# Anti-tumor activity of selinexor in combination with antineoplastic agents in chronic lymphocytic leukemia

**DOI:** 10.1038/s41598-023-44039-0

**Published:** 2023-10-07

**Authors:** Candida Vitale, Valentina Griggio, Maria Todaro, Chiara Riganti, Rebecca Jones, Elia Boccellato, Francesca Perutelli, Francesca Arruga, Tiziana Vaisitti, Dimitar G. Efremov, Silvia Deaglio, Yosef Landesman, Benedetto Bruno, Marta Coscia

**Affiliations:** 1 University Division of Hematology, A.O.U. Città della Salute e della Scienza di Torino, 10126 Turin, Italy; 2 Department of Molecular Biotechnology and Health Sciences, University of Torino, 10126 Turin, Italy; 3 Department of Oncology, University of Torino, 10126 Turin, Italy; 4 Department of Medical Sciences, University of Torino, 10126 Turin, Italy; 5 Molecular Hematology, International Centre for Genetic Engineering and Biotechnology, 34149 Trieste, Italy; 6 Karyopharm Therapeutics, Newton, MA 024559 USA

**Keywords:** Chronic lymphocytic leukaemia, Targeted therapies

## Abstract

Despite recent relevant therapeutic progresses, chronic lymphocytic leukemia (CLL) remains an incurable disease. Selinexor, an oral inhibitor of the nuclear export protein XPO1, is active as single agent in different hematologic malignancies, including CLL. The purpose of this study was to evaluate the anti-tumor effects of selinexor, used in combination with chemotherapy drugs (i.e. fludarabine and bendamustine) or with the PI3Kδ inhibitor idelalisib in CLL. Our results showed a significant decrease in CLL cell viability after treatment with selinexor-containing drug combinations compared to each single compound, with demonstration of synergistic cytotoxic effects. Interestingly, this drug synergism was exerted also in the presence of the protective effect of stromal cells. From the molecular standpoint, the synergistic cytotoxic activity of selinexor plus idelalisib was associated with increased regulatory effects of this drug combination on the tumor suppressors FOXO3A and IkBα compared to each single compound. Finally, selinexor was also effective in potentiating the in vivo anti-tumor effects of the PI3Kδ inhibitor in mice treated with the drug combination compared to single agents. Our data provide preclinical evidence of the synergism and potential efficacy of a combination treatment targeting XPO1 and PI3Kδ in CLL.

## Introduction

Chronic lymphocytic leukemia (CLL) is a very heterogeneous disease, from both the biological and clinical standpoint. Whole-genome sequencing analyses identified recurrent mutations with oncogenic roles^1^, but a univocal founding lesion has not been identified. Besides single-gene mutations, several signaling and transcriptional pathways implicated in cell survival—such as phosphoinositide 3-kinase/protein kinase B (PI3K/Akt), B-cell receptor (BCR)/BTK and nuclear factor kappa-light-chain-enhancer of activated B cells (NF-kB)—are aberrantly activated in CLL, also due to the interactions with the protective tumor microenvironment^2–5^.

The treatment landscape for CLL has been dramatically changing over the last few years, with the availability of small molecules targeting BTK, PI3K and B-cell lymphoma 2 (Bcl-2)^6^. However, despite the therapeutic efficacy of new targeted drugs, treatment of specific subgroups of patients, especially those with high-risk features, remains an unmet clinical need, due to the shorter duration of response and the possible development of resistance^7–9^. The simultaneous targeting of different pro-survival pathways may therefore represent an appealing therapeutic strategy, equally effective for multiple CLL subsets.

Exportin 1 (XPO1) is among the best-characterized nuclear exporters in humans, responsible for the translocation of hundreds of different proteins from the nucleus to the cytoplasm through the nuclear pore complex^10^. Elevated protein levels of XPO1 have been reported in different tumors, especially hematologic malignancies^11^, leading to an altered regulation and localization of cargo proteins and often correlating with poor prognosis^12,13^. In CLL cells, *XPO1* is overexpressed and recurrently mutated^1,11,14^. Moreover, many tumor suppressors and growth regulatory proteins with a known pathogenic role in CLL (e.g. cellular tumor antigen p53, cyclin-dependent kinase inhibitor 1 p21, the effector of Akt signal transduction forkhead box O3 [FOXO3A] and IkappaBα [IkBα], the endogenous inhibitor of NF-kB) are recognized by XPO1 cargo proteins^15^.

Selective inhibitors of nuclear export (SINEs) block XPO1 and their use in vitro and in vivo in a mouse model has confirmed that XPO1 is a valid target in CLL^14^. Selinexor (KPT-330) is a clinically available, orally administered, SINE which affects CLL cells’ viability also in presence of microenvironment-derived pro-survival signals^16^, and elicits a synergistic cytotoxic effect with ibrutinib in vitro^17^. As a single agent, selinexor proved to be safe and showed preliminary anti-cancer activity in phase I studies enrolling patients with relapsed or refractory non-Hodgkin lymphoma, including CLL, acute myeloid leukemia and sarcomas^13,18,19^. A phase I study also showed the safety and some signs of clinical activity of selinexor in combination with ibrutinib in relapsed or refractory patients with CLL or non-Hodgkin lymphomas^20^. Currently, selinexor is approved by the FDA for clinical use in patients affected by relapsed/refractory multiple myeloma and has received accelerated approval in diffuse large B-cell lymphoma^21–23^.

The aim of this study was to identify new selinexor-containing drug combinations that display synergistic mechanisms of cytotoxicity and show anti-tumor efficacy in CLL models, for a possible subsequent transition to a clinical-level experimental phase.

## Results

### Selinexor significantly increases the cytotoxic effect of bendamustine, fludarabine and idelalisib against primary CLL cells

**Figure 1:**
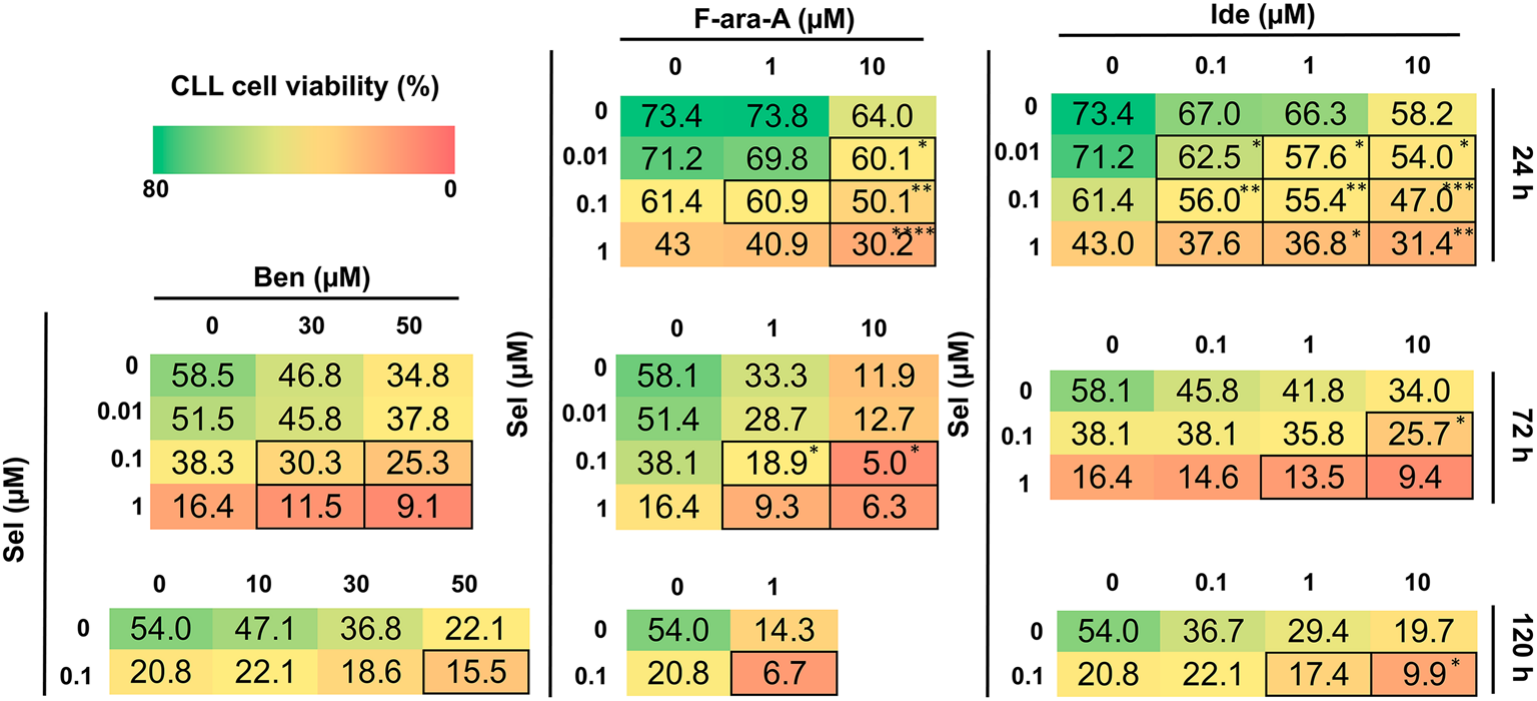
Selinexor (Sel) significantly increases the cytotoxic effect of bendamustine (Ben), fludarabine (F-ara-A) and idelalisib (Ide) against primary CLL cells at multiple concentrations and time points. Heatmaps show the mean viability (% of AnnV-/PI- CLL cells) for 11 samples of patients with CLL that were co-treated in vitro with selinexor + bendamustine, selinexor + fludarabine and selinexor + idelalisib at specific drug concentrations and time points. Cells highlighted with borders indicate the combinations with a CI < 1 (synergistic). Asterisks indicate the combinations which determined a significant reduction in cell viability compared to both single agents, at the corresponding concentration. ****p < 0.0001, ***p < 0.001, **p < 0.01 and *p < 0.05.

**Figure 2:**
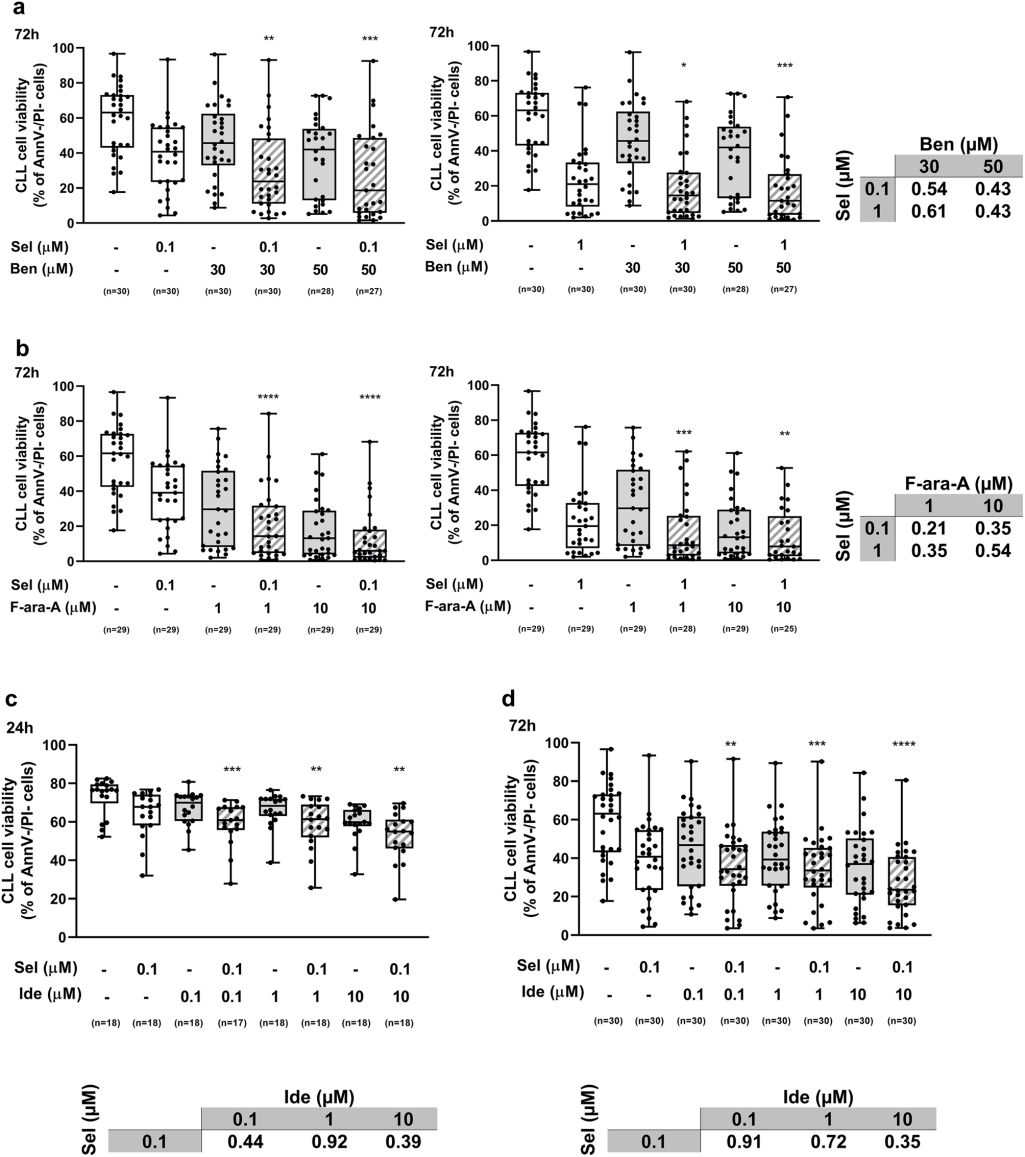
The synergism of selinexor (Sel) + bendamustine (Ben), selinexor + fludarabine (F-ara-A) and selinexor + idelalisib (Ide) is confirmed on an expanded sample cohort. CLL cell viability significantly decreased when samples from an expanded patient cohort were treated with the drug combinations compared to single compounds. (**a**), (**b**) and (**d**) represent 72-h viability, whereas panel (**c**) represents 24-h viability. Box plots represent median value and 25–75% percentiles, whiskers represent minimum and maximum values for each group; each point represents a single sample. CI for all combinations is depicted in tables. Asterisks indicate the combinations which resulted in a significant reduction in cell viability compared to both single agents, at the corresponding concentration. ****p < 0.0001, ***p < 0.001, **p < 0.01 and *p < 0.05.

Primary CLL cells from an exploratory cohort of patients were treated with increasing concentrations of bendamustine, fludarabine or idelalisib, used alone or in combination with selinexor. Viability was evaluated at different time points. Based on the obtained results (Supplementary Fig. 1), the most promising doses and timing for each drug combination (i.e. selinexor + bendamustine, selinexor + fludarabine and selinexor + idelalisib) were selected and tested on an expanded cohort of 11 patients (Fig. 1). Overall, the most effective results in terms of cytotoxicity and drug synergism were obtained after 72-h treatment with selinexor + bendamustine and selinexor + fludarabine, and after 24- and 72-h treatment with selinexor + idelalisib. For all time points and concentrations, we also demonstrated the absence of selinexor cytotoxicity towards non-neoplastic CD3+ and CD14+ cells (data not shown). Based on the difference between the viability after combination and single-agent treatment, and on the combination index (CI) results, we further selected specific drug combinations and time points, and extended the analyses to an expanded cohort of primary CLL samples (Fig. 2). We confirmed a significant decrease in CLL cell viability when samples were treated with drug combinations compared to single compounds. We observed a synergistic cytotoxic effect of selinexor 0.1 µM + bendamustine 30 µM and 50 µM, after 72-h culture (Fig. 2a). At the same time point, combination analysis for selinexor + fludarabine showed a strong synergism for selinexor 0.1 µM + fludarabine 1 µM, a synergism for selinexor 0.1 µM + fludarabine 10 µM, and a synergism for selinexor 1 µM + fludarabine 1 and 10 µM (Fig. 2b).

**Figure 3:**
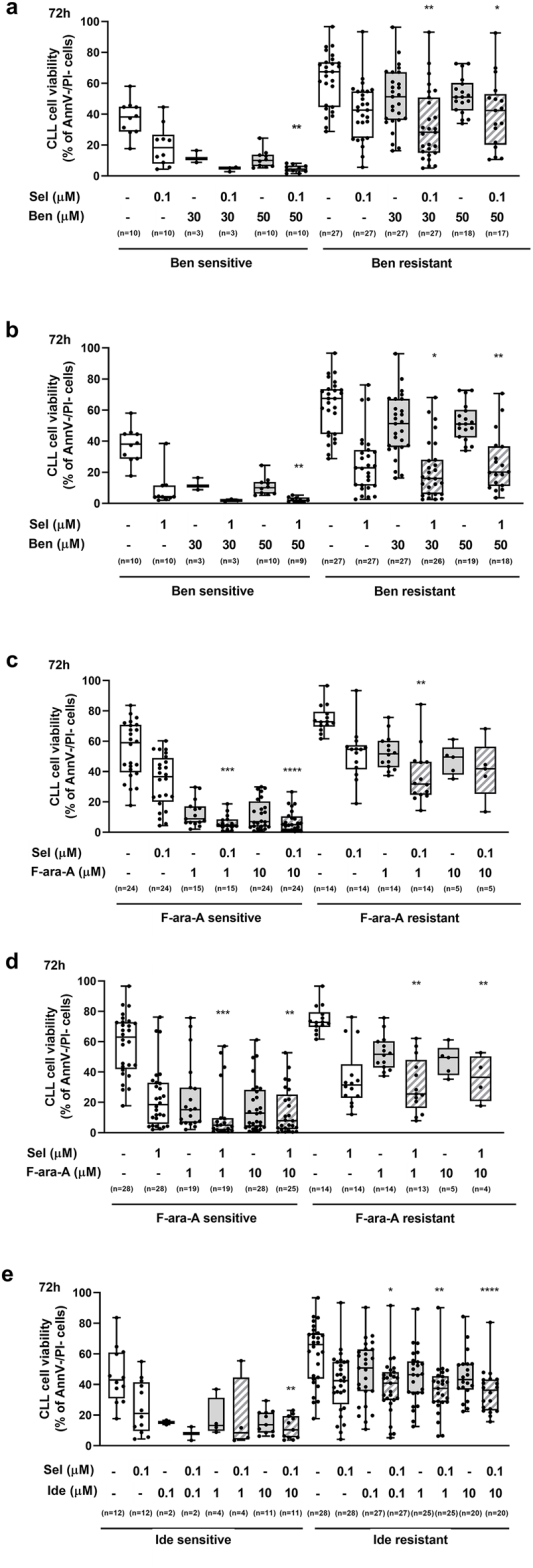
Selinexor (Sel) is effective in restoring drug-induced cytotoxicity. CLL samples were categorized as drug-sensitive or drug-resistant if their normalized cell viability after the specific drug treatment was ≤ or > 0.5, respectively. In the CLL samples characterized by in vitro resistance to bendamustine (Ben) (**a**,**b**), fludarabine (F-ara-A) (**c**,**d**) or idelalisib (Ide) (**e**,**f**), the addition of selinexor significantly increased drug-induced cytotoxicity at the majority of tested concentrations. Box plots represent median value and 25–75% percentiles for 72-h viability, whiskers represent minimum and maximum values for each group; each point represents a single sample. Asterisks indicate the combinations determining a significant reduction in cell viability compared to both single agents, at the corresponding concentration. ****p < 0.0001, ***p < 0.001, **p < 0.01 and *p < 0.05.

The combinations selinexor 0.1 µM + idelalisib 0.1 µM, 1 µM and 10 µM resulted synergistic or strongly synergistic both at 24-h and 72-h time points (Fig. 2c,d). Interestingly, in the cohorts of CLL samples that were characterized by in vitro resistance to single-agent bendamustine, fludarabine or idelalisib (i.e. samples showing a normalized cell viability after single-agent-treatment > 0.5, Supplementary Fig. 2), the addition of selinexor was effective in restoring drug-induced cytotoxicity (Fig. 3).

### Selinexor-based combinations differently affect CLL cell viability in specific molecular subgroups

It has been previously reported that CLL cells characterized by Immunoglobulin Heavy chain Variable region genes (IGHV) mutated (M) status and by the presence of del(17p) are less sensitive to SINE KPT-185 and to selinexor^14,16^. In line with previous data, our results show that single-agent selinexor is less effective in inducing cytotoxicity towards CLL cells carrying del(17p) and/or *TP53* mutations (*TP53* disrupted [*TP53*^*dis*^] subset). By contrast, we did not observe a difference in selinexor-induced cytotoxicity between IGHV M and unmutated (UM) CLL cells (Supplementary Fig. 3). When selinexor was used in combination with bendamustine, fludarabine or idelalisib, there was no significant difference in 72-h cell viability based on IGHV mutational status (Supplementary Fig. 4). By contrast, the cytotoxic effect of selinexor in combination with chemotherapy drugs was dependent on the *TP53* status. Indeed, *TP53*^dis^ samples exposed to selinexor + bendamustine, selinexor + fludarabine or selinexor + idelalisib were less susceptible to drug-induced cytotoxicity compared to *TP53* wild type (*TP53*^wt^) (Supplementary Fig. 5).

### Selinexor effectively counteracts the stromal cell-induced resistance of CLL cells to bendamustine, fludarabine and idelalisib

**Figure 4:**
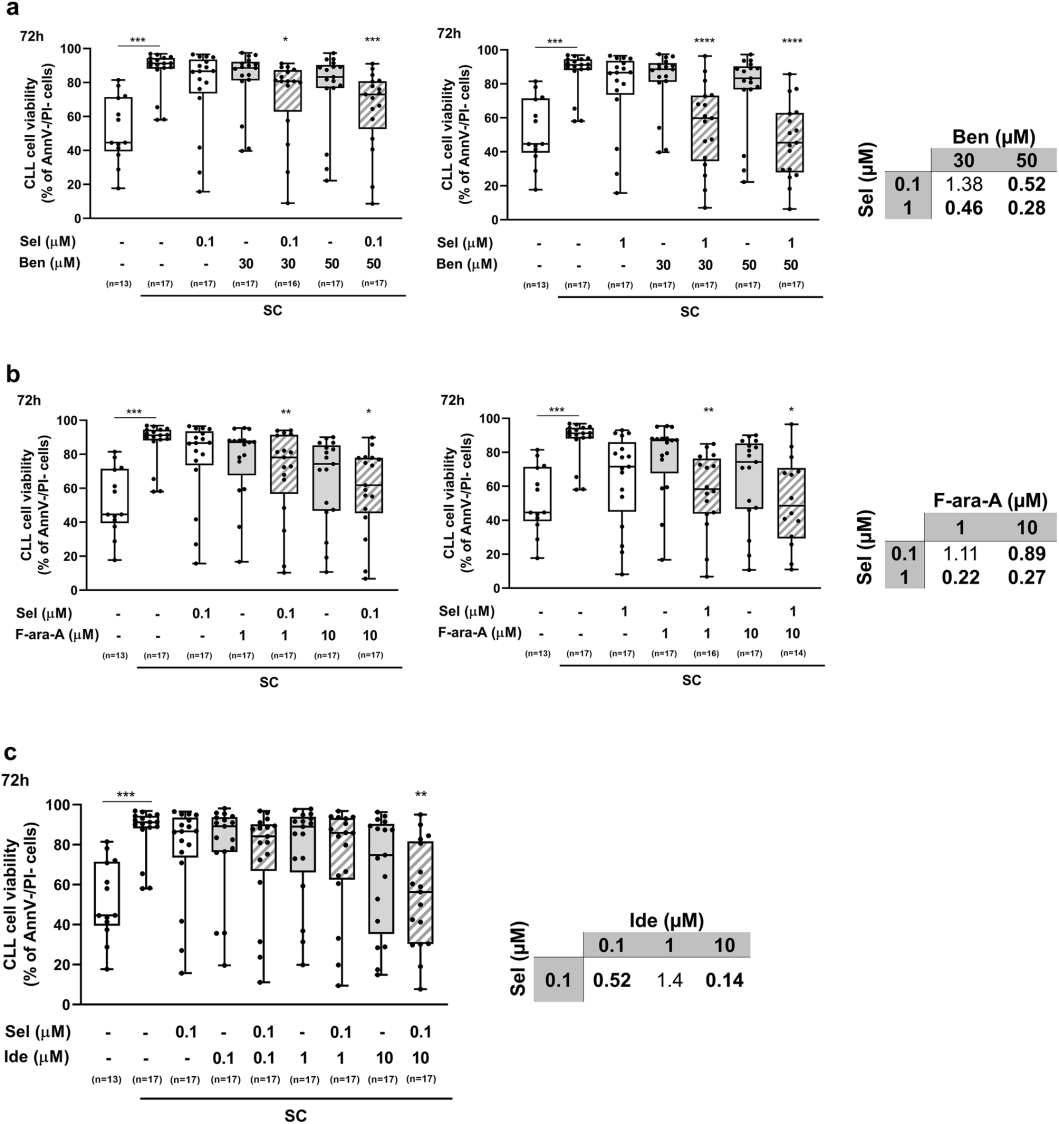
Selinexor (Sel) exerts synergistic cytotoxic activity with bendamustine (Ben), fludarabine (F-ara-A) or idelalisib (Ide) towards CLL cells co-cultured with stromal cells. CLL cells were co-cultured with M2-10B4 stromal cells (SC) and exposed to selinexor, bendamustine, fludarabine and idelalisib (Ide), at specified concentrations for 72 h. Box plots represent median value and 25–75% percentiles for viability, whiskers represent minimum and maximum values of each group; each point represents a single sample. CI for all combinations is depicted in tables. Asterisks indicate the combinations which determined a significant reduction in cell viability compared to both single agents, at the corresponding concentration. ****p < 0.0001, ***p < 0.001, **p < 0.01 and *p < 0.05.

Stromal cells protect CLL cells from spontaneous apoptosis and drug-induced cytotoxicity^24–26^. Previously reported data show that *s*ingle-agent selinexor exerts a cytotoxic effect toward primary CLL cells also in the presence of the pro-survival effect exerted by stromal cells^16,17^. Therefore, we next wanted to evaluate the most effective drug combinations for their ability to reverse the protection exerted by stromal cells toward drug-induced cytotoxicity. First, a direct cytotoxic effect of each drug combination toward the stromal cell component was excluded (data not shown). The protective effect of stromal cells was confirmed by the high values of 72-h viability of CLL cells co-cultured with stromal cells, both in the absence and in the presence of selinexor, bendamustine, fludarabine and idelalisib used as single agents. By contrast, most drug combinations significantly reduced the viability of leukemic cells co-cultured with stromal cells, thus confirming their ability to at least partially hamper the protective effect exerted by stromal cells toward drug-induced cytotoxicity (Fig. 4). Interestingly, most of the tested drug combinations also showed synergism in co-culture system, with CI < 1.

### The cytotoxicity of the selinexor and idelalisib combination is mediated by IkBα and FOXO3A nuclear retention

**Figure 5:**
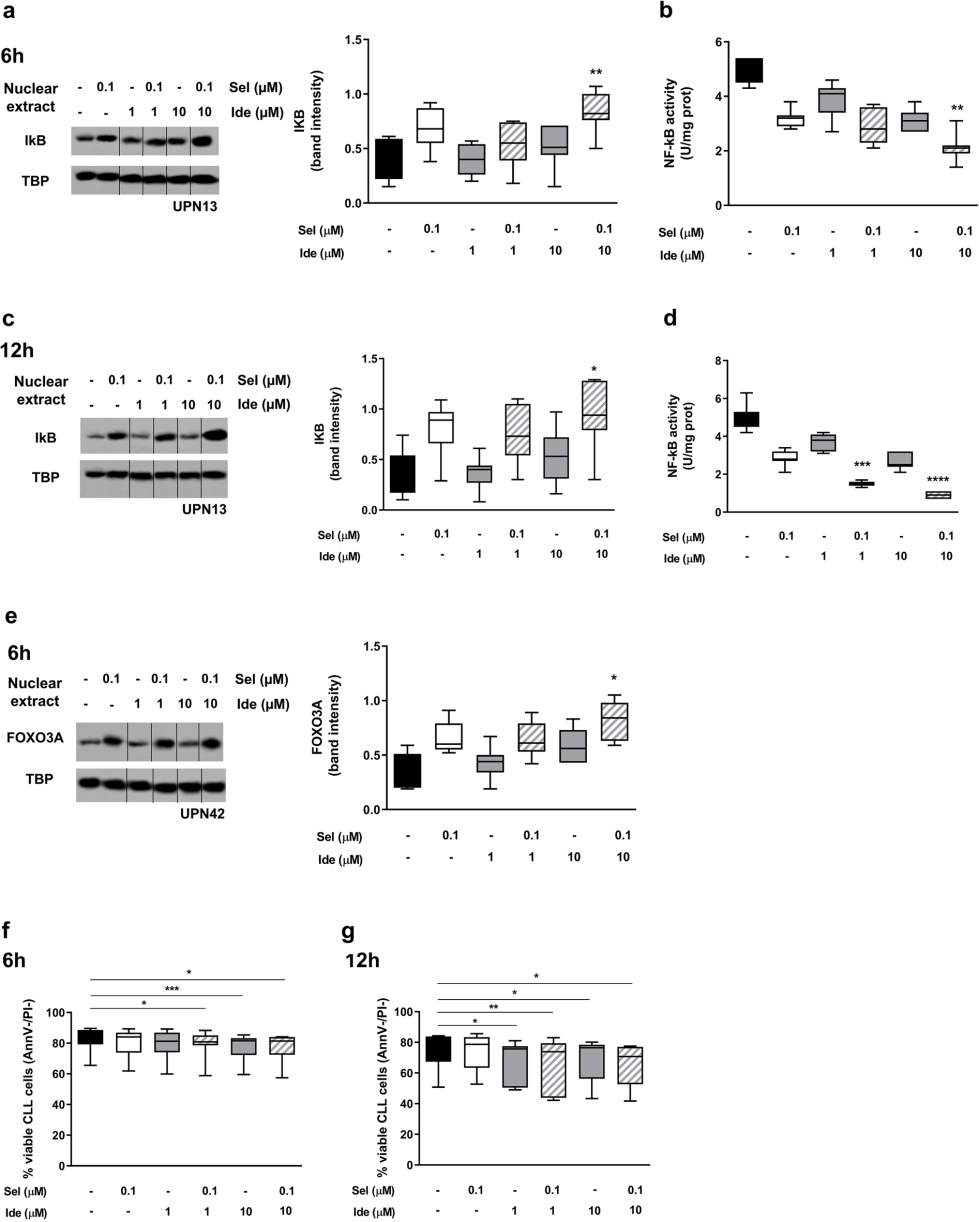
The effects of the selinexor (Sel) + idelalisib (Ide) combination are mediated by IkBα and FOXO3A. Primary CLL cells were exposed to selinexor and idelalisib at specified concentrations. IkBα nuclear amount (**a**,**c**), NF-kB activity (**b**,**d**) and cell viability (**f**,**g**) were evaluated after 6 and 12 h. FOXO3A nuclear amount (**e**) was evaluated after 6 h. In (**a**), (**c**) and (**e**) a representative blot (with relative Unique Patient Number, UPN13 and UNP42), together with the corresponding cumulative band intensity data of 7 independent experiments, respectively, is shown. Box and whiskers plots show median values, 25–75% percentiles, and minimum and maximum values for each group. Repositioned gel lanes are indicated by vertical lines. Asterisks indicate the combinations which determined a significant effect compared to both single agents, at the corresponding concentration. ****p < 0.0001, ***p < 0.001, **p < 0.01 and *p < 0.05.

To explore the molecular basis of the synergistic cytotoxic effect of selinexor and idelalisib, we investigated the effects of XPO1 inhibition in combination with PI3Kδ inhibition on the nuclear retention of IkBα and FOXO3A, which are intracellular mediators functionally regulated by the PI3K/Akt pathway and are known cargo proteins of XPO1. Of note, for this set of experiments, we only selected the concentrations of idelalisib that exhibited an inhibitory effect on Akt phosphorylation (i.e. 1 and 10 µM, Supplementary Fig. 6). A schematic representation of the role of XPO1, XPO1 inhibition, PI3K pathway activity and PI3K inhibition on the NF-kB inhibitor IkBα and on the tumor suppressor FOXO3A cellular localization is depicted in Supplementary Fig. 7^14,27,28^. After 6-h culture, the combination of selinexor and idelalisib at different concentrations induced a nuclear accumulation of IkBα (Fig. 5a), which was paralleled by a significant decrease in NF-kB activity (Fig. 5b) in primary CLL cells. The nuclear retention of IkBα was confirmed also at the 12-h time point when we observed an even more pronounced inhibition of the transcriptional activity of NF-kB (Fig. 5c,d). Similarly, also FOXO3A nuclear accumulation was promoted after 6-h exposure of CLL cells to the combination selinexor + idelalisib (Fig. 5e). Interestingly, all these molecular effects were not attributable to a reduction in cell viability, which was only negligibly decreased after short-term (i.e. 6-h and 12-h) exposure to the drug combination (Fig. 5f,g).

### In vivo evaluation of the activity of selinexor in combination with bendamustine or acalisib

**Figure 6:**
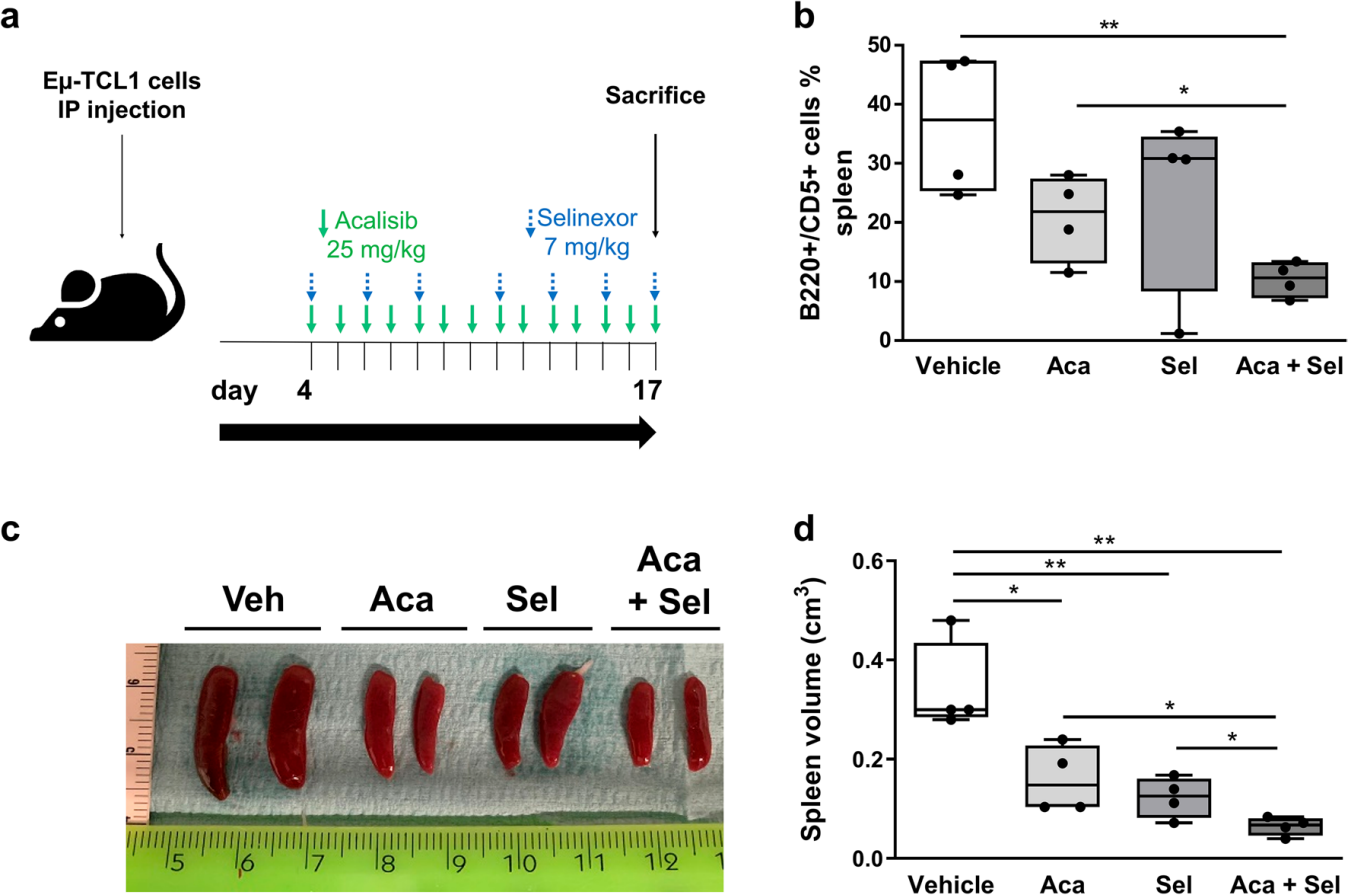
Selinexor (Sel) in combination with acalisib (Aca) is effective in reducing the tumor burden in vivo. Mice transplanted with the Eµ-TCL1-derived leukemia were randomly treated with selinexor and acalisib, used as single agents or in combination, or with a vehicle. A schematic outline of in vivo experiments is depicted in (**a**). (**b**) Shows the percentage of leukemic cells (CD5+/B220+ cells) in the spleen of mice transplanted with the Eµ-TCL1-derived leukemia after 2 weeks of treatment. (**c**) shows the macroscopic view of the spleens from two representative animals for each treatment group, and (**d**) shows the spleen volume for all treated animals. In (**b**) and (**d**) box and whiskers plots represent median values, 25–75% percentiles, and minimum and maximum values for each group; each point represents a single sample. **p < 0.01 and *p < 0.05.

To further corroborate in vitro data and the anti-tumor effect of selinexor-based combinations, we exploited a murine model derived from the engraftment of Eµ-TCL1 leukemic cells into syngeneic mice. In these experiments we tested selinexor in combination with bendamustine and with acalisib—a second generation PI3Kδ inhibitor whose pharmacokinetic profile renders it more suitable than idelalisib for in vivo experiments. After 2-week treatment, the combination of selinexor and bendamustine was effective in reducing the tumor burden in the splenic compartment (Supplementary Fig. 8). In this setting, bendamustine and selinexor, used alone or in combination, induced a decrease in the spleen volume of treated mice when compared to vehicles. However, single-agent bendamustine already exerted a strong anti-tumor effect thus masking the possible beneficial effect of adding selinexor to the treatment regimen. Conversely, selinexor clearly potentiated the anti-tumor activity of acalisib (Fig. 6a–d). In fact, mice treated with selinexor + acalisib showed a significantly lower infiltration by leukemic cells and a significant reduction of the volume of the spleen compared to mice treated with each agent alone.

## Discussion

In this study we evaluated the cytotoxic effects of the XPO1 inhibitor selinexor, used in combination with chemotherapy drugs or the PI3Kδ inhibitor idelalisib in CLL. We showed that selinexor significantly increases the cytotoxic effect of fludarabine, bendamustine and idelalisib against primary CLL cells in vitro, exerting synergism at multiple concentrations, also when leukemic cells are cultured in the presence of the protective effect of stromal cells. Selinexor in combination with idelalisib affected NF-kB and PI3K/Akt pathways, and the synergic cytotoxic effect of the combination was mediated by IkBα and FOXO3A nuclear retention. Finally, in vivo, selinexor was able to increase the anti-tumor effect of the PI3Kδ inhibition.

A curative approach for CLL has not yet been identified, and the need to overcome the mechanisms of drug resistance and to achieve better efficacy results constitutes the rationale for investigating novel drug combinations. It has been previously shown that, in cell lines derived from different tumors, selinexor significantly affects the mechanisms of DNA damage repair and synergistically increases the cytotoxicity of chemotherapy agents exerting DNA damaging effects^29^. Additionally, it has been shown that selinexor inhibits the activation of pathways downstream to BCR^16^, thus synergizing with the BTK inhibitor ibrutinib in CLL^17^. This dual blocking of BTK by selinexor plus ibrutinib has already shown to result in promising clinical activity^20^.

In this paper, we tested selinexor in combination with two different chemotherapy agents used for the treatment of CLL—fludarabine and bendamustine—and also with the targeted PI3Kδ inhibitor idelalisib. PI3K inhibitors—among which the δ subunit inhibitor idelalisib has been the first and most widely tested, also at a clinical level—are another class of drugs that induce the apoptosis of CLL cells through the dual inhibition of the BCR signaling and of the chemokine receptor-dependent activation of Akt, that are key mechanisms in promoting microenvironment-induced survival of leukemic cells^2,3^. Our data show that selinexor synergizes with both standard chemotherapy agents and idelalisib in affecting primary CLL cells viability in vitro. Of note, selinexor could overcome the intrinsic in vitro resistance of leukemic cells to fludarabine, bendamustine and idelalisib, as shown by the significant reduction of cell viability after exposure of CLL cells to drug combinations containing selinexor compared to each single-agent treatment. Even more interestingly, our combination experiments showed that the addition of selinexor allows to lower the in vitro cytotoxic concentrations of each partner drug (i.e. fludarabine, bendamustine or idelalisib) much below the Cmax achieved with doses currently administered to patients^30^. From the clinical standpoint, this observation may open the way to the testing of combination regimens containing reduced doses of each drug, thus limiting the potential side effects.

In CLL, drug sensitivity is affected not only by intrinsic features of the leukemic cells, such as the presence of *TP53* abnormalities and UM IGHV status, but also by the interactions of neoplastic cells with the surrounding microenvironment^26,31–33^. Interestingly, we observed that selinexor—when used in combination with fludarabine, bendamustine or idelalisib—was capable to partially overcome the protective effect exerted by stromal cells toward the cytotoxicity of each single drug, confirming the synergism of all selinexor-containing combinations also in the co-culture system.

With the idea of pursuing the design of a chemo-free regimen, we next investigated the molecular mechanisms underlying the synergistic activity of the combination selinexor + idelalisib. The PI3K/Akt signaling is an important regulator of both FOXO3A and IkBα tumor suppressors. Akt phosphorylates FOXO3A and leads to its cytoplasmic sequestration and degradation, thus reducing its nuclear accumulation and transcriptional activity^28,34^. Notably, it has already been reported that the tumor suppressor FOXO3A is constitutively phosphorylated and inactive in mantle cell lymphoma cells, whose viability is indeed effectively impaired by the functional re-activation of FOXO3A^35^. In addition, Akt can phosphorylate and activate IKK, which in turn phosphorylates IkBα leading to its proteasomal degradation and to the activation and nuclear translocation of the tumor-promoting transcription factor NF-kB^36^. Accordingly, our data show that the exposure of CLL cells to PI3K-inhibiting concentrations of idelalisib reduces the activity of NF-kB and upregulates the nuclear expression levels of FOXO3A in a dose-dependent manner. In addition to PI3K/Akt, also XPO1 contributes to the regulation of FOXO3A and IkBα activity by affecting their nuclear-cytoplasmic shuttling^15^. In line with previous data^28,37^, our results show that selinexor-mediated inhibition of XPO1 induces the nuclear retention of FOXO3A and IkBα in primary CLL cells. Notably, the combined exposure of CLL cells to selinexor and idelalisib further increased the nuclear concentrations of FOXO3A and significantly reduced the nuclear activity of NF-kB compared to each single agent, thus explaining from a molecular standpoint, the synergism of this drug combination. It has already been reported that selinexor has single-agent activity in ibrutinib-resistant CLL in vitro, suggesting it may be effective in ibrutinib-resistant CLL patients^17^. Based on this observation and on our findings, it might be interesting, as a future perspective, to investigate whether the synergism of the combination selinexor + idelalisib is maintained also toward leukemic cells isolated from the small but clinically relevant subgroup of patients showing resistance to a sequential or combined treatment with BTK and BCL2 inhibitors.

Currently, one major limitation of the clinical applicability of the combination selinexor + idelalisib might be the concern linked to the recent alert launched by the FDA following the suspicion of an overall reduced life expectancy in patients receiving PI3K inhibitors within randomized clinical trials^38^. This increase in mortality might be due to drug-related toxicities leading, on one hand, to a high rate of deaths directly linked to adverse events and, on the other hand, to the frequent treatment interruptions importantly affecting patients’ outcome. Despite the reported toxicities, several PI3K inhibitors (e.g. idelalisib, duvelisib, umbralisib and copanlisib) had been tested for the treatment of CLL and other lymphoproliferative disorders—including indolent and aggressive B-cell lymphomas—showing an uncontroversial clinical efficacy^39–43^. Our data demonstrate a synergism of selinexor with idelalisib, which is further corroborated by the demonstration of interconnected molecular mechanisms and of an in vivo anti-tumor efficacy. Altogether, these observations might support the design of phase I clinical trials where the PI3K inhibitor—thanks to the combination with selinexor—is administered at lower doses than those previously approved for patients’ treatment, or with an intermittent dosing regimen. Treatment with different schedule or dosage of PI3K inhibitors might have the potential of limiting drug-related toxicities, thus allowing not to completely give up a potentially effective drug class.

## Methods

### Human primary samples

CLL patients were diagnosed according to International Workshop on CLL-National Cancer Institute (IWCLL/NCI) guidelines^44^. A total of 45 patients (Supplementary Table 1) were included in the study after their written informed consent, in accordance with the Declaration of Helsinki and approval by the local Institutional Review Board (Comitato Etico Interaziendale A.O.U. Città della Salute e della Scienza di Torino-A.O. Ordine Mauriziano-A.S.L. Città di Torino. Ref. CS/570-1211/2015 and Ref. CS2/1123-337/2019). Patients were either untreated or off therapy for at least 12 months before sampling of peripheral blood (PB) for the experiments (Supplementary Table 1).

Samples were characterized for IGHV mutational status and sequences with deviations of < 2% or ≥ 2% from the germline IGHV sequence were considered IGHV UM or M, respectively^45^. Del(17p) in CLL cells was assessed by *fluorescence *in situ* hybridization* and the presence of *TP53* gene mutations was evaluated by Sanger sequencing. CLL patients with mutation of the *TP53* gene and/or del(17p) were considered *TP53*^dis^, whereas samples without *TP53* mutation nor del(17p) were included in the *TP53*^wt^ subset.

### Culture and co-culture conditions

Peripheral blood mononuclear cells (PBMC) were isolated by density gradient centrifugation on Pancoll human (#cP04-60500, PanBiotech, Aidenbach, Germany). PBMC were stained with anti-CD19 PerCP Vio700 (#130-113-171) and anti-CD5-APC (#130-118-364) antibodies (Miltenyi Biotec, Bologna, Italy), and were used without further manipulation when they contained more than 90% of CLL cells (CD19+/CD5+). When CLL cells were ≤ 90% they were purified by immunomagnetic bead method (B-CLL Cell Isolation Kit, #130-103-466 Miltenyi Biotec). CLL cells were cultured in 96- or 24-well plates (#3799 and #CLS3527, Costar, Cambridge, MA, USA) (2x10^5^ cells/well or 10^6^ cells/well) at 37 °C in a 5% CO_2_ humidified incubator. The standard culture medium was RPMI 1640 (#21875-034, Gibco, Thermo Fisher Scientific, Monza, Italy) supplemented with 10% FCS (#10270-106, Gibco), 2 mM l-glutamine (#25030081 Gibco), 100 U/ml penicillin and 100 μg/ml streptomycin (#15140122, Gibco).

Primary CLL cells were exposed in vitro to increasing concentrations of selinexor (KPT-330, kindly provided by Karyopharm Therapeutics, Newton, MA) (0.01 μM, 0.1 μM, 1 μM or 10 μM), fludarabine (2-Fluoroadenine-9-b-D-arabinofuranoside, #F2773 Sigma-Aldrich, Milano, Italy) (0.01 μM, 0.1 μM, 1 μM or 10 μM), bendamustine (#S5939 Selleckchem, Munich, Germany) (1 μM, 3 μM, 10 μM, 30 μM or 50 μM) and idelalisib (CAL101, #S2226 Selleckchem) (0.01 μM, 0.1 μM, 1 μM or 10 μM). The drugs were tested at all concentrations either as single agent or in combination (selinexor + fludarabine, selinexor + bendamustine and selinexor + idelalisib). For combination experiments, all drugs were incubated at the same time. In selected experiments, whole PBMC were used, at the same culture conditions, to test drugs toxicity against non-neoplastic cells. The M2-10B4 murine stromal cell line (ATCC # CRL-1972) was used for co-culture experiments.

### Cell viability assay

Cell viability was evaluated at specified timepoints by flow cytometry with Annexin-V/Propidium Iodide (AnnV/PI) staining with the MEBCYTO-Apoptosis Kit (#4700, MBL Medical and Biological Laboratories, Naka-ku Nagoya, Japan) according to the manufacturer’s instructions. Viability of non-neoplastic cells (i.e. CD3+ and CD14+ cells) was also assessed, evaluating AnnV- cells gating on CD3+ and CD14+ cells (anti-human CD3-PerCP #300326, Biolegend and anti-human CD14-PE # 130-113-147, Miltenyi Biotec). Flow cytometry samples were acquired with FACSCalibur and data were analyzed by CELLQuest software (Becton Dickinson, Mountain View, CA, USA) and FlowJo software (Tree Star, Inc, Ashland, OR, USA). Normalized cell viability was defined as the ratio between the percentage of viable CLL cells (AnnV−/PI−) cultured in presence of the drug of interest and the percentage of viable CLL cells that were left untreated for the same time interval. Samples were considered resistant when the normalized cell viability after the specific drug treatment was > 0.5.

### Western Blots and NF-kB activity

Cytosolic and nuclear protein extracts were obtained using the Nuclear Extract Kit (#40410, Active Motif, Active Motif, Carlsbad, CA, USA) following the manufacturer’s instructions. Lysates were resolved by SDS‐PAGE and transferred to nitrocellulose membranes (Bio-Rad, Hercules, CA, USA). The following monoclonal antibodies were used: anti-p(Ser 473)Akt (Millipore); anti-Akt (Millipore); anti-IkBα (Cell Signaling Technology, #4812) anti-FOXO3A (Cell Signaling Technology, #12829S). Antibodies anti-TUBULIN (#sc-5274, Santa Cruz Biotechnology, Dallas, TX, USA) and anti-TATA-box binding protein (TBP, #sc-56795, Santa Cruz Biotechnology) were used as control of equal protein loading for cytosolic and nuclear fractions. Secondary peroxidase-conjugated antibodies (#1662408EDU, Bio-Rad Laboratories) were used. Blot images were acquired with a ChemiDocTM Touch Imaging System device (Bio-Rad Laboratories). Original blots are presented in Supplementary Figs. 9 and 10. The ImageJ software (NIH, Bethesda, MD, USA) was used to perform densitometric analysis of western blot band intensity. For graph representation, the band intensity of the proteins of interest was normalized on the correspondent housekeeping proteins. NF-kB activity was evaluated by the TransAM Flexi NF-kB Family assay kit (#43298, Active Motif).

### Animal experiments

C57BL/6 Eμ-TCL1 mice were maintained in specific pathogen-free animal facilities and treated in accordance with European Union guidelines, Institutional Animal Care and Use Committee, and ARRIVE guidelines. The in vivo experiments were approved by the Italian Ministry of Health (Authorization n. 47/2018-PR). Splenic leukemic cells from Eμ-TCL1 mice (10^7^ cells) were injected i.p. into syngeneic C57BL/6 mice^46,47^, and experiments were performed with 4 mice groups. Engrafted mice were randomly assigned to treatment groups: vehicle, selinexor, bendamustine, acalisib, selinexor + bendamustine, selinexor + acalisib. Leukemic mice were treated after 3 or 4 days from the injection. Selinexor (kindly provided by Karyopharm Therapeutics, Newton, MA, USA) was administered at a dose of 7 mg/kg, formulated in Plasdone PVP K29/32 and Poloxamer Pluroniv F-68 (kindly provided by Karyopharm Therapeutics, Newton, MA, USA), three times a week by oral gavage. Bendamustine was administered at a dose of 24 mg/kg, formulated in 1% DMSO, 30% PEG300, 1% Tween 80 and saline solution, once a week i.v. The PI3Kδ inhibitor acalisib (GS-9820, # HY-12644, MedChemExpress, Monmouth Junction, NJ, USA) was used for animal experiments due to its isoform specificity and better pharmacokinetic properties than idelalisib in the mouse^48^, and was administered at a dose of 25 mg/kg, formulated in in 10% DMSO, 40% PEG300, 5% Tween 80 and saline solution, by daily oral gavage. Mice were euthanized at after 2 weeks of treatment by CO_2_ inhalation followed by cervical dislocation. The percentage of TCL1 cells was evaluated in PB, bone marrow and spleen compartments using anti-mouse CD45R/B220 PE-Cy7 and anti-mouse CD5 BB515 (#552772 and #565504, BD Biosciences, Allschwil, Switzerland) by flow cytometry (FACS Celesta, BD Biosciences). Spleen volume was also measured.

### Statistical analysis

GraphPad Prism (San Diego, CA, USA) was used to perform statistical analyses (using paired t-test or Wilcoxon matched-pairs signed rank test, or unpaired t-test or Mann–Whitney test, based on the data distribution). A *p* value < 0.05 was considered significant. For synergism analyses, the CI was determined using CompuSyn software (ComboSyn, Inc.). CI values < 0.3, < 1, = 1 and > 1 indicate strong synergism, synergism, additivity and antagonism, respectively.

### Supplementary Information

Supplementary Information.

## Data Availability

The data that support the findings of this study are available from the corresponding author, but restrictions apply to the availability of these data, which were used under license for the current study, and so are not publicly available. Data are however available from the corresponding author upon reasonable request and with permission of Karyopharm Therapeutics Inc.
