# Supplementary material for: Anti-tumor activity of selinexor in combination with antineoplastic agents in chronic lymphocytic leukemia
Source: Sci Rep. 2023 Oct 7;13:16950. doi: 10.1038/s41598-023-44039-0 (PMC10560255; doi:10.1038/s41598-023-44039-0)
Supplement: Supplementary file 1 — Supplementary Information. [file 41598_2023_44039_MOESM1_ESM.docx]

**Anti-tumor activity of selinexor in combination with antineoplastic agents in chronic lymphocytic leukemia**

**Candida Vitale**1,2**+, Valentina Griggio**1,2**+, Maria Todaro**1,2**, Chiara Riganti**3**, Rebecca Jones**1,2**, Elia Boccellato**1,2**, Francesca Perutelli**1,2**, Francesca Arruga**4**, Tiziana Vaisitti**4**, Dimitar G. Efremov**5**, Silvia Deaglio**4**, Yosef Landesman**6**, Benedetto Bruno**1,2 **and Marta Coscia**1,2***.**

1University Division of Hematology, A.O.U. Città della Salute e della Scienza di Torino, Torino, 10126, Italy

2Department of Molecular Biotechnology and Health Sciences, University of Torino, Torino, 10126, Italy

3Department of Oncology, University of Torino, Torino 10126, Italy

4Department of Medical Sciences, University of Torino, Torino, 10126, Italy

5Molecular Hematology, International Centre for Genetic Engineering and Biotechnology, Trieste, 34149, Italy

6Karyopharm Therapeutics, Newton, MA, 024559, USA.

[*marta.coscia@unito.it](mailto:*marta.coscia@unito.it)

+these authors contributed equally to this work

**Supplementary Table 1. Summary of genetic patient characteristics.**

| Unique Patient Number (UPN) | Del(13q) | Del(11q) | Tris(12) | Del(17p) | FISH category | *TP53*^mut^ | *TP53*^dis^ | IGHV mutational status | Previous treatment  (interval between last treatment and sampling) |
| --- | --- | --- | --- | --- | --- | --- | --- | --- | --- |
| UPN01 | NA | NA | NA | NA | NA | NA | NA | NA | None |
| UPN02 | no | no | no | no | No | wt | wt | M | None |
| UPN03 | yes | no | no | no | Del(13q) | wt | wt | M | None |
| UPN04 | yes | no | no | no | Del(13q) | wt | wt | M | None |
| UPN05 | no | no | no | yes | Del(17p) | NA | Dis | UM | Chl (4 years) |
| UPN06 | yes | no | no | no | Del(13q) | wt | wt | M | None |
| UPN07 | no | no | no | no | No | wt | wt | M | Chl, fludarabine (8 years) |
| UPN08 | yes | no | no | no | Del(13q) | wt | wt | M | CVP, chl, fludarabine, alemtuzumab (3 years) |
| UPN09 | no | no | no | no | No | wt | wt | UM | None |
| UPN10 | NA | NA | NA | NA | NA | NA | NA | M | Chl (7 years) |
| UPN11 | no | no | yes | no | Tris(12) | wt | wt | M | None |
| UPN12 | NA | NA | NA | no | NA | mut | Dis | M | None |
| UPN13 | yes | no | no | no | Del(13q) | wt | wt | UM | None |
| UPN14 | no | no | yes | no | Tris(12) | wt | wt | UM | None |
| UPN15 | no | yes | yes | no | Del(11q) | wt | wt | UM | None |
| UPN16 | yes | no | no | no | Del(13q) | wt | wt | M | None |
| UPN17 | yes | no | no | no | Del(13q) | wt | wt | M | Rituximab + chl (4 years) |
| UPN18 | yes | no | no | yes | Del(17p) | wt | Dis | M | Chl, FCR, rituximab + cyclophosphamide, rituximab + chl, CVP, BR (2 years) |
| UPN19 | yes | no | no | yes | Del(17p) | mut | Dis | UM | None |
| UPN20 | no | no | no | no | No | wt | wt | UM | None |
| UPN21 | no | no | yes | no | Tris(12) | wt | wt | UM | Chl, rituximab + chl (1 year) |
| UPN22 | NA | NA | NA | NA | NA | NA | NA | UM | None |
| UPN23 | yes | yes | no | yes | Del(17p) | wt | Dis | UM | Chl, BR (5 years) |
| UPN24 | yes | no | no | no | Del(13q) | wt | wt | UM | None |
| UPN25 | no | no | no | yes | Del(17p) | mut | Dis | UM | R-CHOP, alemtuzumab, BR, rituximab + chl, rituximab, ofatumumab, chl + rituximab (3 years) |
| UPN26 | no | no | no | yes | Del(17p) | mut | Dis | M | None |
| UPN27 | yes | no | no | no | Del(13q) | wt | wt | M | Fludarabine + cyclophosphamide (7 years) |
| UPN28 | no | no | no | no | No | NA | NA | NA | Chl (2 years) |
| UPN29 | no | yes | no | no | Del(11q) | wt | wt | UM | FCR (3 years) |
| UPN30 | yes | yes | no | no | Del(11q) | wt | wt | M | FCR (8 years) |
| UPN31 | yes | no | no | no | Del(13q) | wt | wt | UM | None |
| UPN32 | no | no | yes | no | Tris(12) | NA | wt | UM | Chl, bendamustine, cyclophosphamide (1 year) |
| UPN33 | yes | no | no | yes | Del(17p) | mut | Dis | UM | FCR (6 years) |
| UPN34 | yes | no | no | no | Del(13q) | wt | wt | M | None |
| UPN35 | no | no | no | no | No | wt | wt | NA | None |
| UPN36 | yes | no | no | no | Del(13q) | NA | NA | M | None |
| UPN37 | yes | no | no | no | Del(13q) | wt | wt | M | None |
| UPN38 | yes | no | no | no | Del(13q) | NA | NA | M | Pentostatine + cyclophosphamide + ofatumumab (7 years) |
| UPN39 | no | no | no | no | No | NA | NA | M | None |
| UPN40 | NA | NA | NA | NA | NA | NA | NA | NA | None |
| UPN41 | no | no | no | no | No | wt | wt | M | None |
| UPN42 | no | yes | no | no | No | wt | wt | UM | None |
| UPN43 | NA | NA | NA | NA | NA | NA | NA | NA | Chl, BR, idelalisib + rituximab (2 years) |
| UPN44 | yes | no | no | no | No | wt | wt | UM | BR (4 years) |
| UPN45 | no | no | yes | no | No | no | wt | UM | None |

Abbreviations: UPN, Unique patient number; NA, not available; IGHV, Immunoglobulin heavy chain variable region genes; M, mutated; UM, unmutated; Chl, chlorambucil; CVP, cyclophosphamide, vincristine and prednisone; FCR, fludarabine, cyclophosphamide and rituximab; BR, bendamustine + rituximab, chlorambucil; R-CHOP, rituximab, cyclophosphamide, doxorubicin, vincristine and prednisone.

**Supplementary Figure 1.**


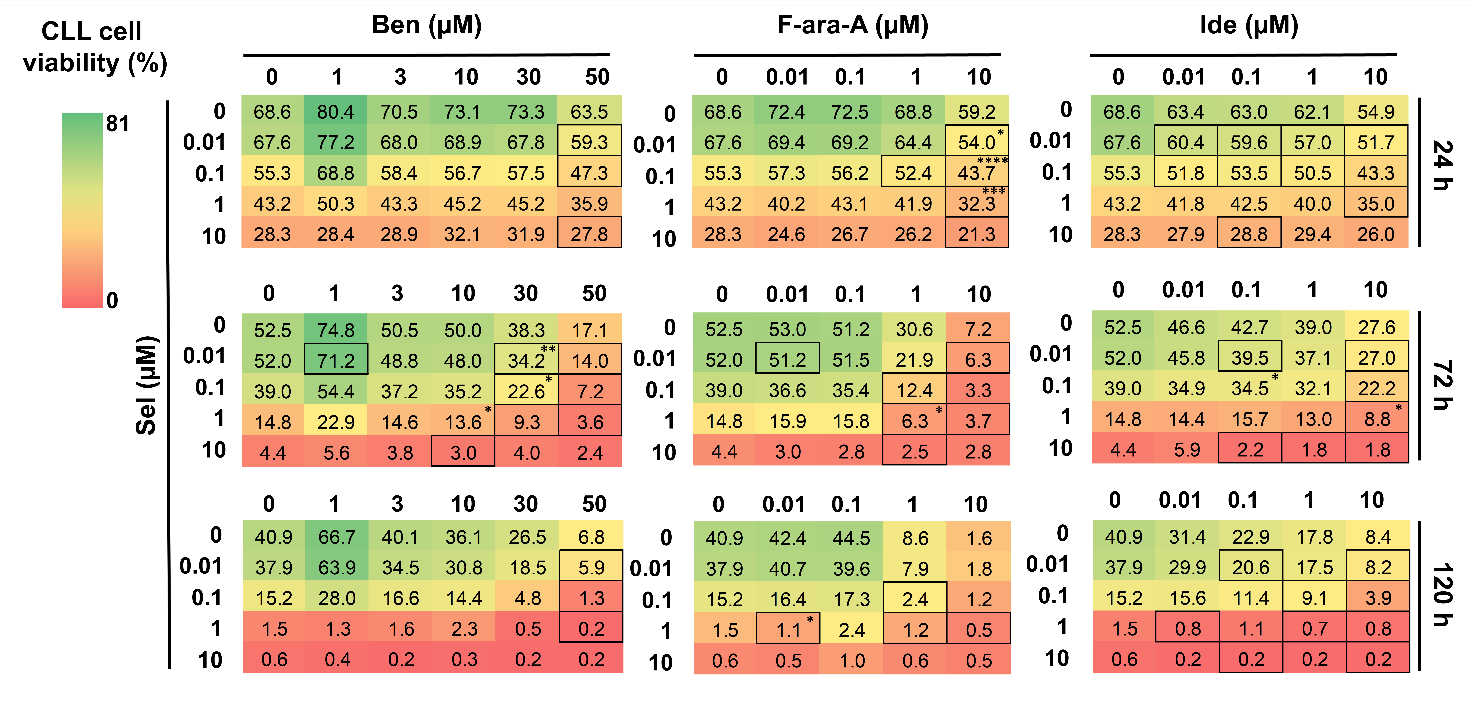


**Supplementary Figure 1. Exploratory evaluation of the cytotoxic effect of bendamustine, fludarabine and idelalisib, used in combination with selinexor, on primary CLL cells.** Heatmaps show the mean viability (% of AnnV-/PI- CLL cells) for 5 samples of patients with CLL that were co-treated *in vitro* with selinexor (Sel) + bendamustine (Ben), selinexor + fludarabine (F-ara-A) and selinexor + idelalisib (Ide) at specific drug concentrations and time points. Cells highlighted with borders indicate the combinations with a CI<1 (synergistic). Asterisks indicate the combinations which determined a significant reduction in cell viability compared to both single agents, at the corresponding concentration. **** p<0.0001, *** p<0.001, ** p<0.01 and *p<0.05.

**Supplementary Figure 2.**


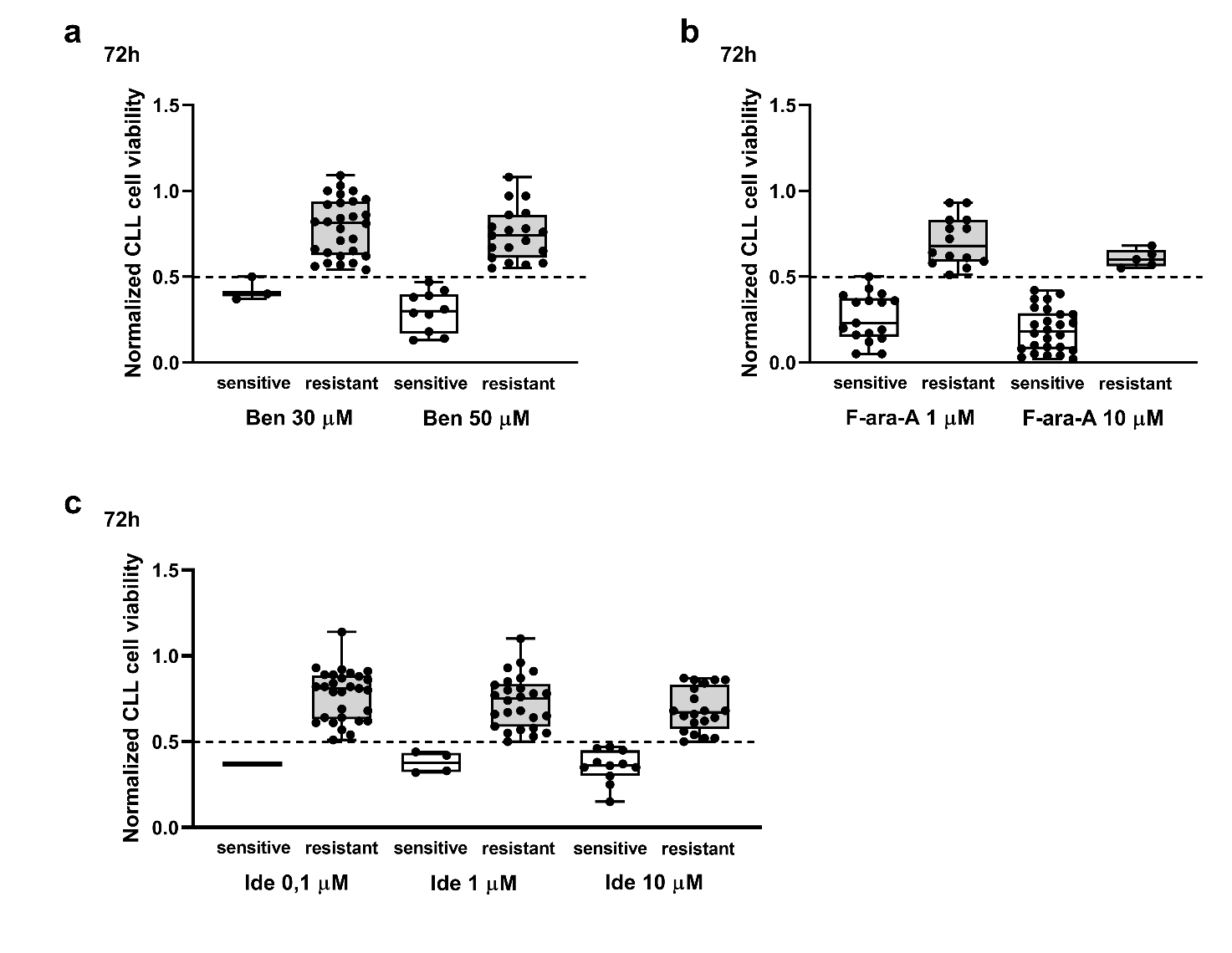


**Supplementary Figure 2. Normalized cell viability after *in vitro* treatment with bendamustine, fludarabine or idelalisib.** Normalized cell viability was calculated as the ratio between the percentage of AnnV-/PI- CLL cells cultured in the presence of bendamustine (Ben), fludarabine (F-ara-A) or idelalisib (Ide) and the percentage of AnnV-/PI- CLL cells left untreated. Samples showing a 72-hour normalized cell viability after single-agent-treatment ≤ 0.5 were defined “sensitive”, otherwise sample were defined “resistant”. Box plots represent median values and 25%-75% percentiles, whiskers represent minimum and maximum values for each group; each point represents a single sample.

**Supplementary Figure 3.**


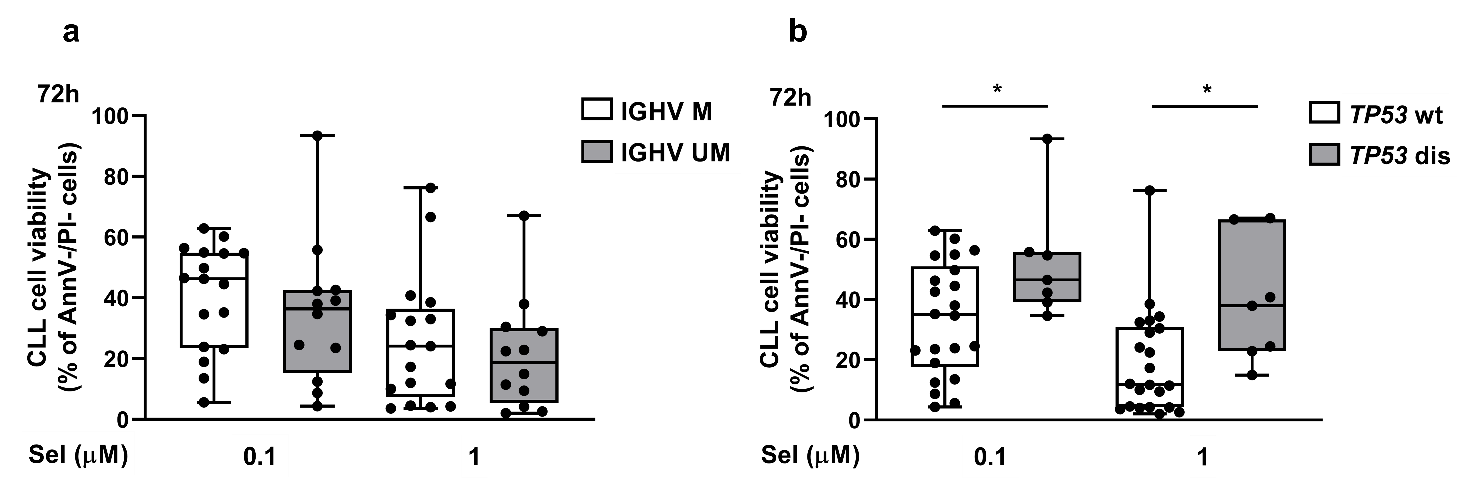


**Supplementary Figure 3. Cytotoxic effect of single-agent selinexor in specific molecular subgroups of CLL samples.** Patients were grouped based on IGHV mutational status (a) or *TP53* status (patients with del(17p) and/or *TP53* mutations were considered *TP53*-disrupted, *TP53*^dis^, whereas patients without del(17p) and/or *TP53* mutations were considered *TP53* wild type, *TP53*^wt^) (b), and cell viability was assessed after 72-hour culture with single-agent selinexor (Sel). Box plots represent median value and 25%-75% percentiles, whiskers represent minimum and maximum values for each group; each point represents a single sample. * p<0.05.

**Supplementary Figure 4.**


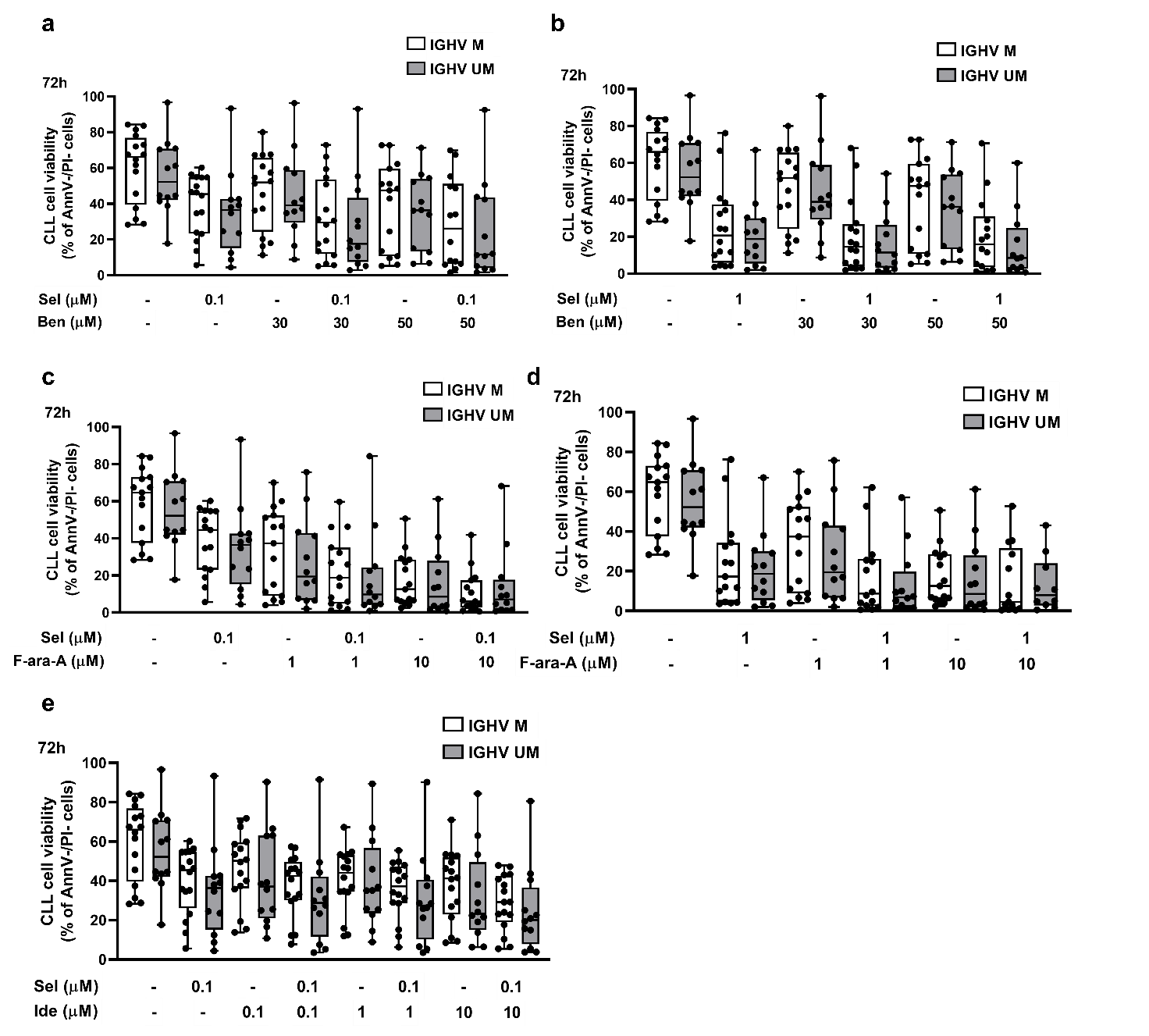


**Supplementary Figure 4. Selinexor-based combinations have similar effects on viability of CLL cells from IGHV mutated and unmutated patients.** Patients were grouped based on IGHV mutational status. Cell viability was assessed after 72-hour culture with Selinexor (Sel) + bendamustine (Ben), fludarabine (F-ara-A) or idelalisib (Ide). Box plots represent median value and 25%-75% percentiles, whiskers represent minimum and maximum values for each group; each point represents a single sample. together with all points.

**Supplementary Figure 5.**


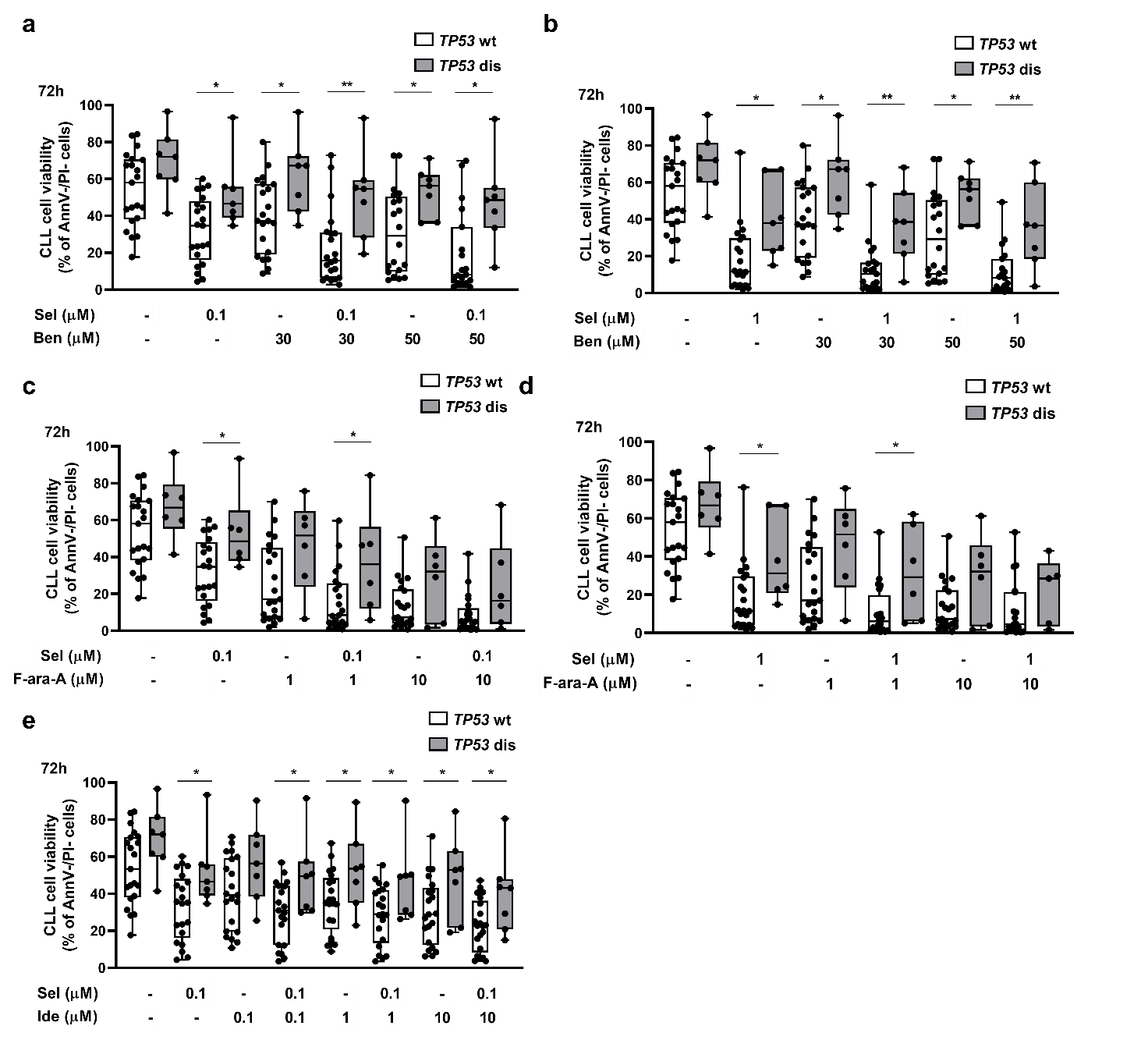


**Supplementary Figure 5. Selinexor-based combinations differently affect viability of CLL cells from *TP53* wild type and *TP53* disrupted patients.** Patients were grouped based *TP53* status (patients with del(17p) and/or *TP53* mutations were considered *TP53*-disrupted, *TP53*^dis^, whereas patients without del(17p) nor *TP53* mutations were considered *TP53* wild type, *TP53*^wt^), and normalized cell viability was assessed after 72-hour culture with single-agent selinexor (Sel). Box plots represent median value and 25%-75% percentiles, whiskers represent minimum and maximum values for each group; each point represents a single sample. ** p<0.01, * p<0.05.

**Supplementary Figure 6.**


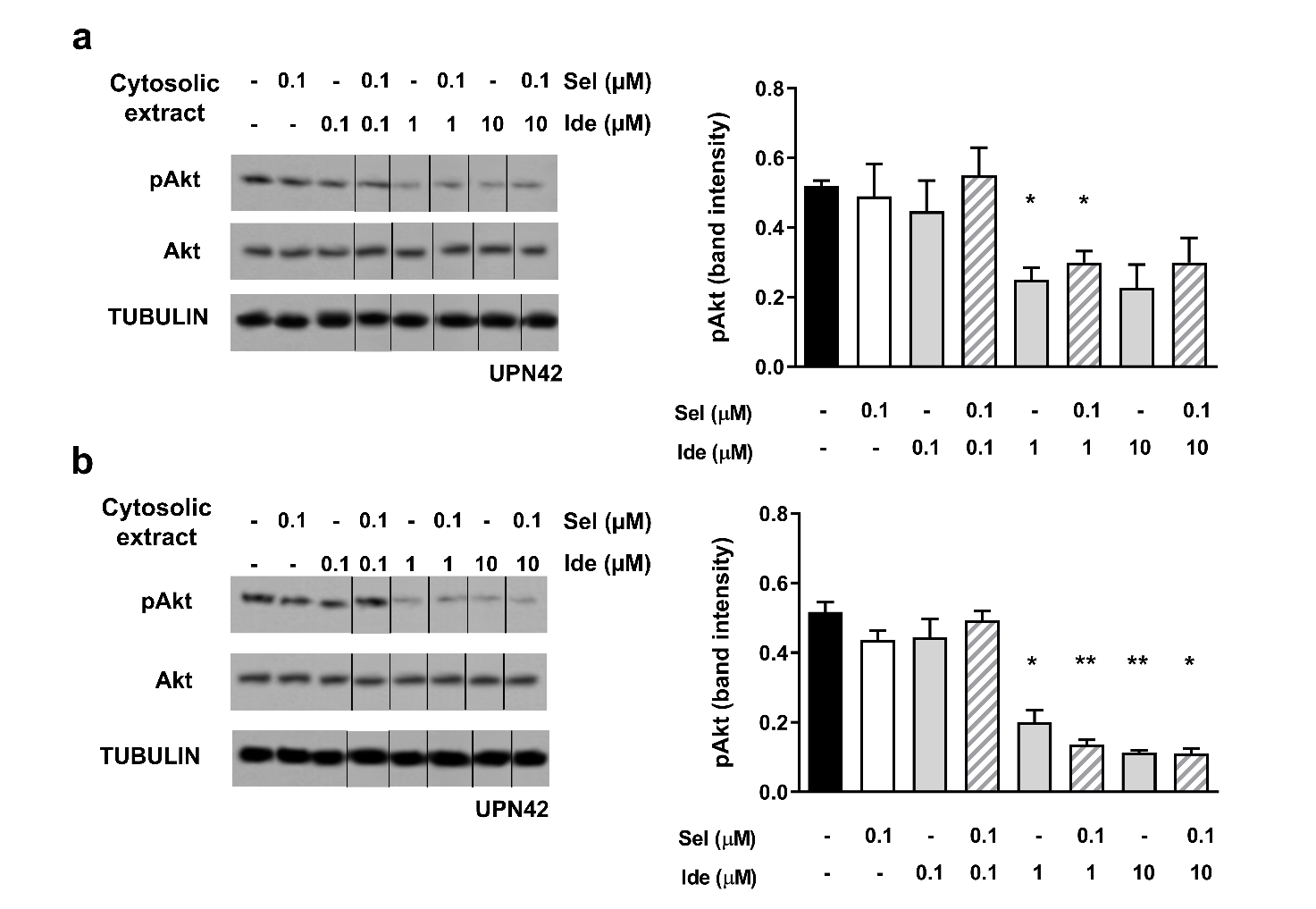


**Supplementary Figure 6. The combination of selinexor and idelalisib exerts Akt inhibition at different time points and concentrations.** Primary CLL cells were exposed to selinexor (Sel) and idelalisib (Ide) at specified concentrations, and Akt phosphorylation was evaluated after 6 hours (a) and 12 hours (b). In (a) and (b) a representative blot (with relative Unique Patient Number, UPN42), together with the corresponding cumulative band intensity data of 3 independent experiments, respectively, is shown. Repositioned gel lanes are indicated by vertical lines. Bar graphs represent mean results obtained from three experiments together with standard error of mean. Asterisks indicate the experimental conditions which determined a significant reduction in Akt phosphorylation compared to untreated control ** p<0.01, * p<0.05.

**Supplementary Figure 7.**


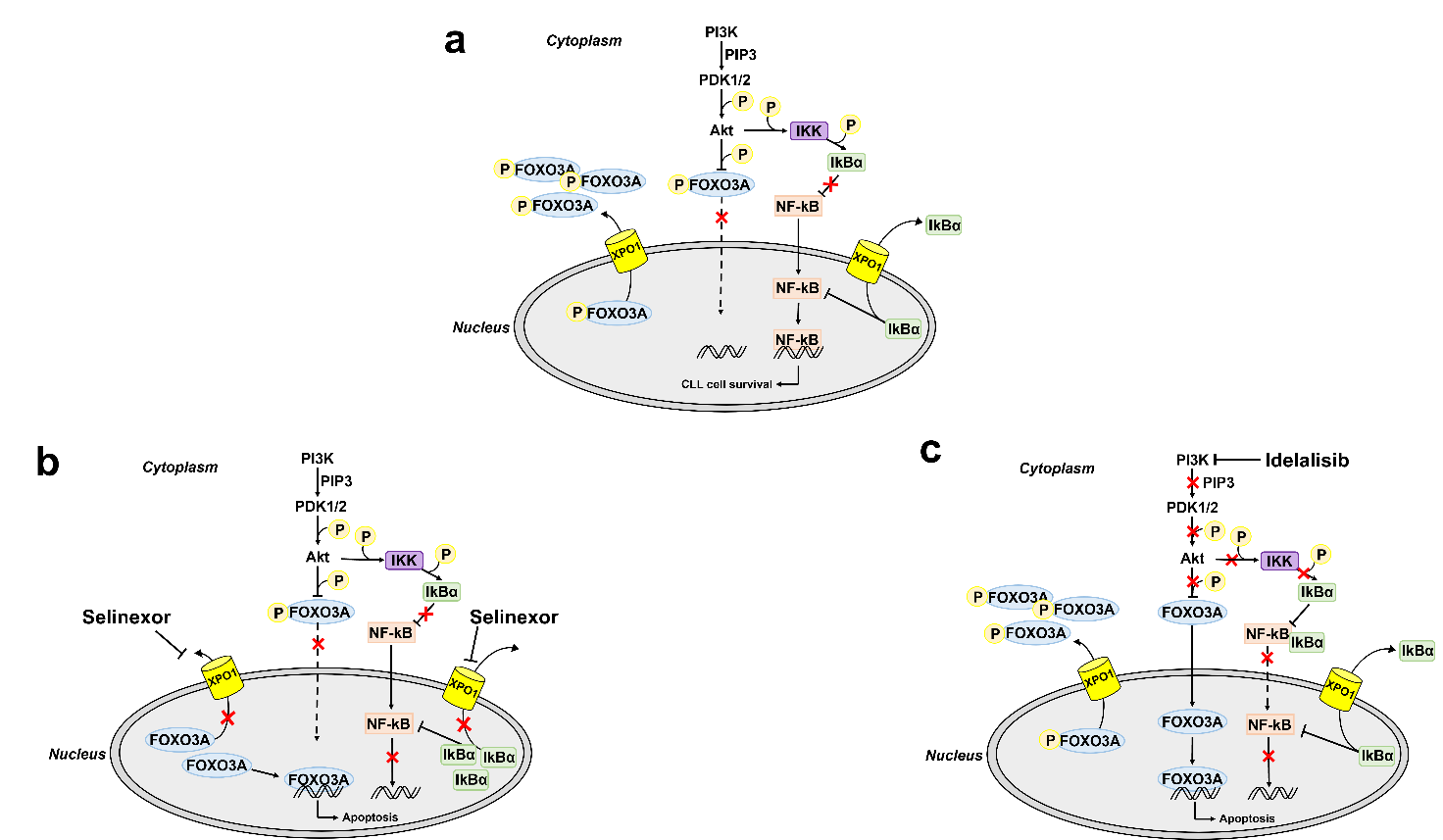


**Supplementary Figure 7. Schematic representation of the role of XPO1, XPO1 inhibition, PI3K pathway activity and PI3K inhibition on the cellular localization of the NF-kB inhibitor IkBα and of the tumor suppressor FOXO3A.** (a) IkBα and FOXO3A are among the proteins carried by XPO1. The activity of XPO1 favors the export from the nucleus to the cytoplasm of the NF-kB inhibitor IkBα, thus promoting the pro-survival activities of NF-kB. FOXO3A is a transcription factor with tumor suppressor activities. Akt - by phosphorylating FOXO3A - leads to its cytoplasmic sequestration and degradation, thus reducing its nuclear accumulation and transcriptional activity. (b) Selinexor blocks XPO1 thus favoring the nuclear retention of the tumor suppressor FOXO3A and of the NF-kB inhibitor IkBα. (c) Idelalisib inhibits the PI3K/Akt signalling pathway, resulting in a reduced phosphorylation of IkBα, which thus retains its inhibitory effect on NF-kB, and of FOXO3A that is then sequestered in the cytosol and degraded.

**Supplementary Figure 8.**


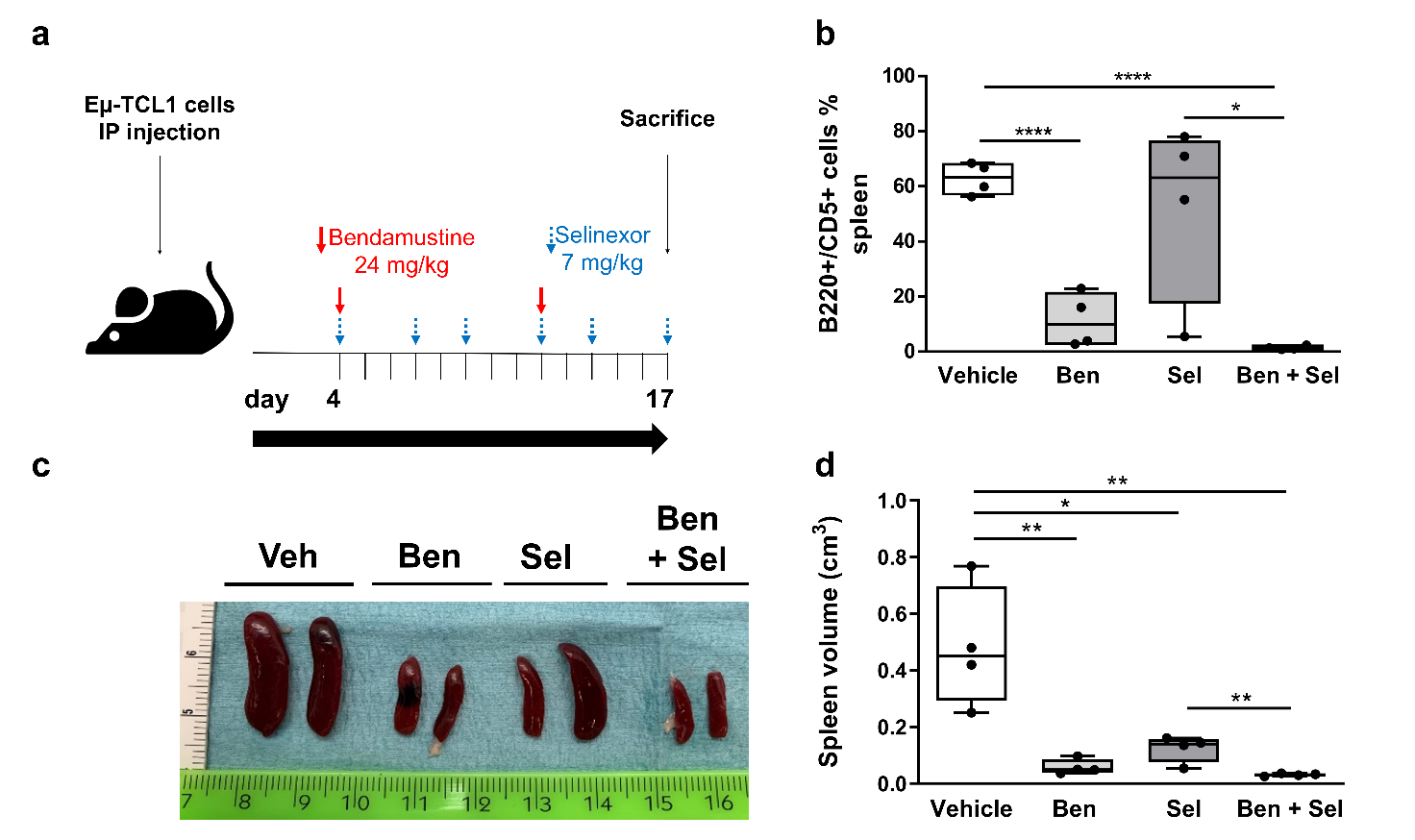


**Supplementary Figure 8. The selinexor + bendamustina combination is effective in reducing the tumor burden *in vivo*.** Mice transplanted with the Eµ-TCL1-derived leukemia were randomly treated with selinexor and bendamustine, used as single agents or in combination, or with a vehicle. A schematic outline of *in vivo* experiments is depicted in panel a. Panel b shows the percentage of leukemic cells (CD5+/B220+ cells) in the spleen of mice transplanted with the Eµ-TCL1-derived leukemia after 2 weeks of treatment. Panel c shows the macroscopic view of the spleens from two representative animals for each treatment group, and panel d shows the spleen volume for all treated animals. In panels b and d box and whiskers plots represent median values, 25–75% percentiles, and minimum and maximum values for each group; each point represents a single sample. **** p<0.0001, ** p<0.01 and * p<0.05.

**Supplementary Figure 9.**


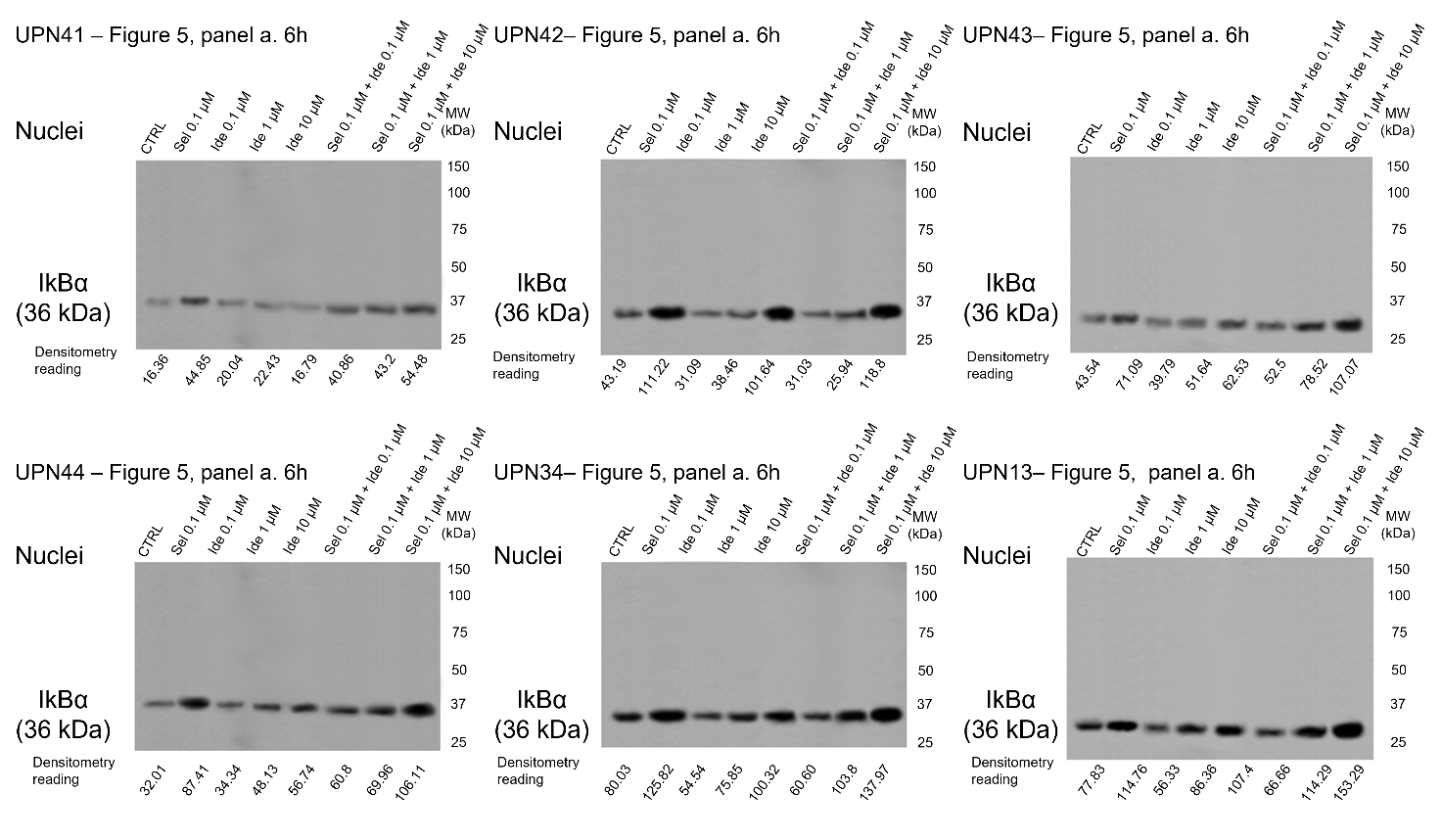


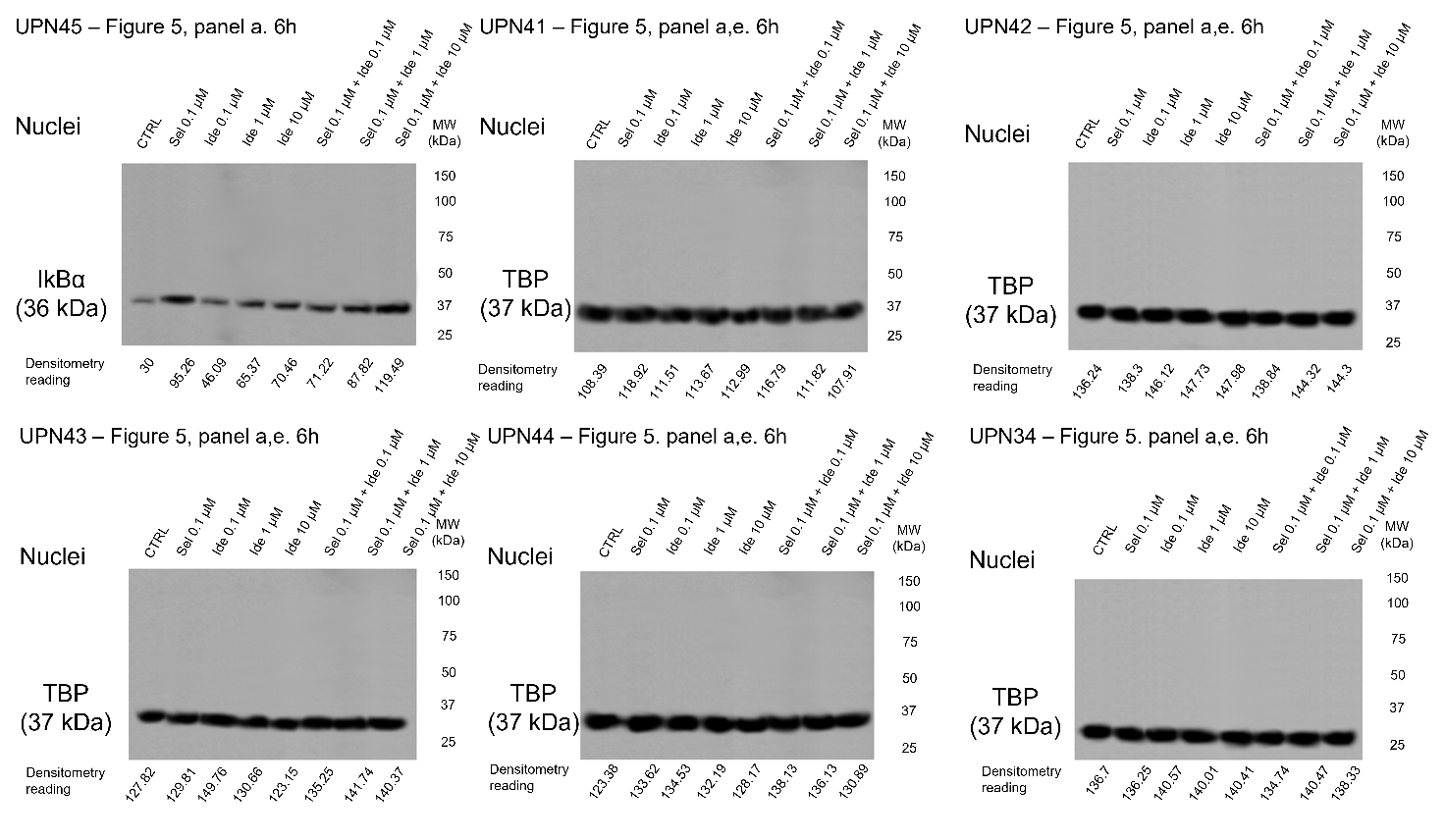


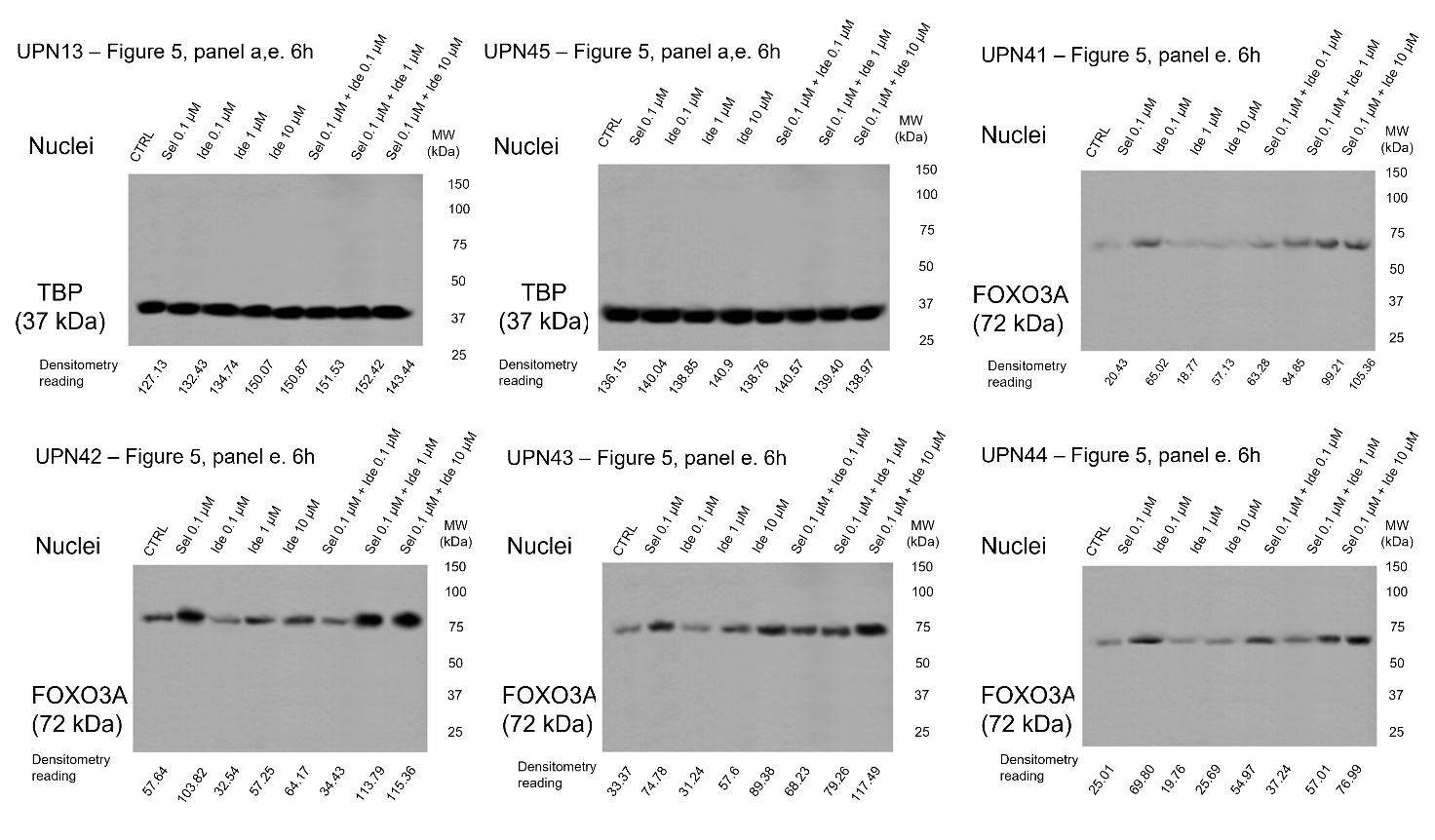

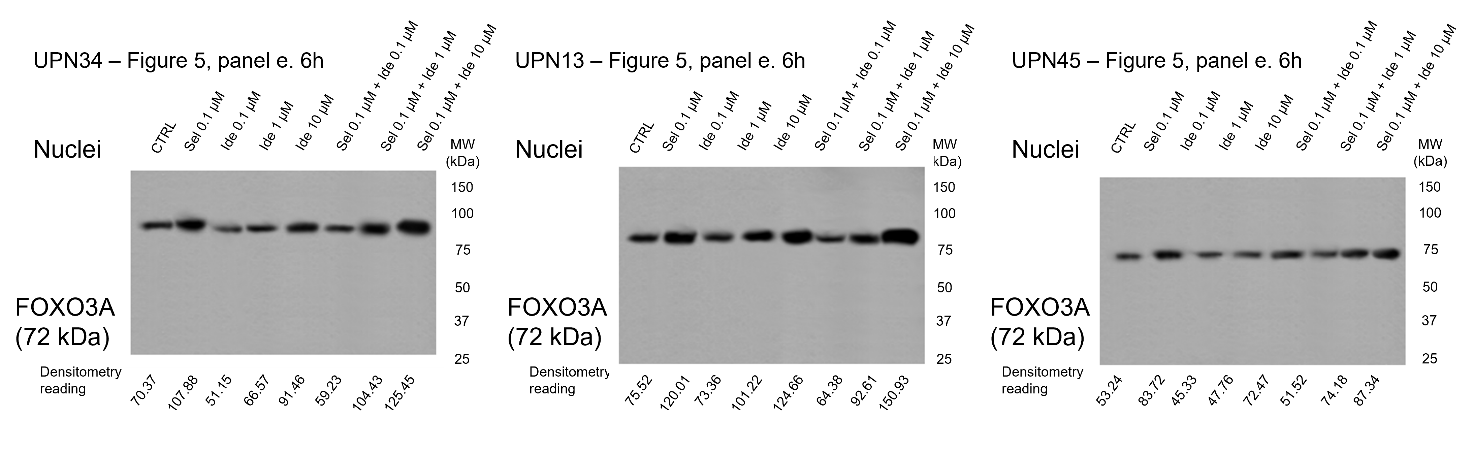

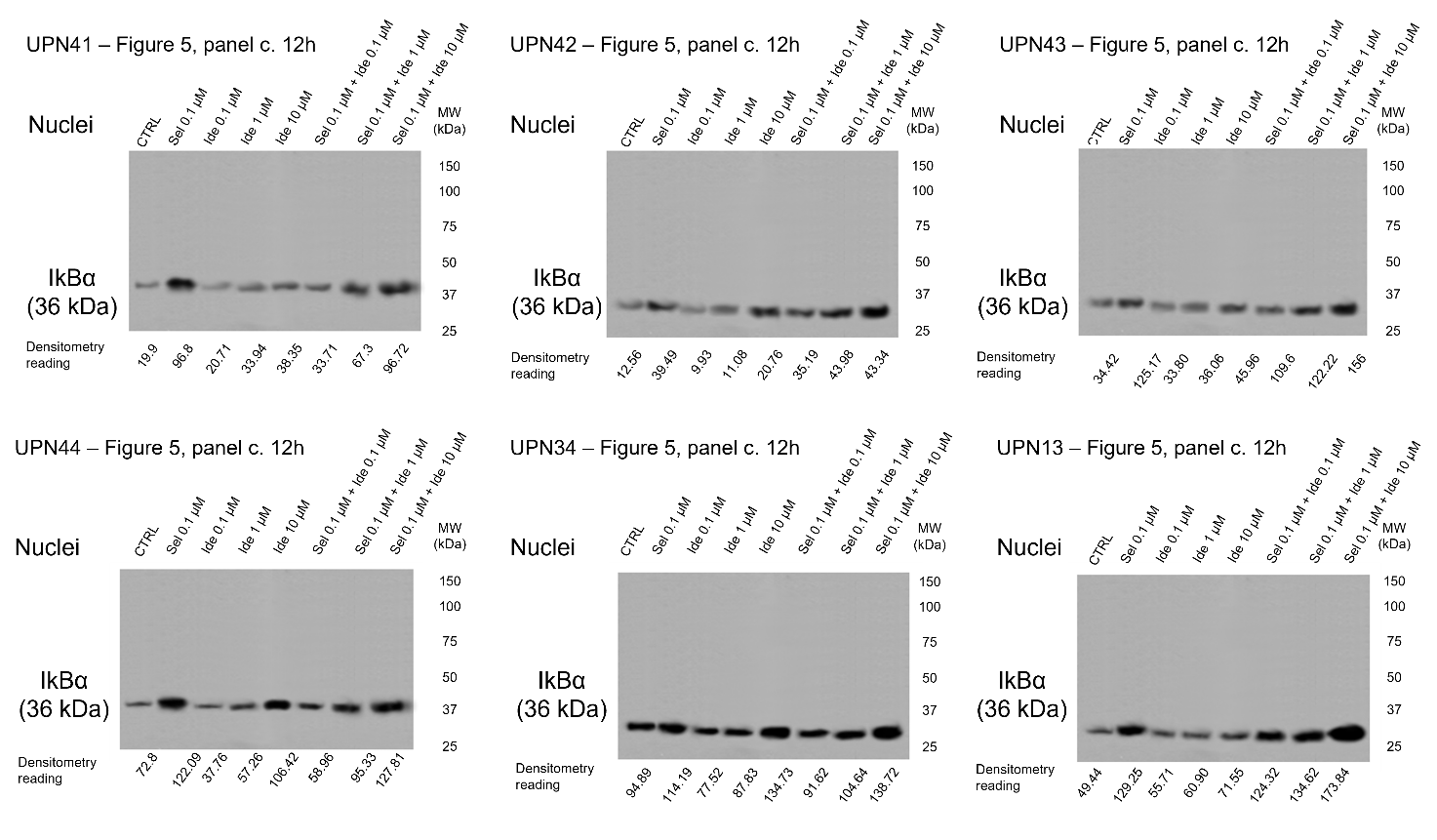

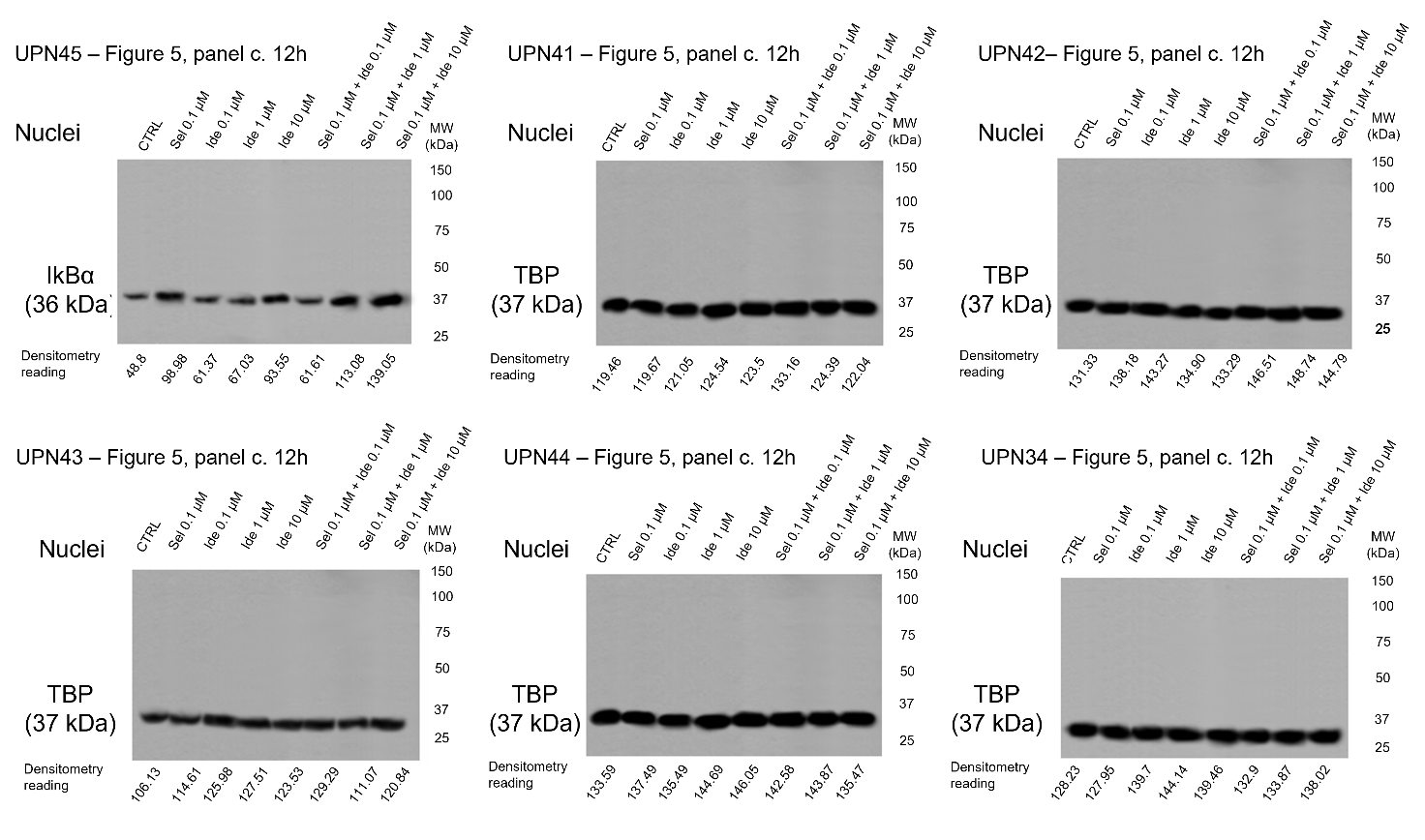

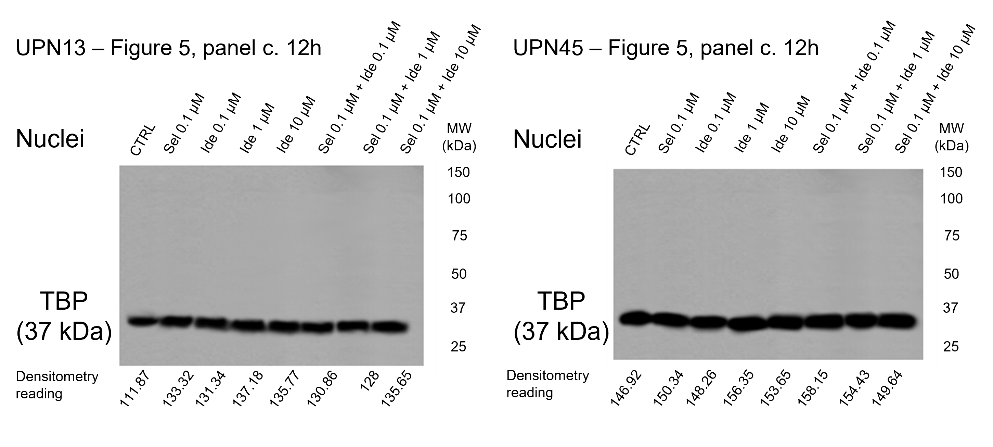

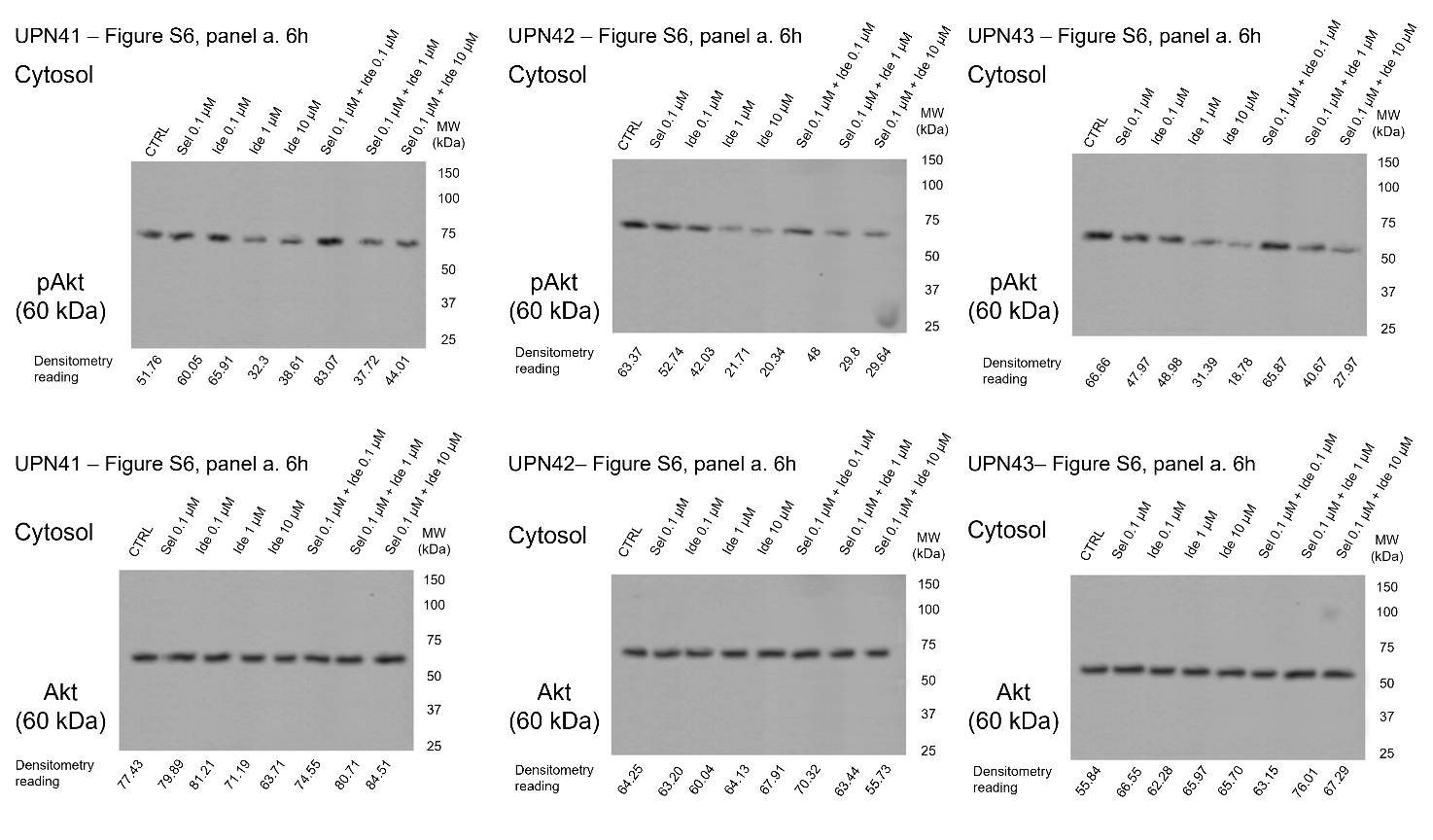

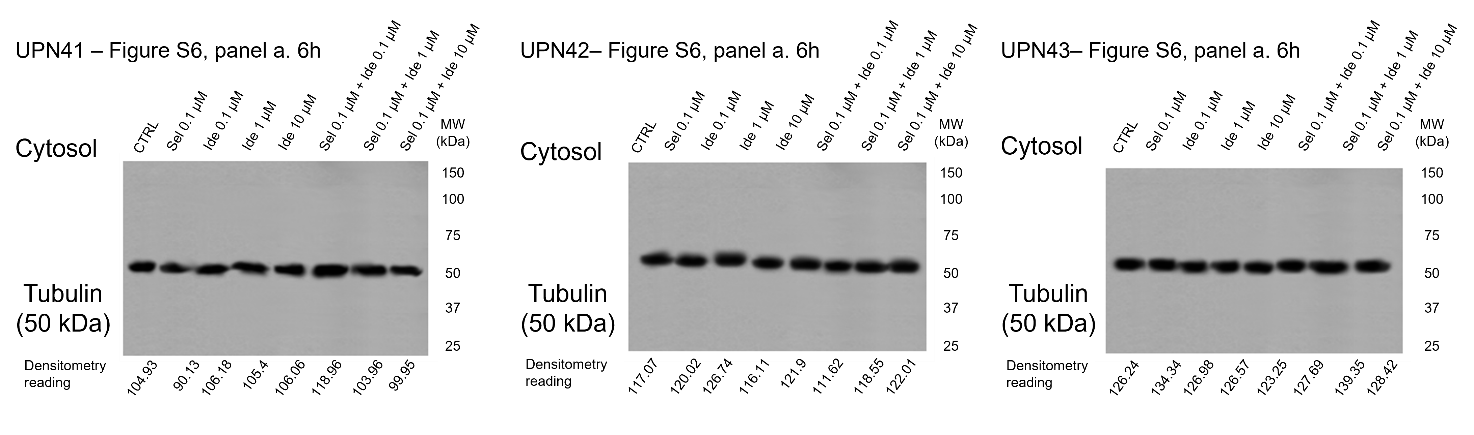

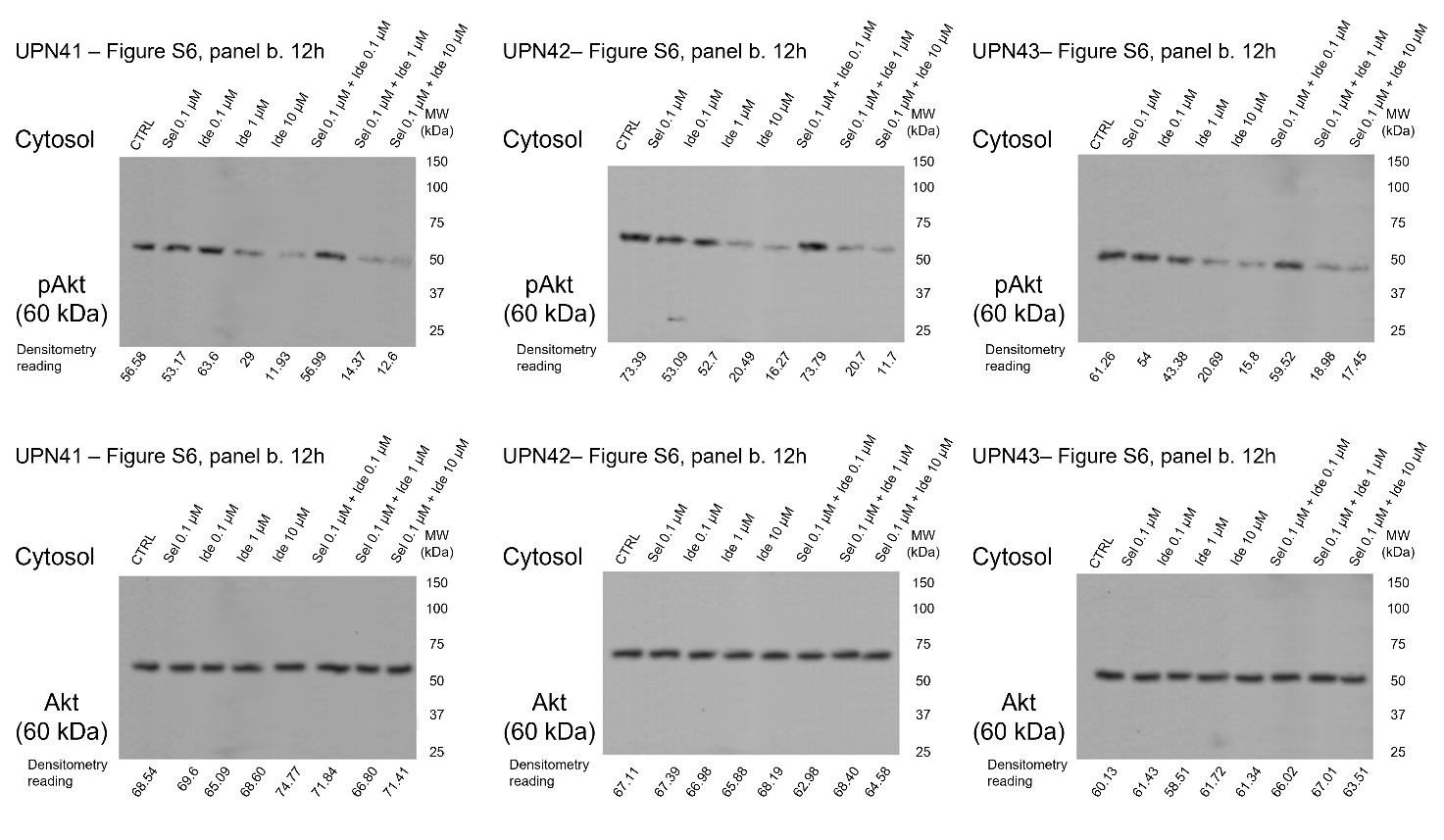

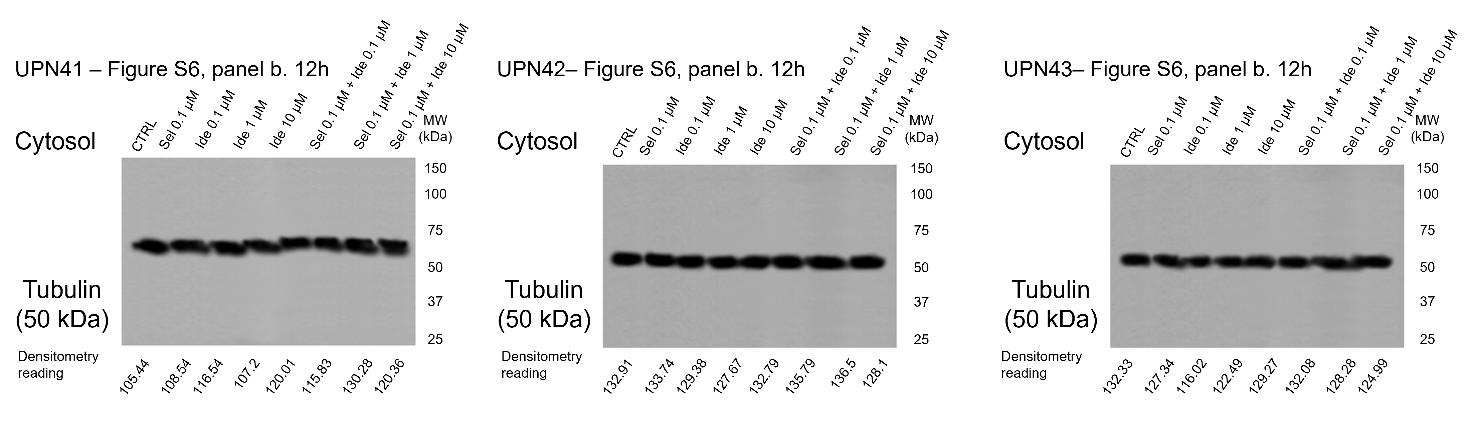


**Supplementary Figure 9. Original whole western blots**

Primary CLL cells were exposed to selinexor (Sel) and idelalisib (Ide) at specified concentrations. IkBα and TBP nuclear amount was evaluated after 6 and 12 hours. FOXO3A nuclear amount was evaluated after 6 hours. Original whole western blots together with densitometry reading of 7 independent experiments (Unique Patient Number, UPN41, UNP42, UPN43, UPN44, UPN34, UPN13 and UPN45) are shown. Data were included in Figure 5 (panels a, c, and e). pAkt, Akt and tubulin cytosolic amount was evaluated after 6 and 12 hours. Original whole western blots together with densitometry reading of 3 independent experiments (Unique Patient Number, UPN41, UNP42 and UPN43) are reported. Data were included in Figure S6 (panels a and b).

**Supplementary Figure 10.**


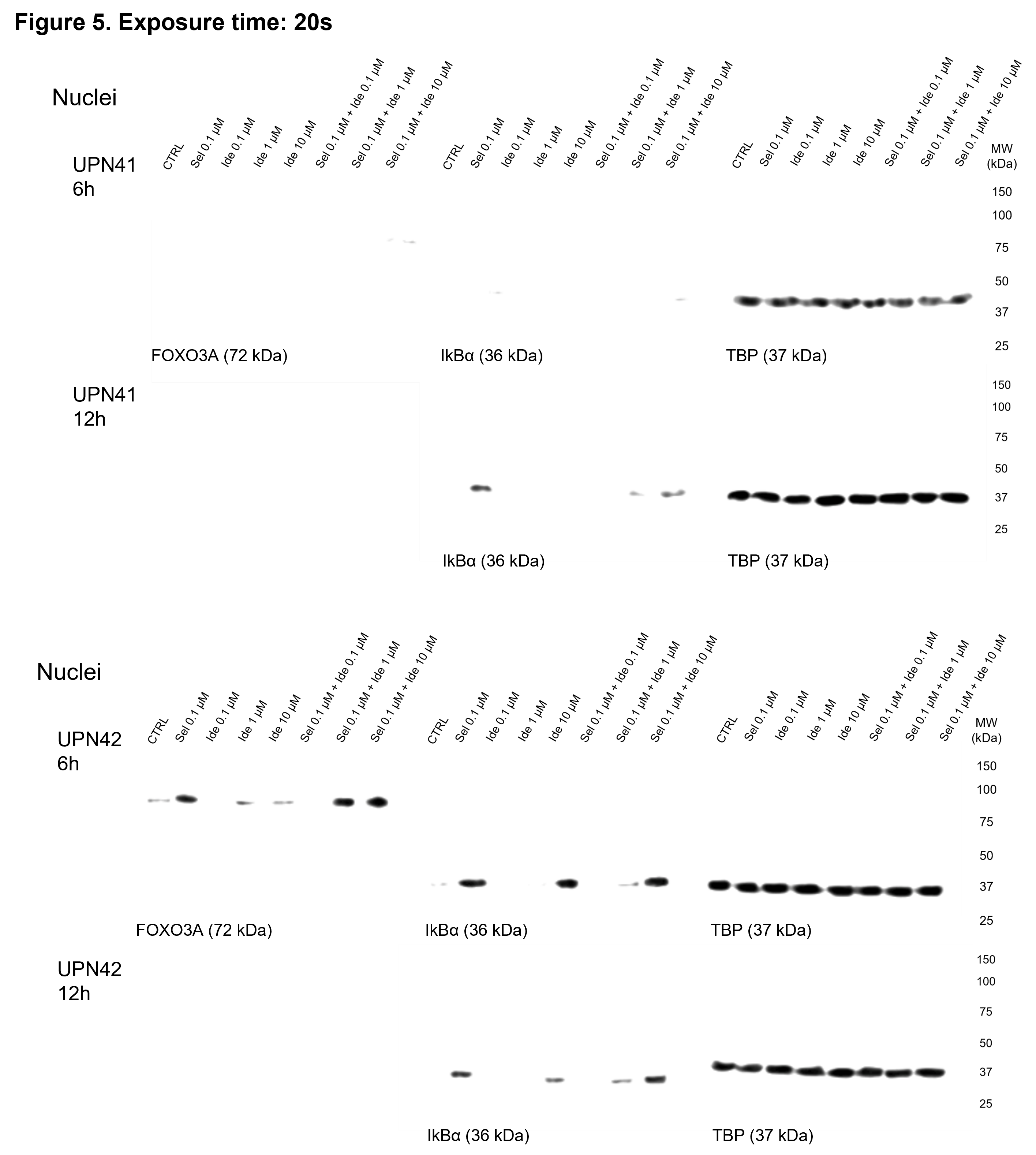

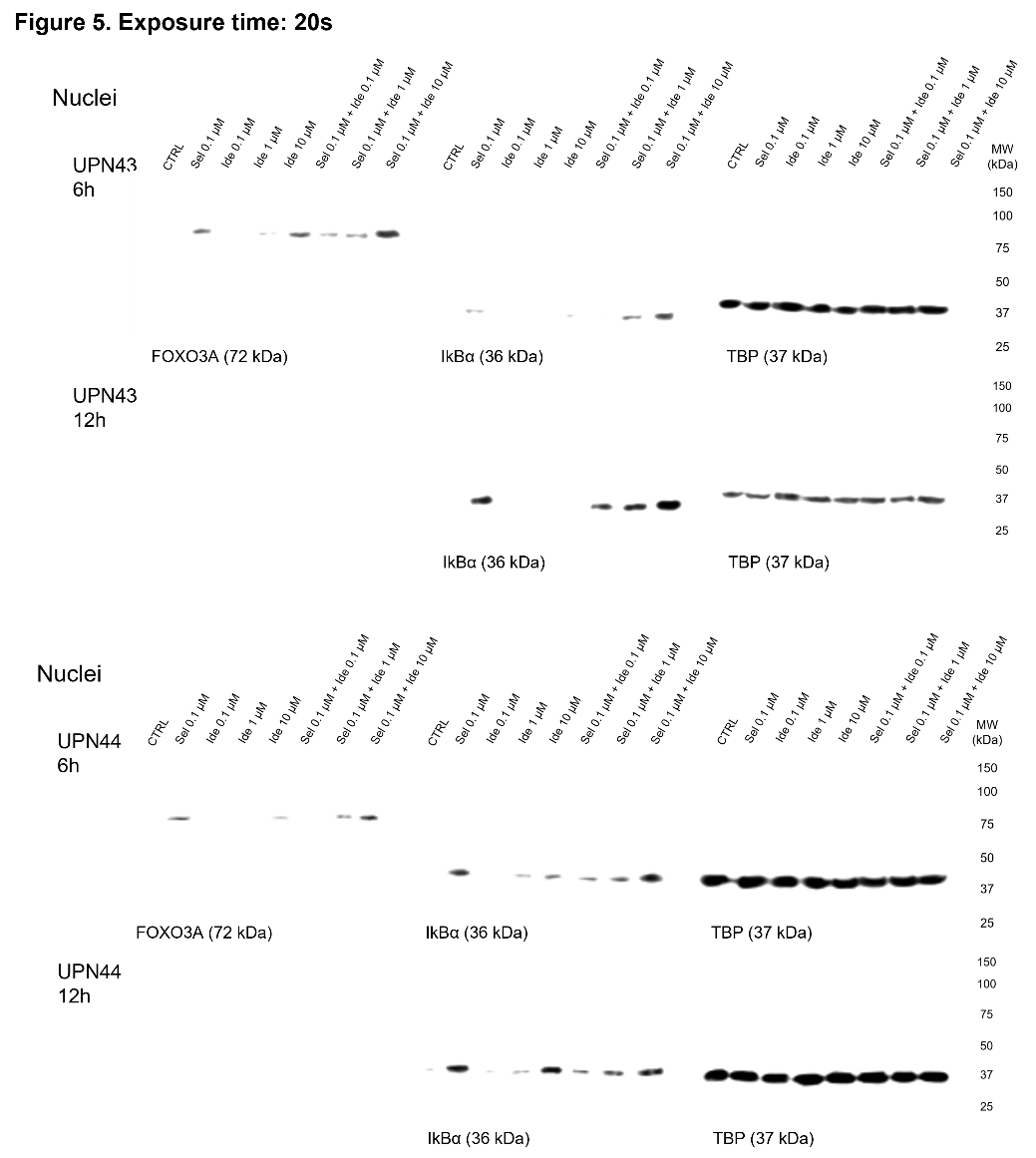


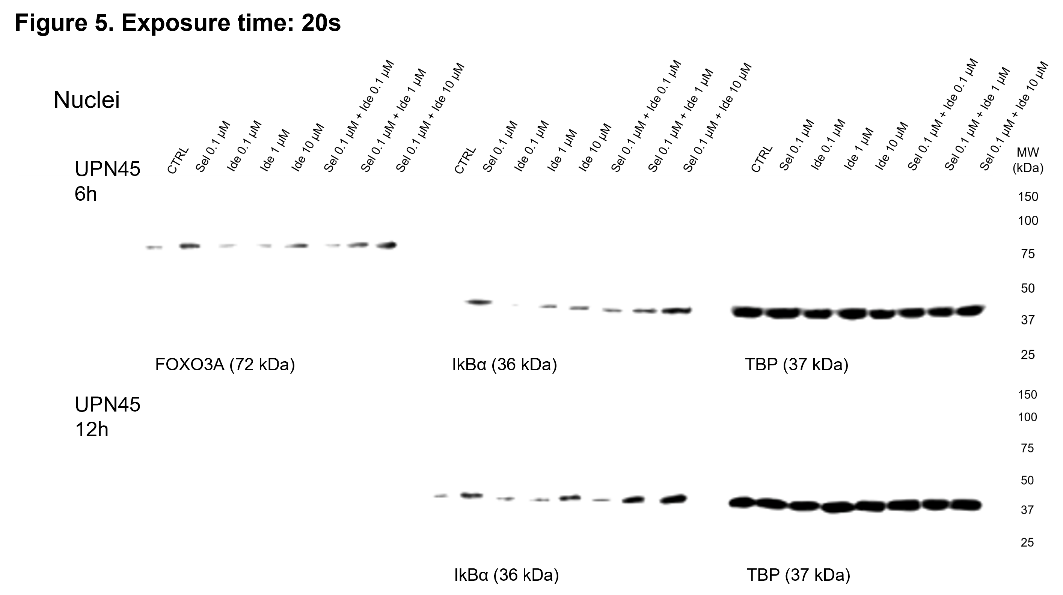


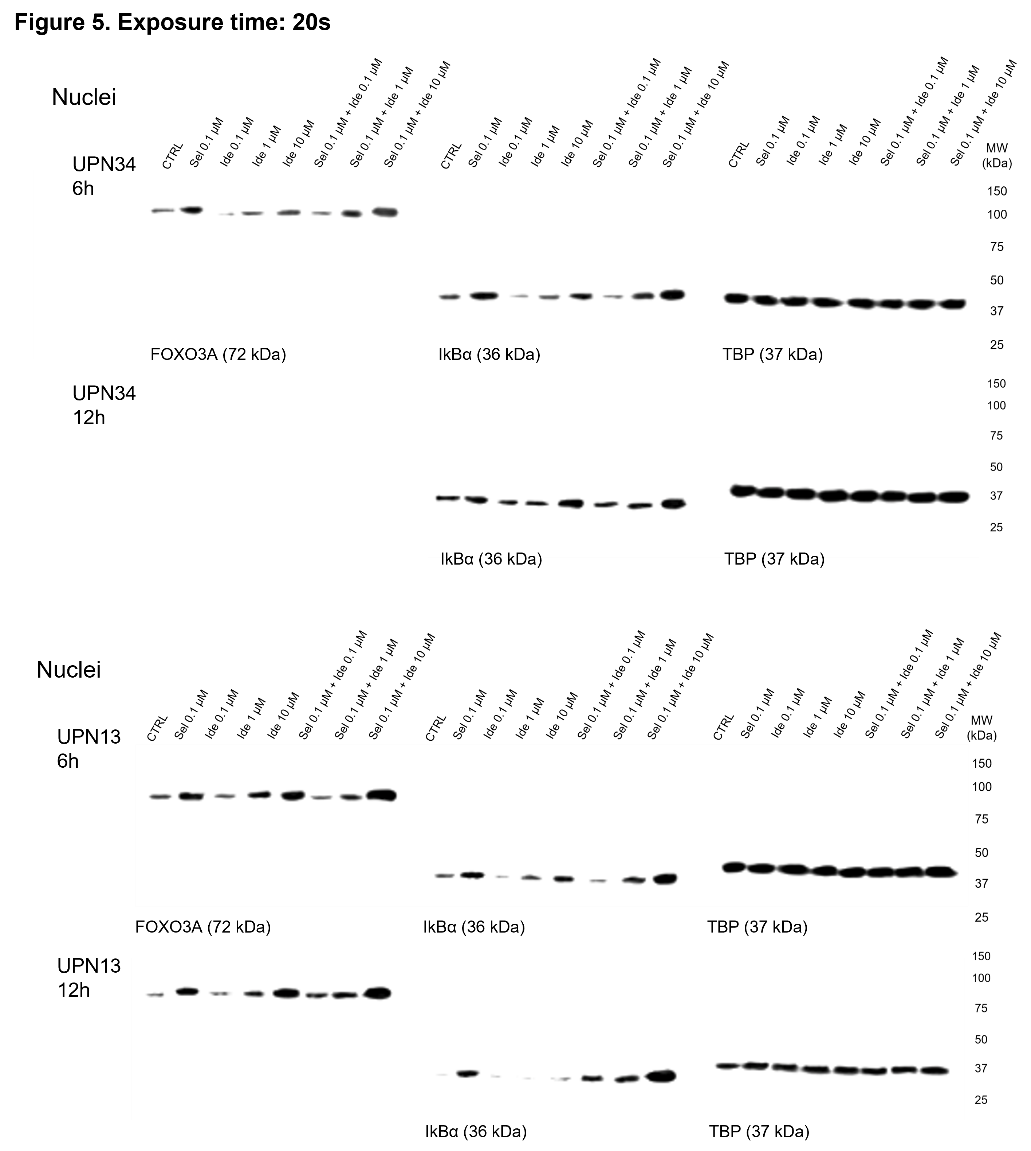


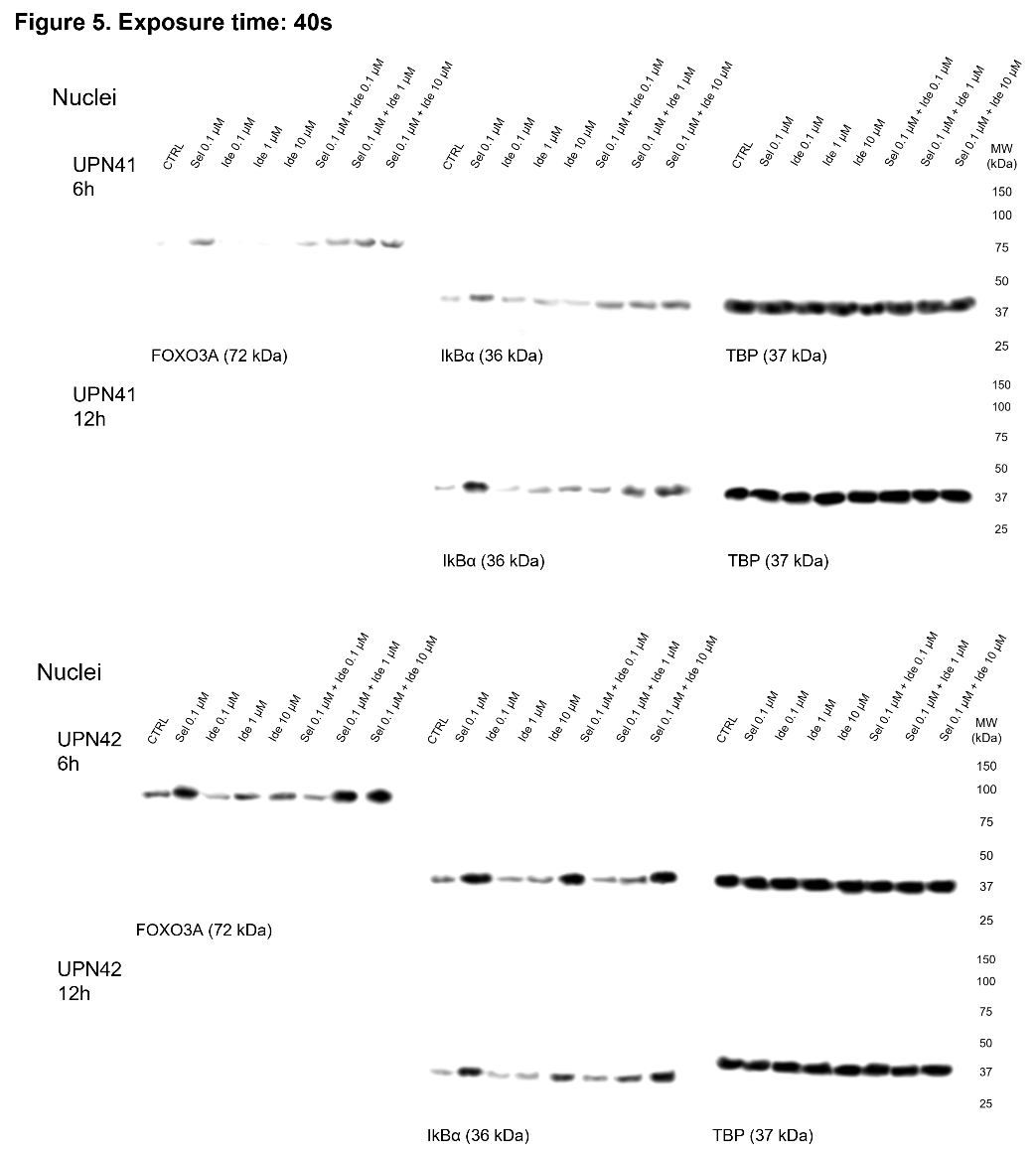

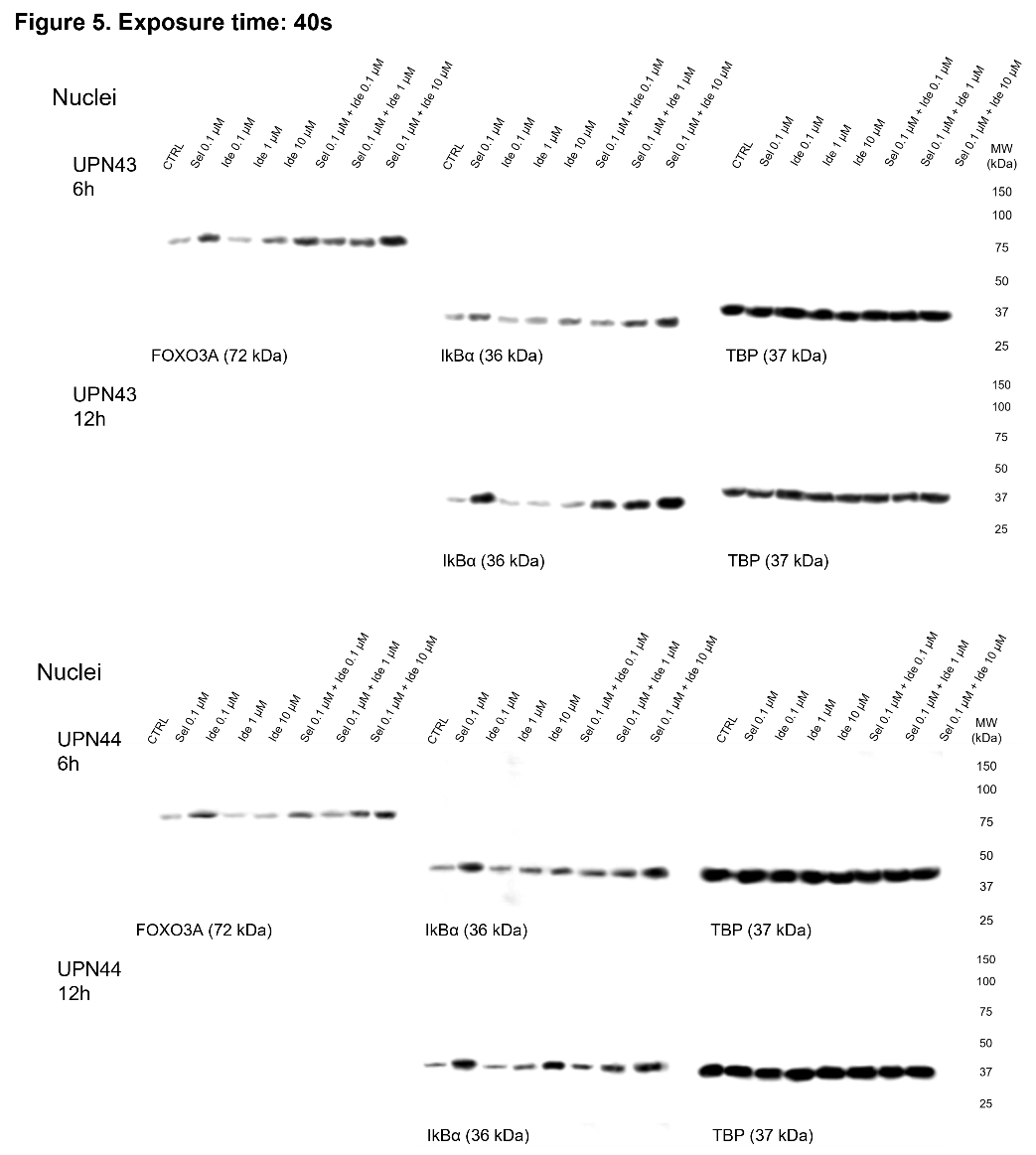


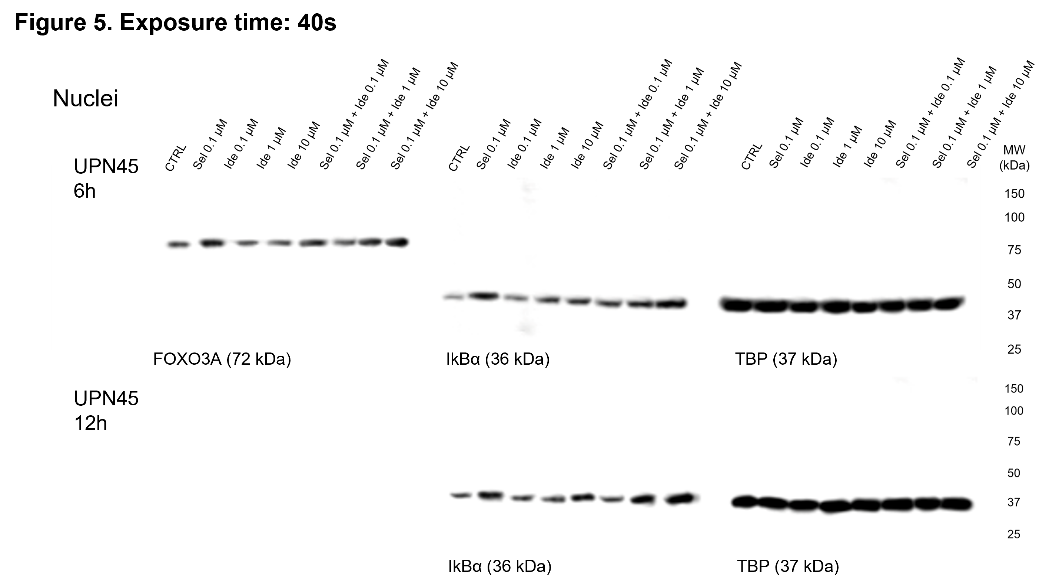

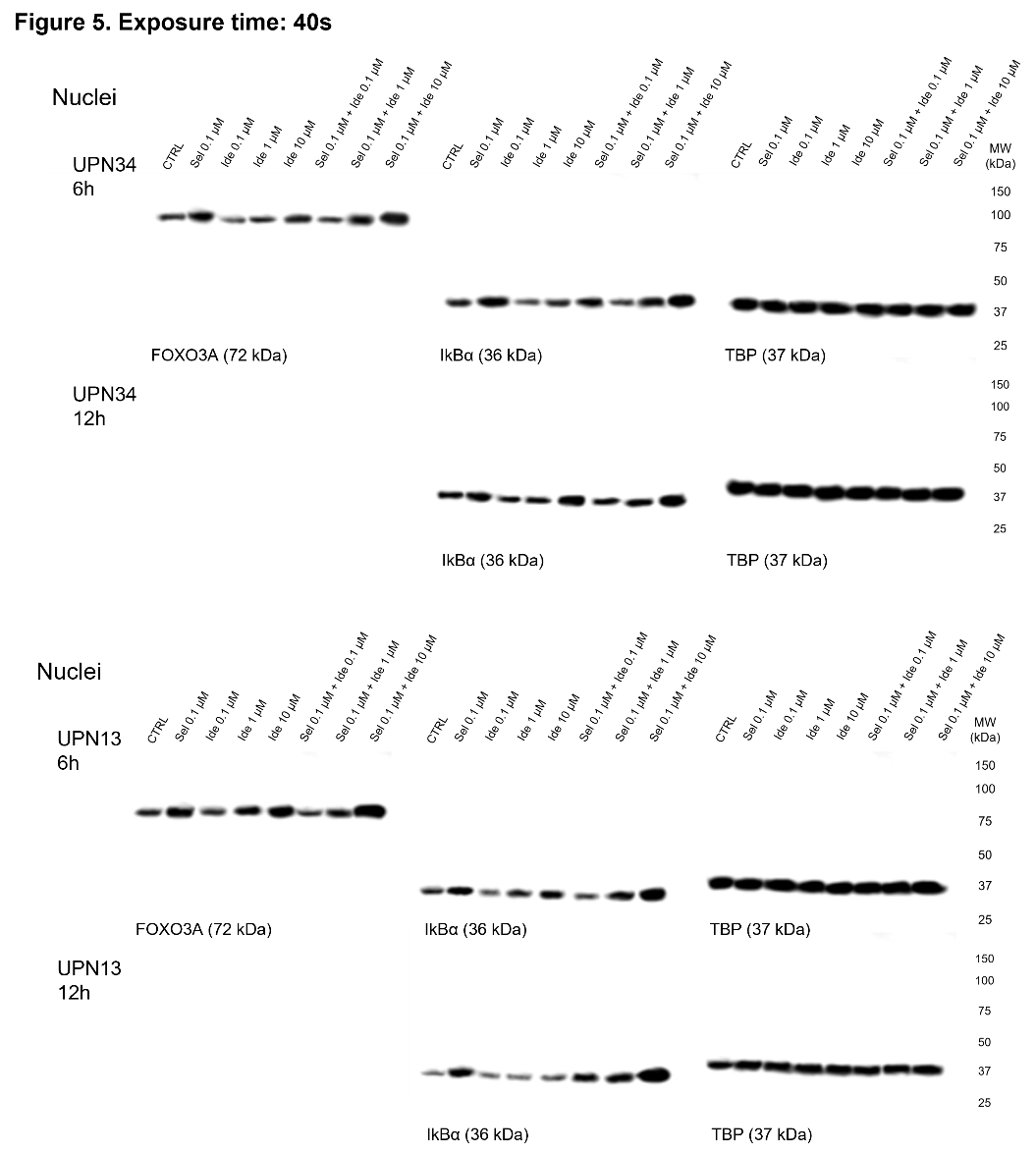


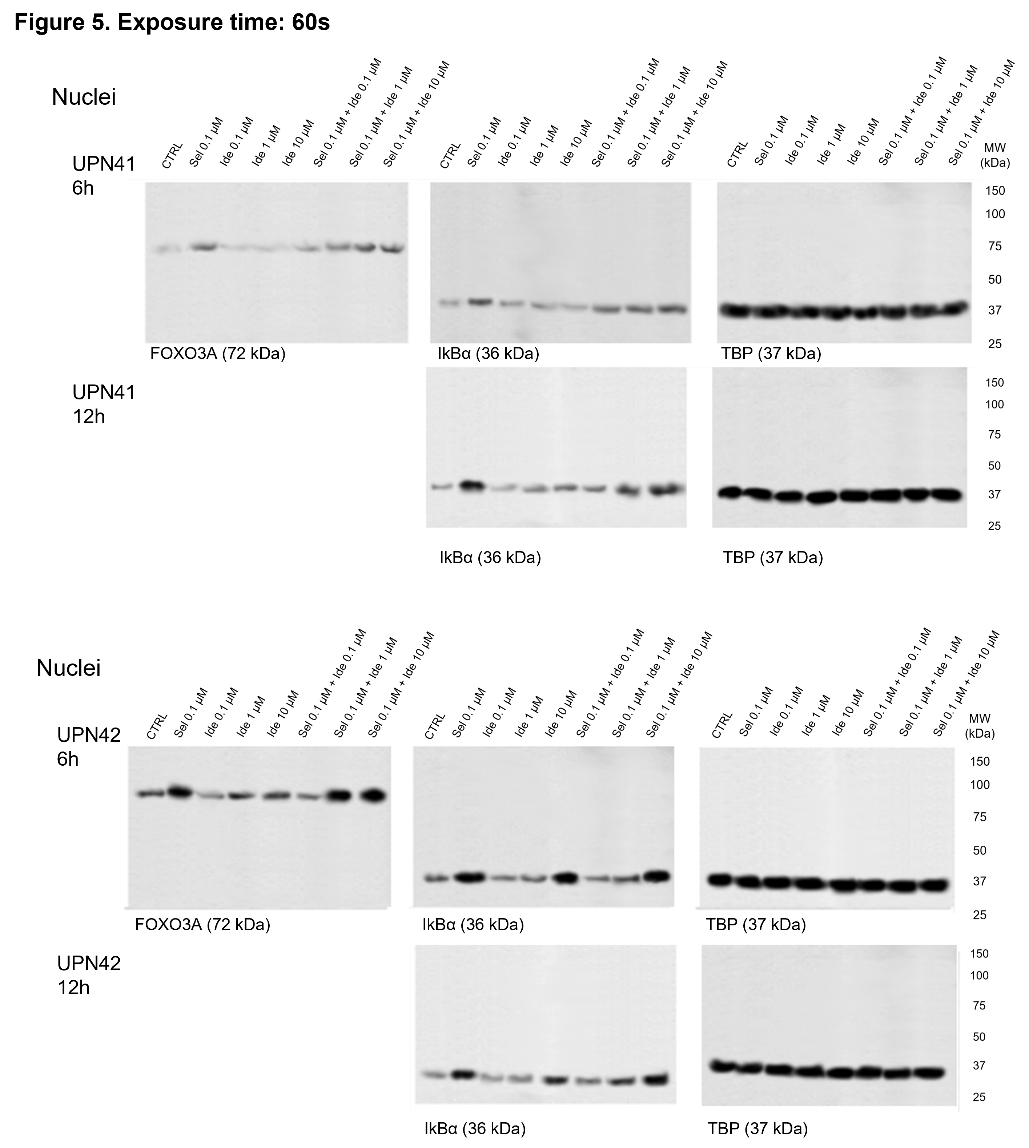

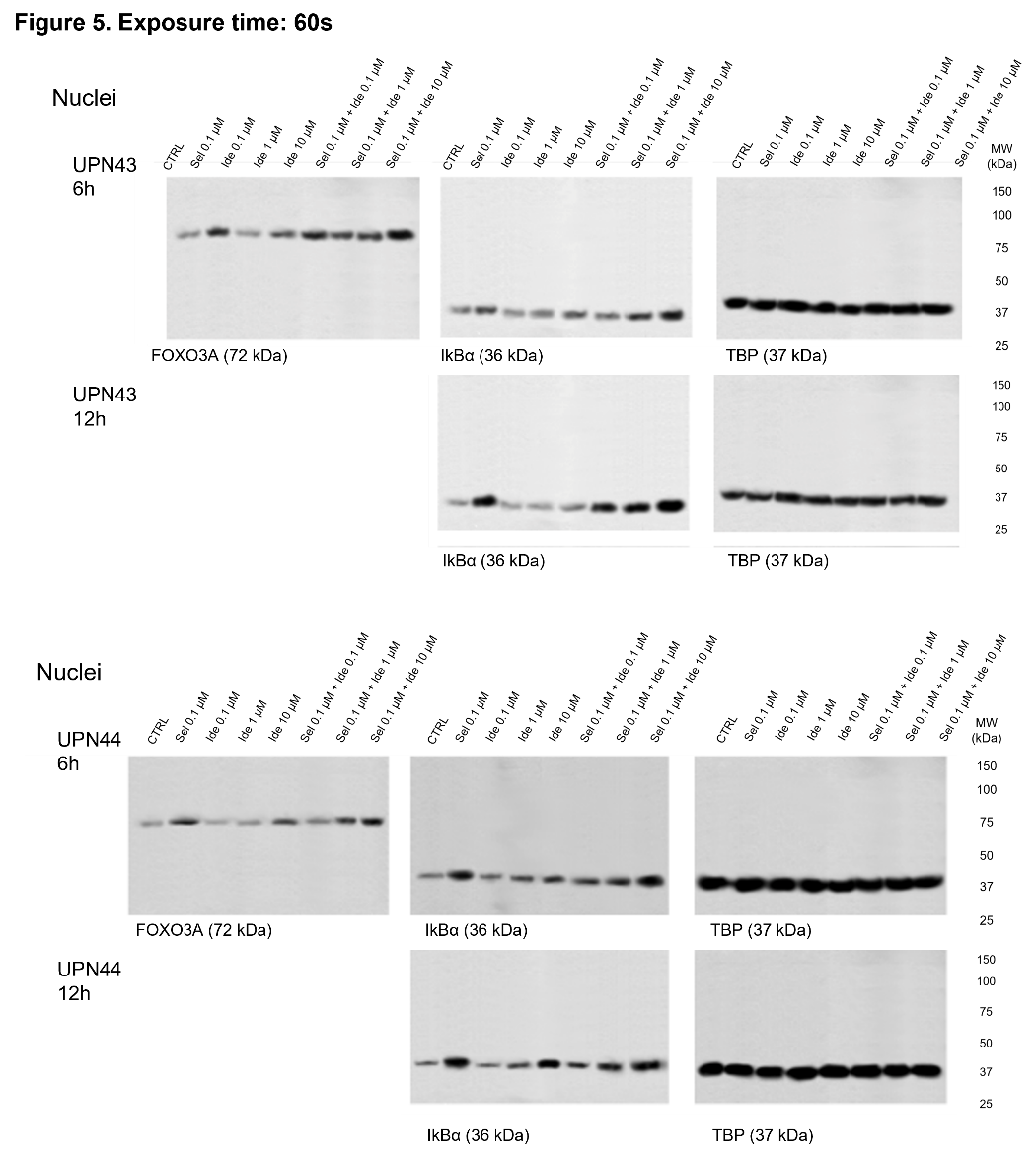


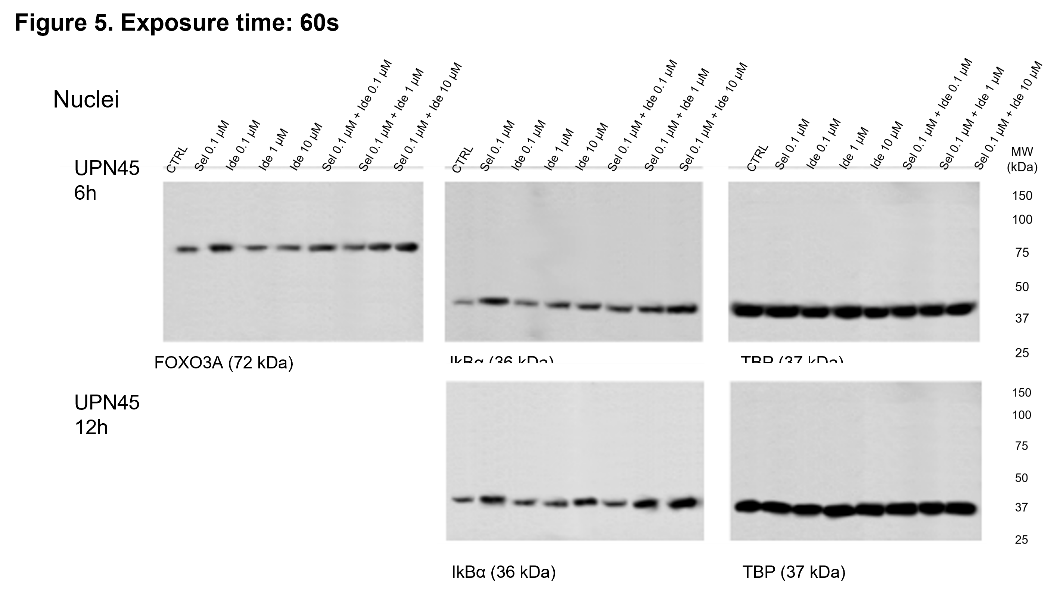

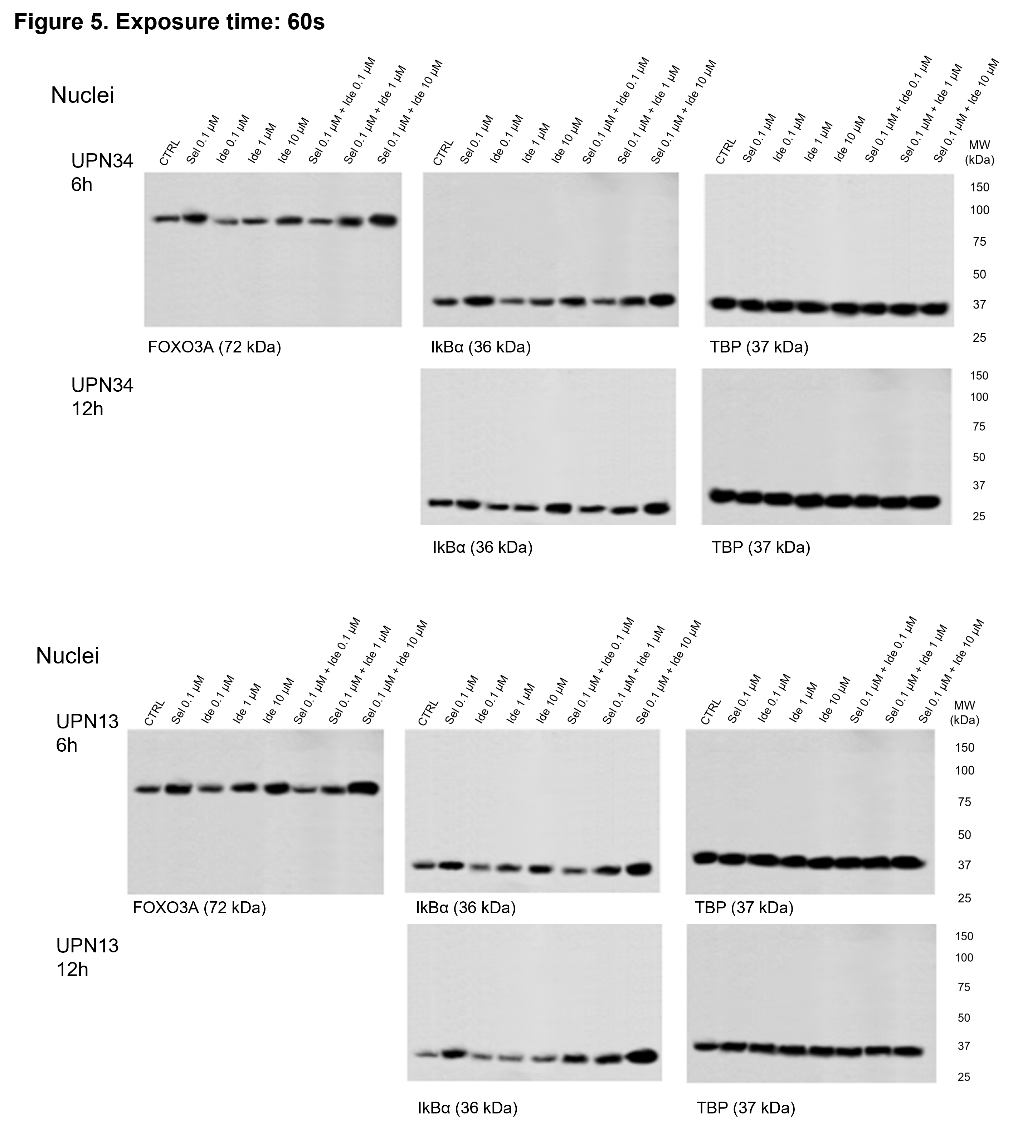


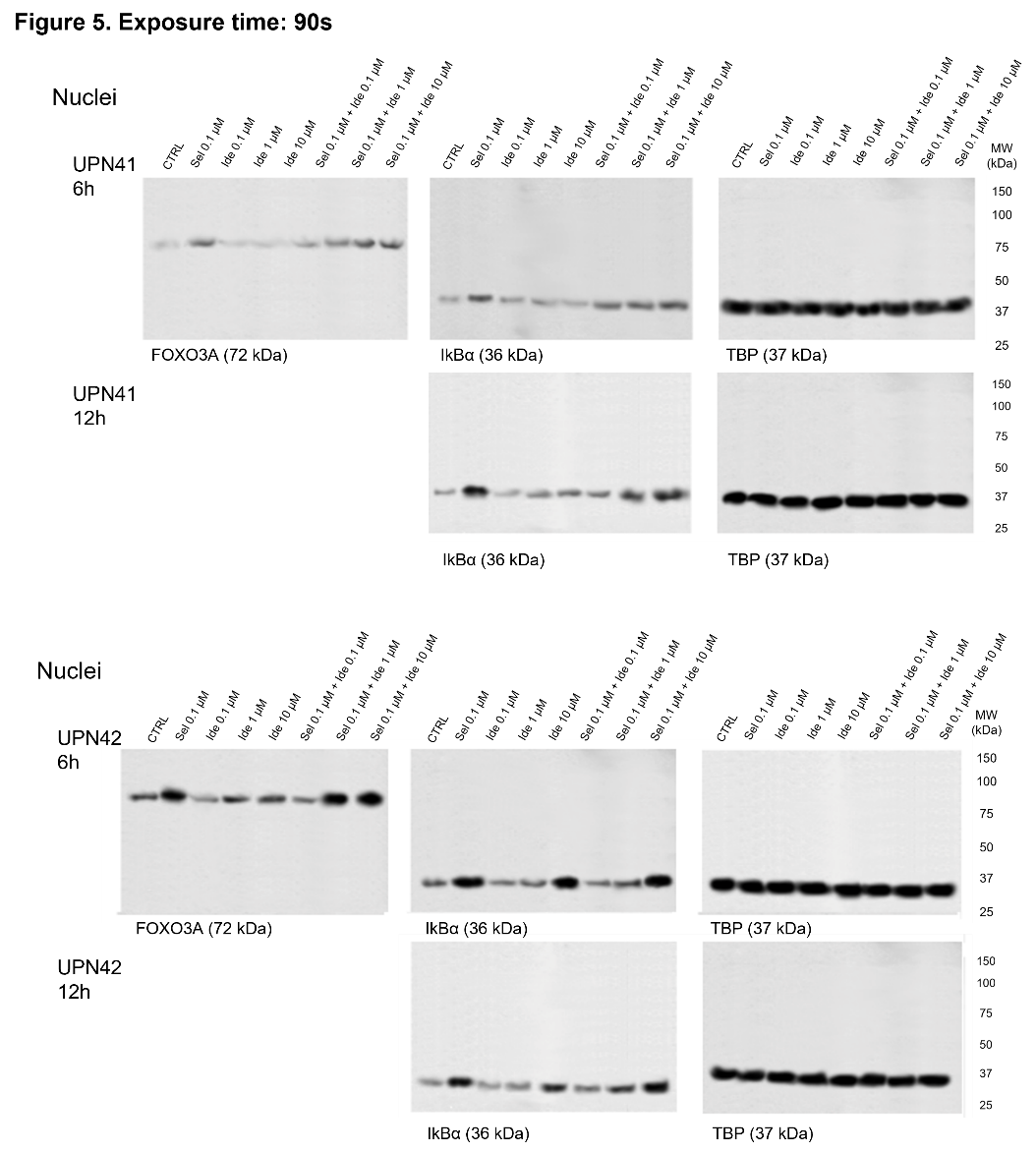

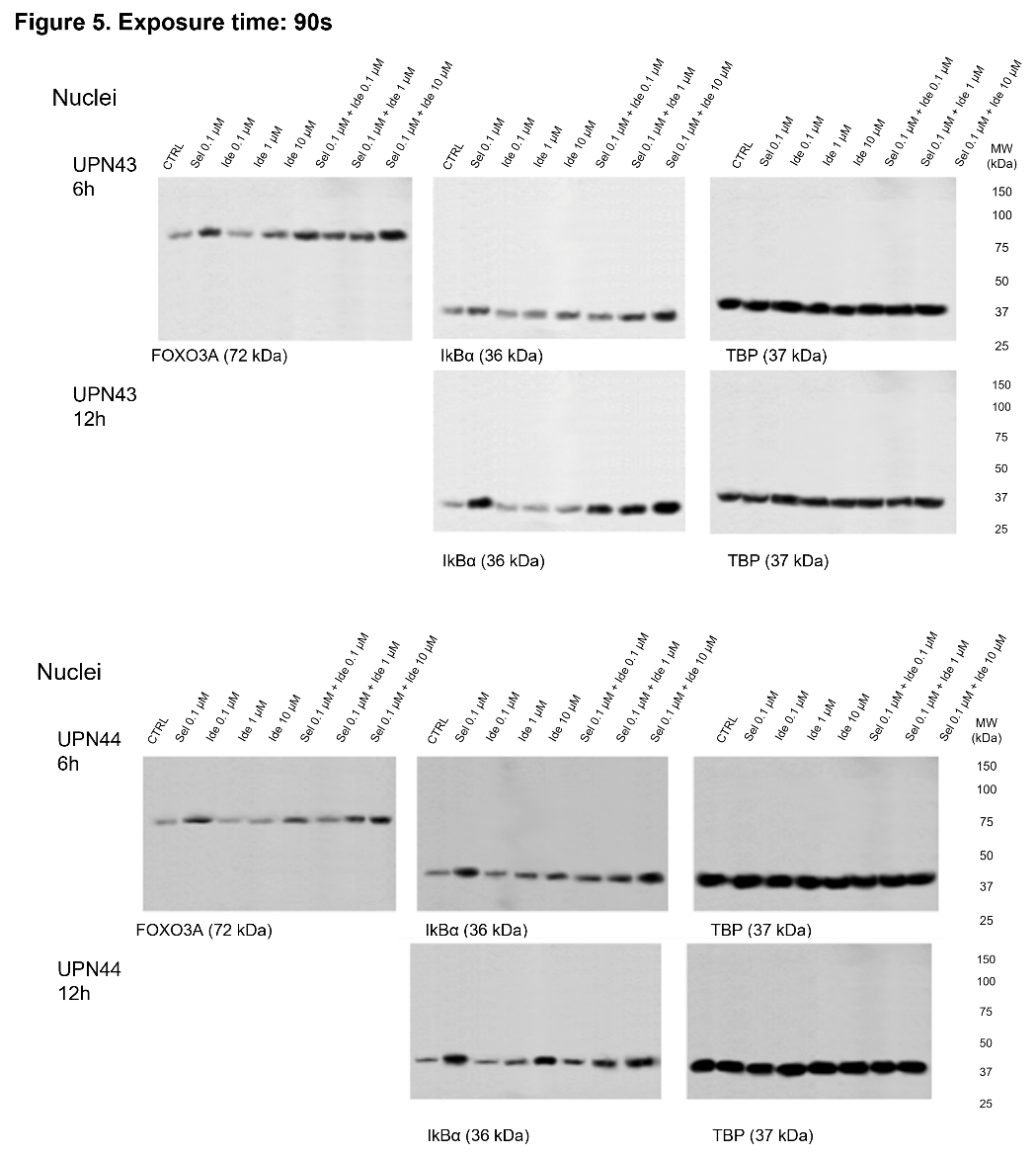


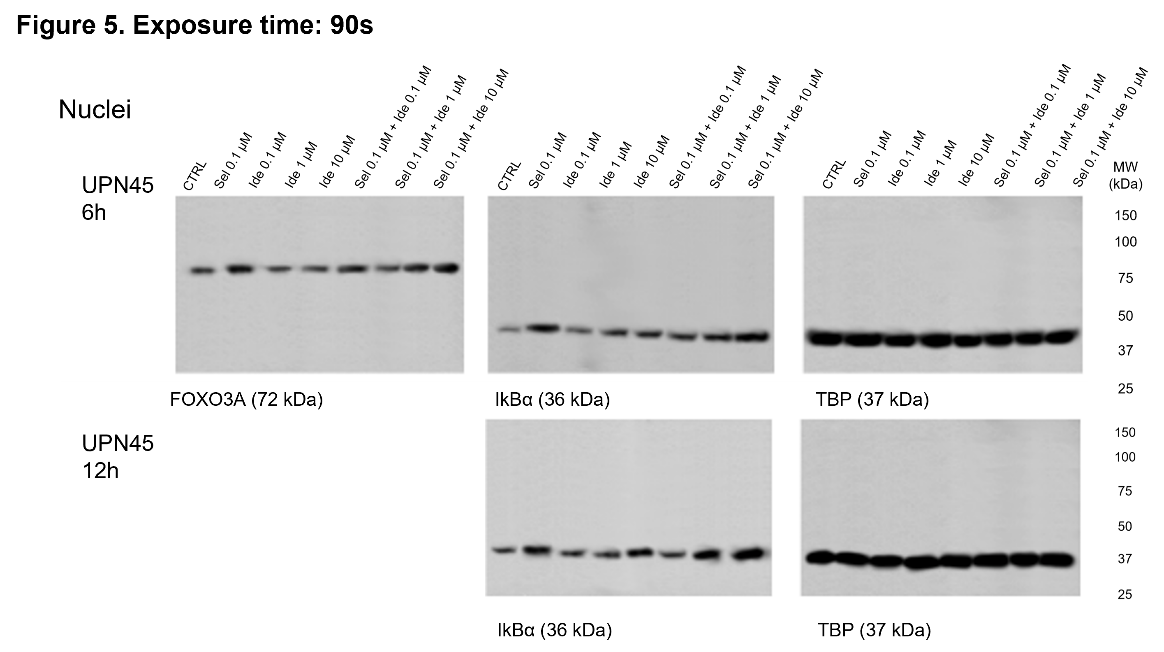


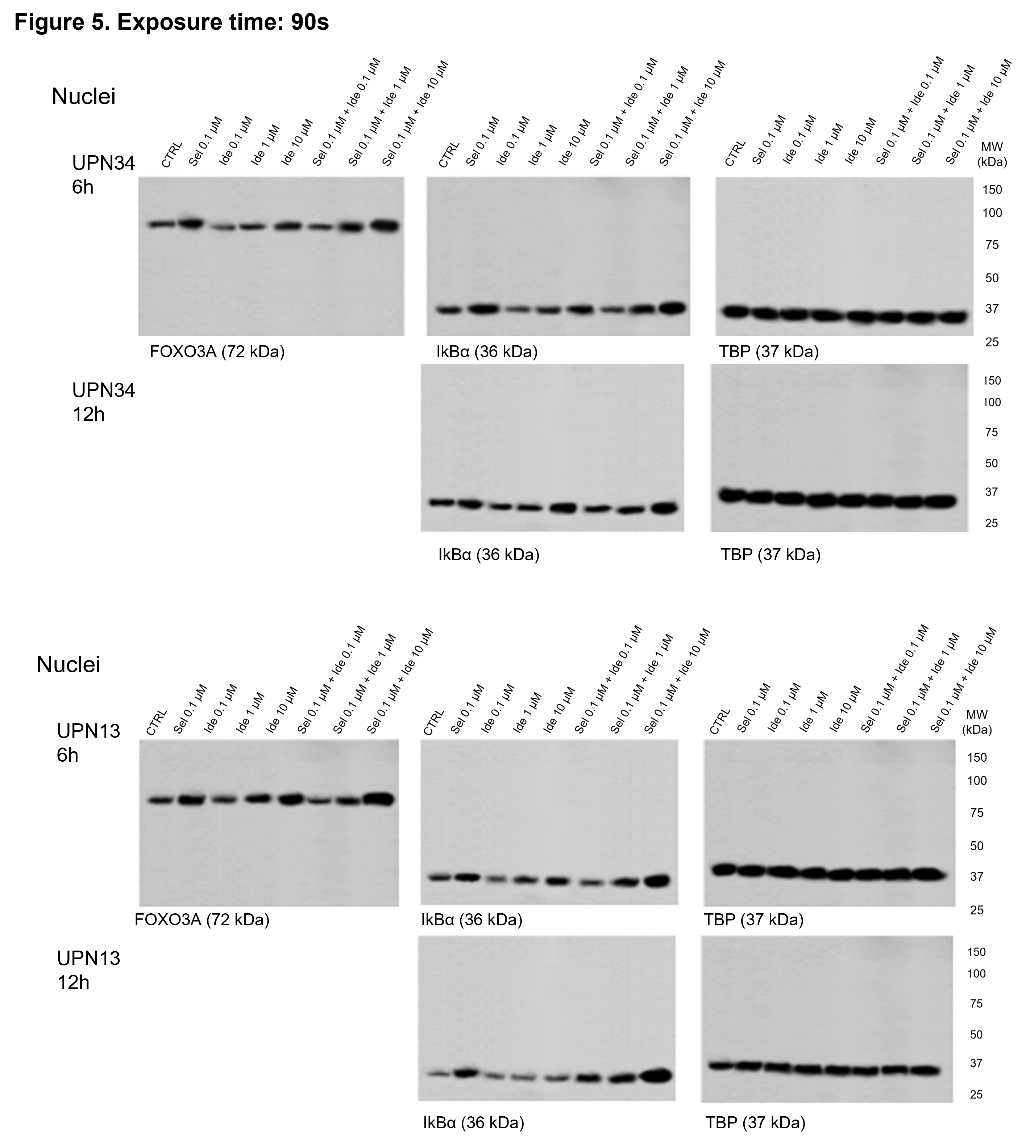


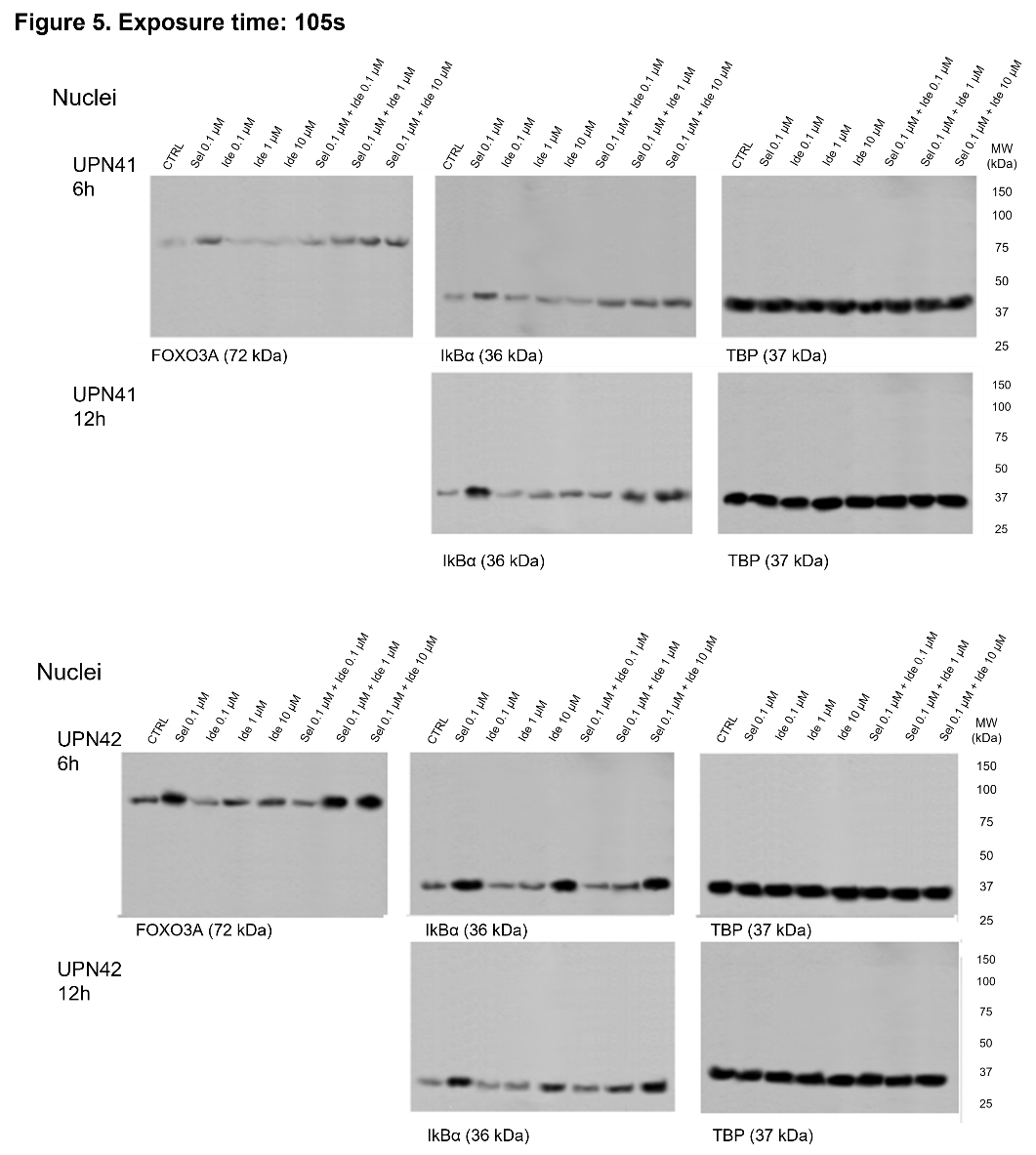

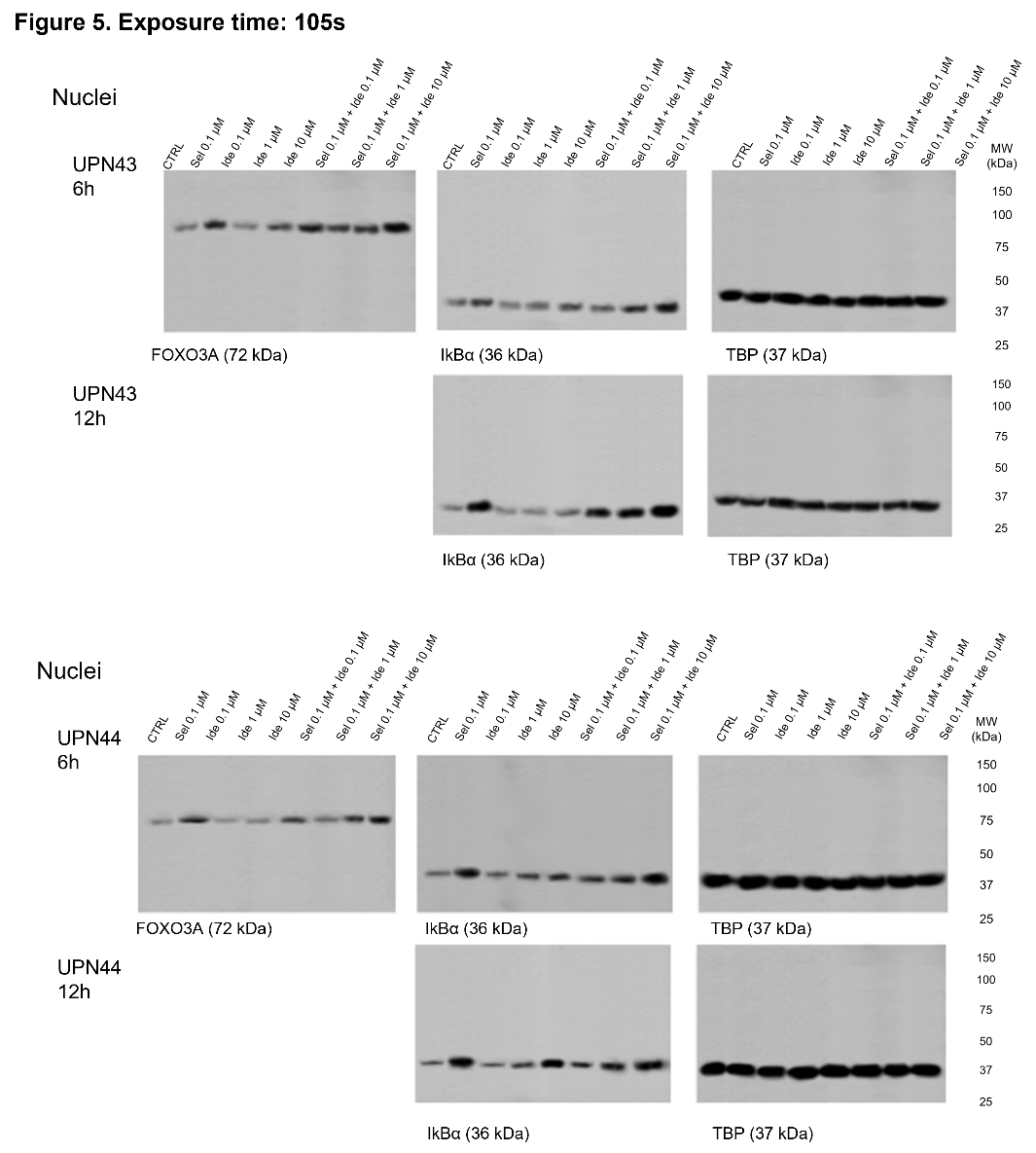


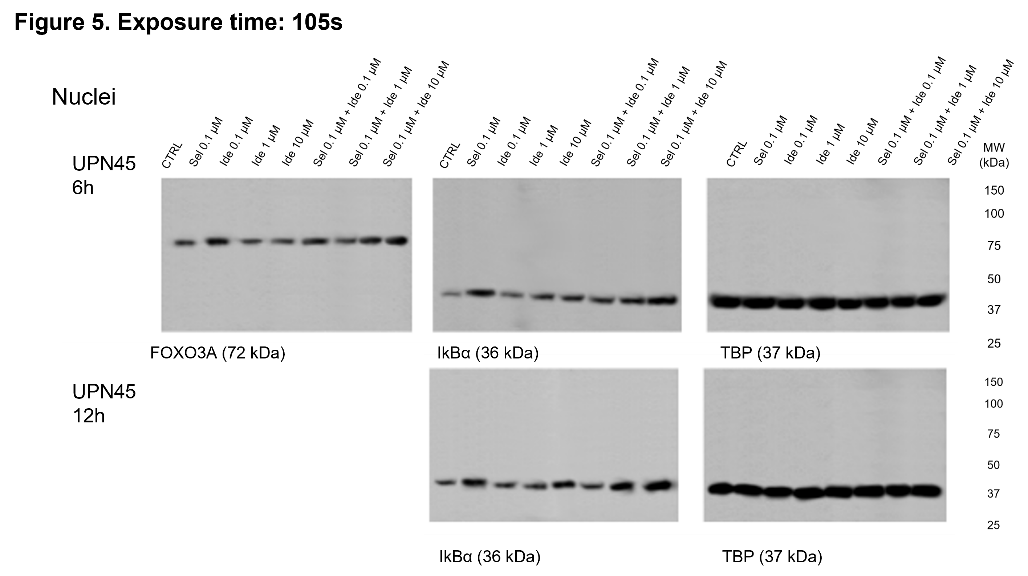

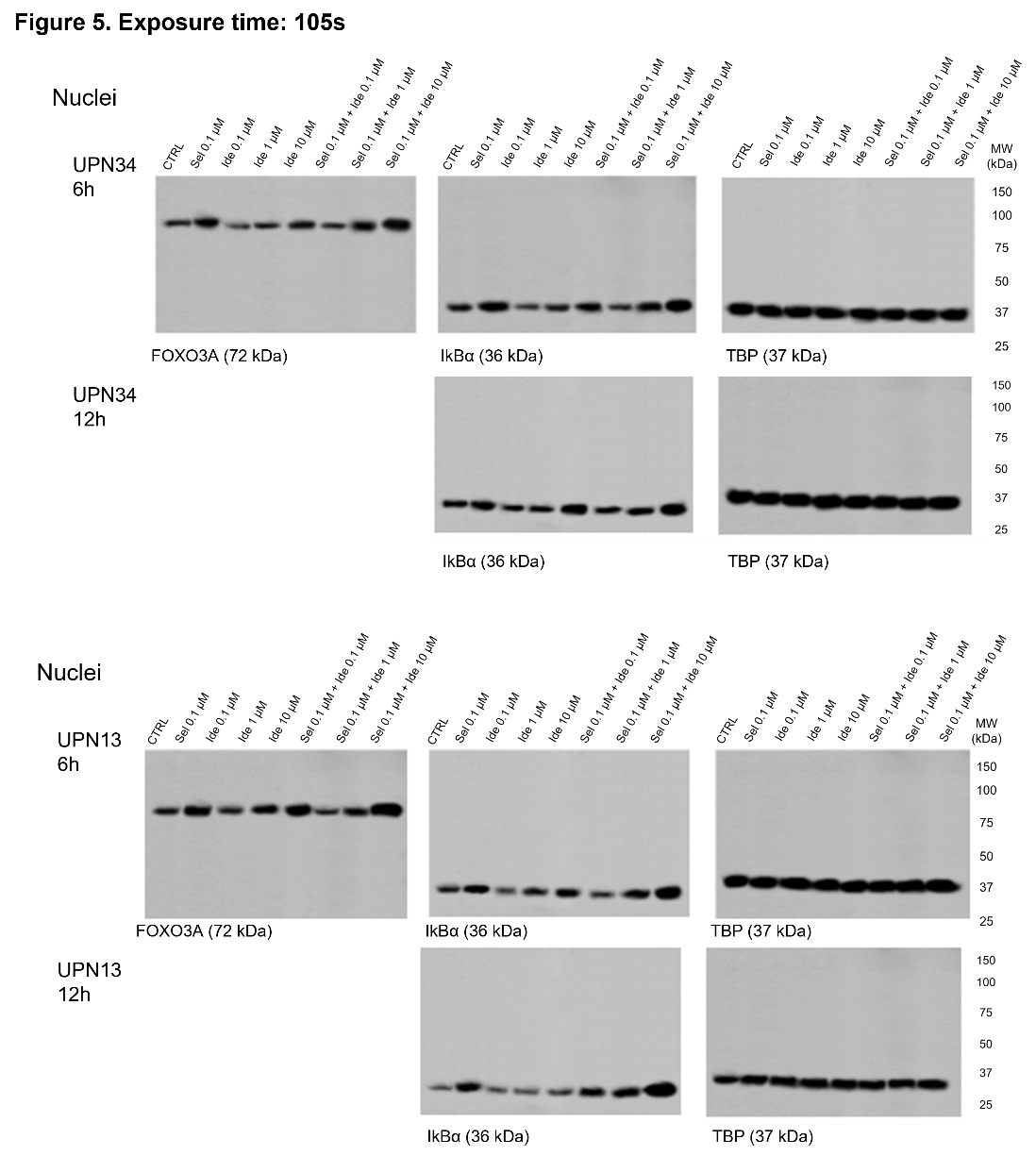


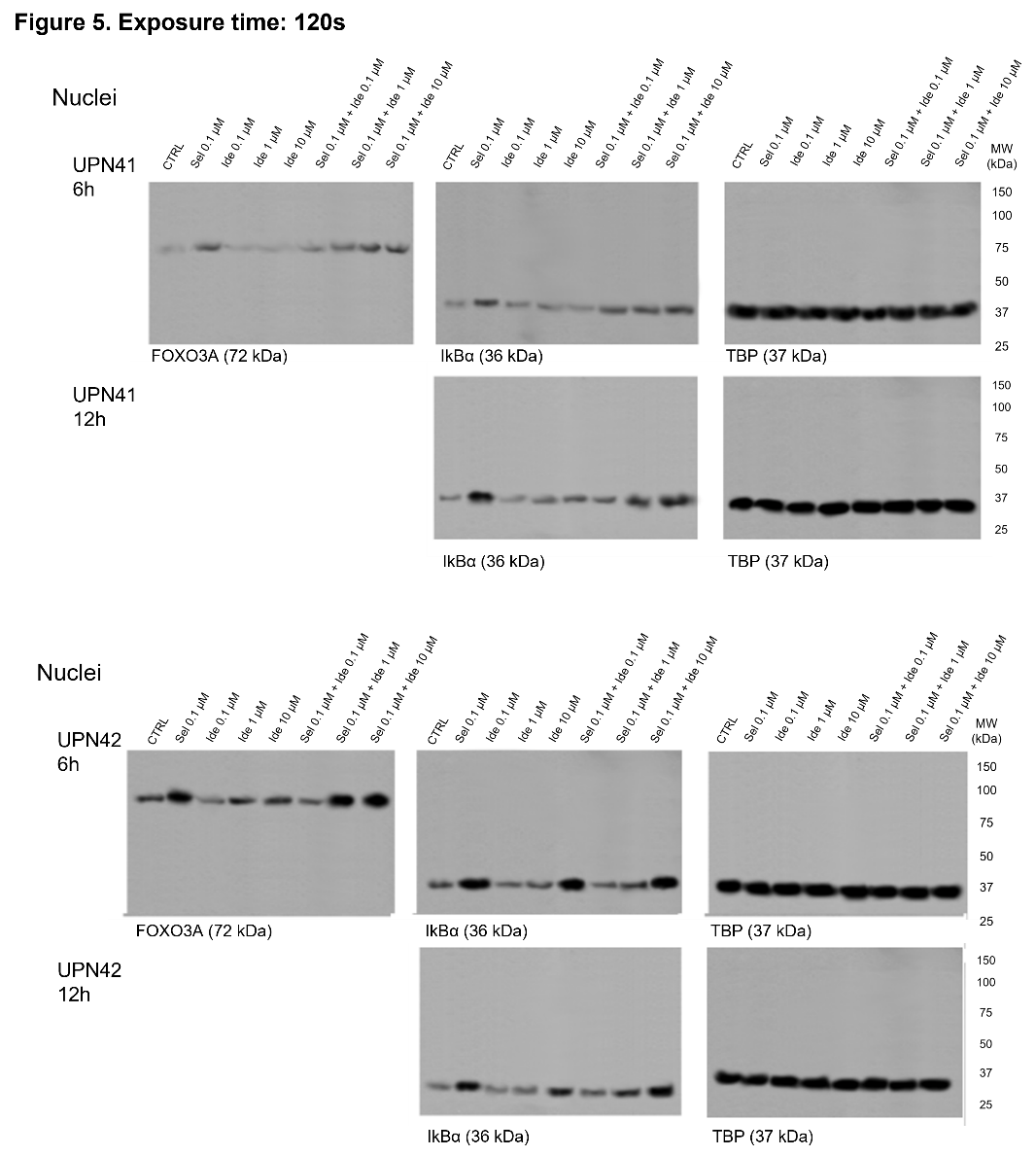

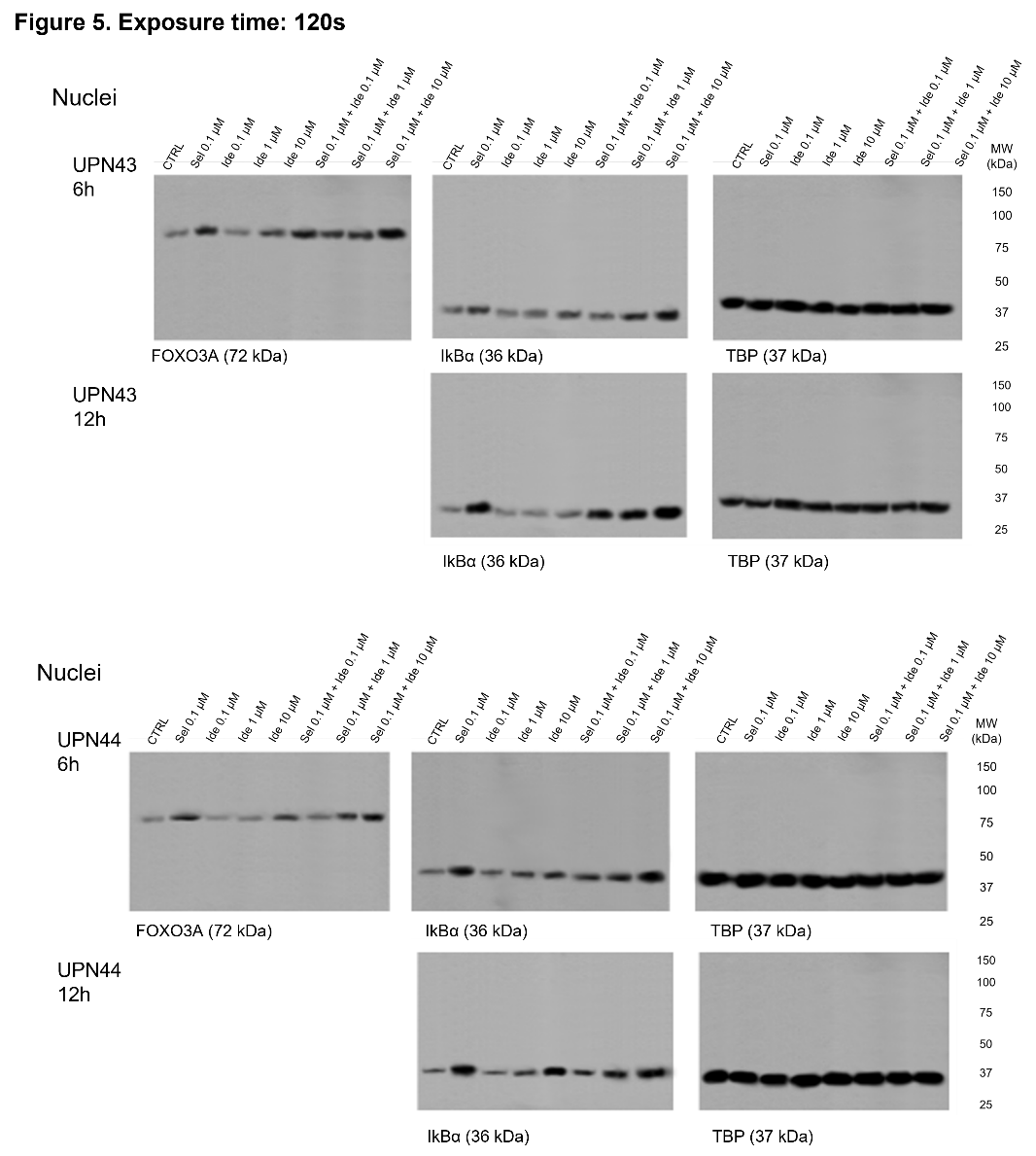


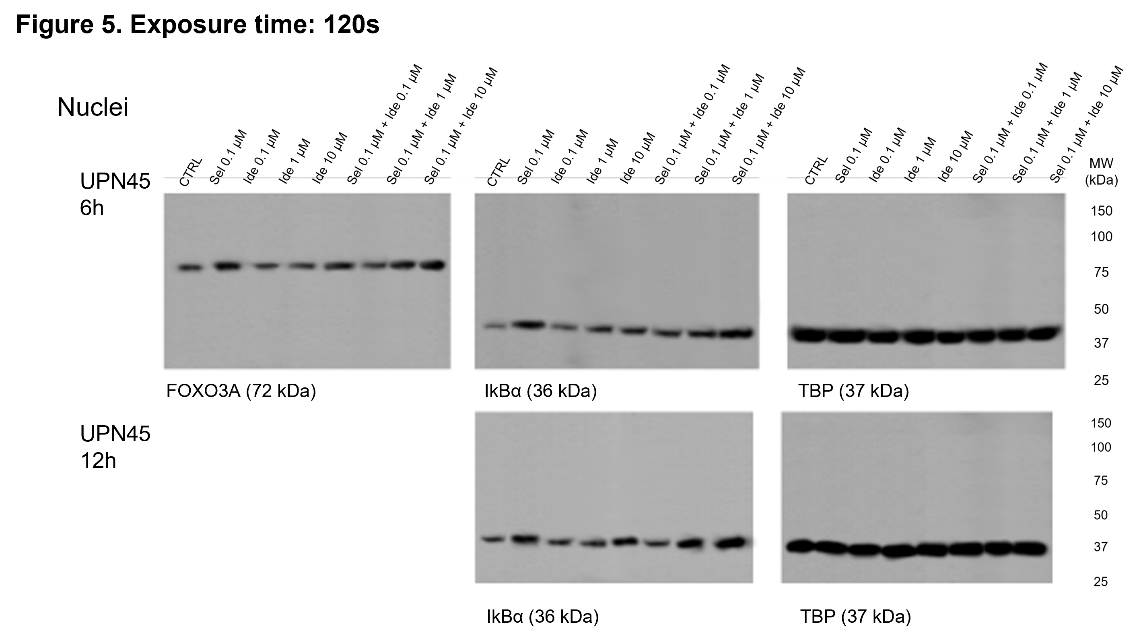

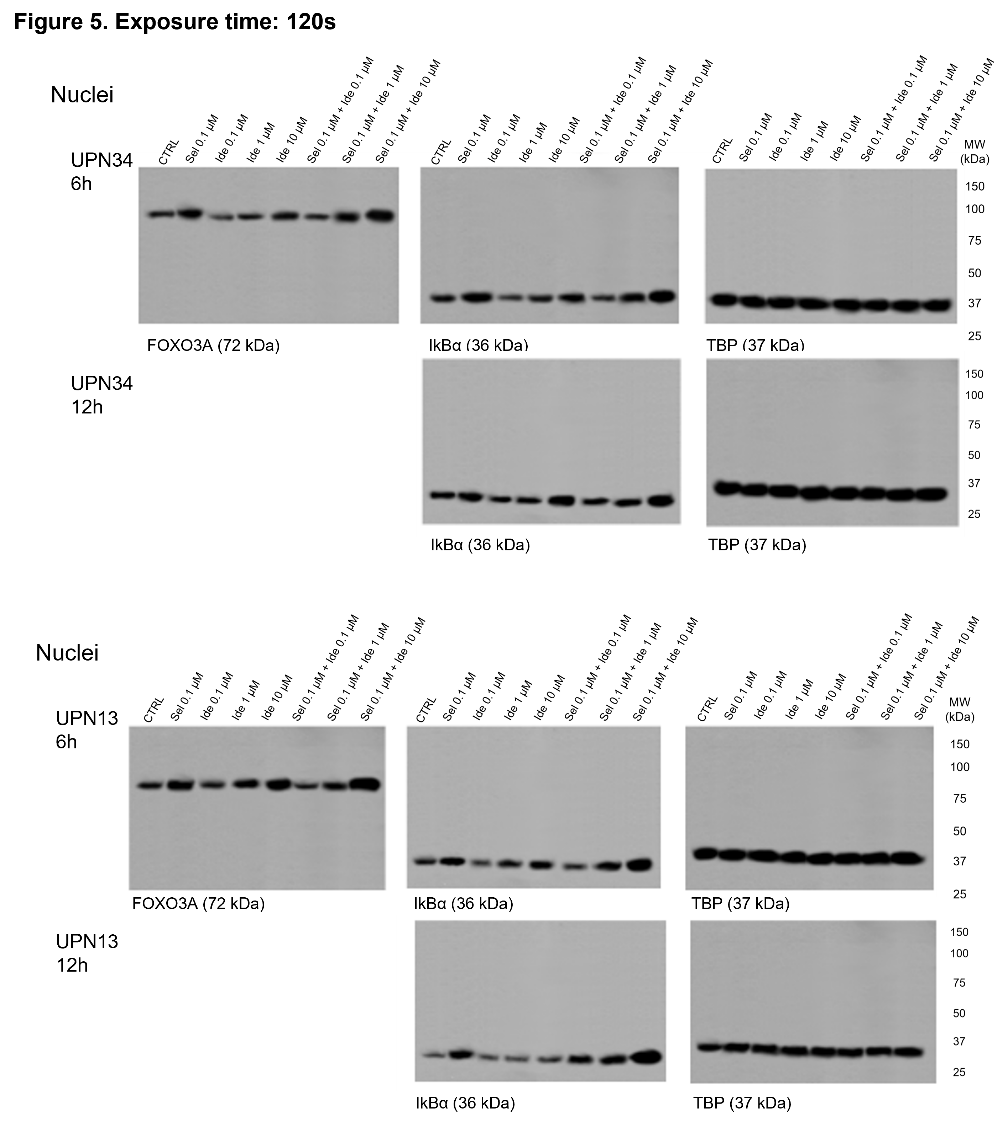


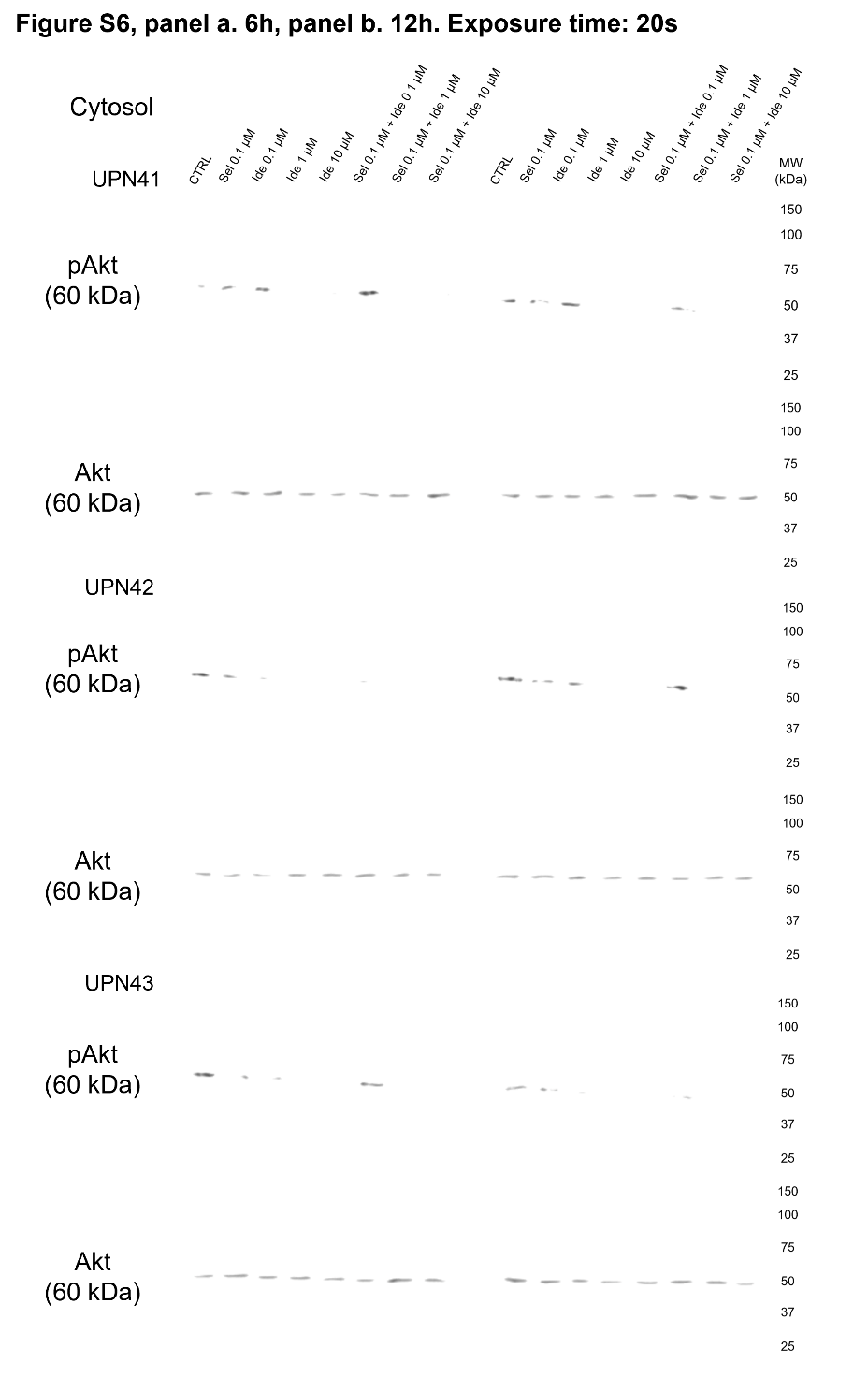

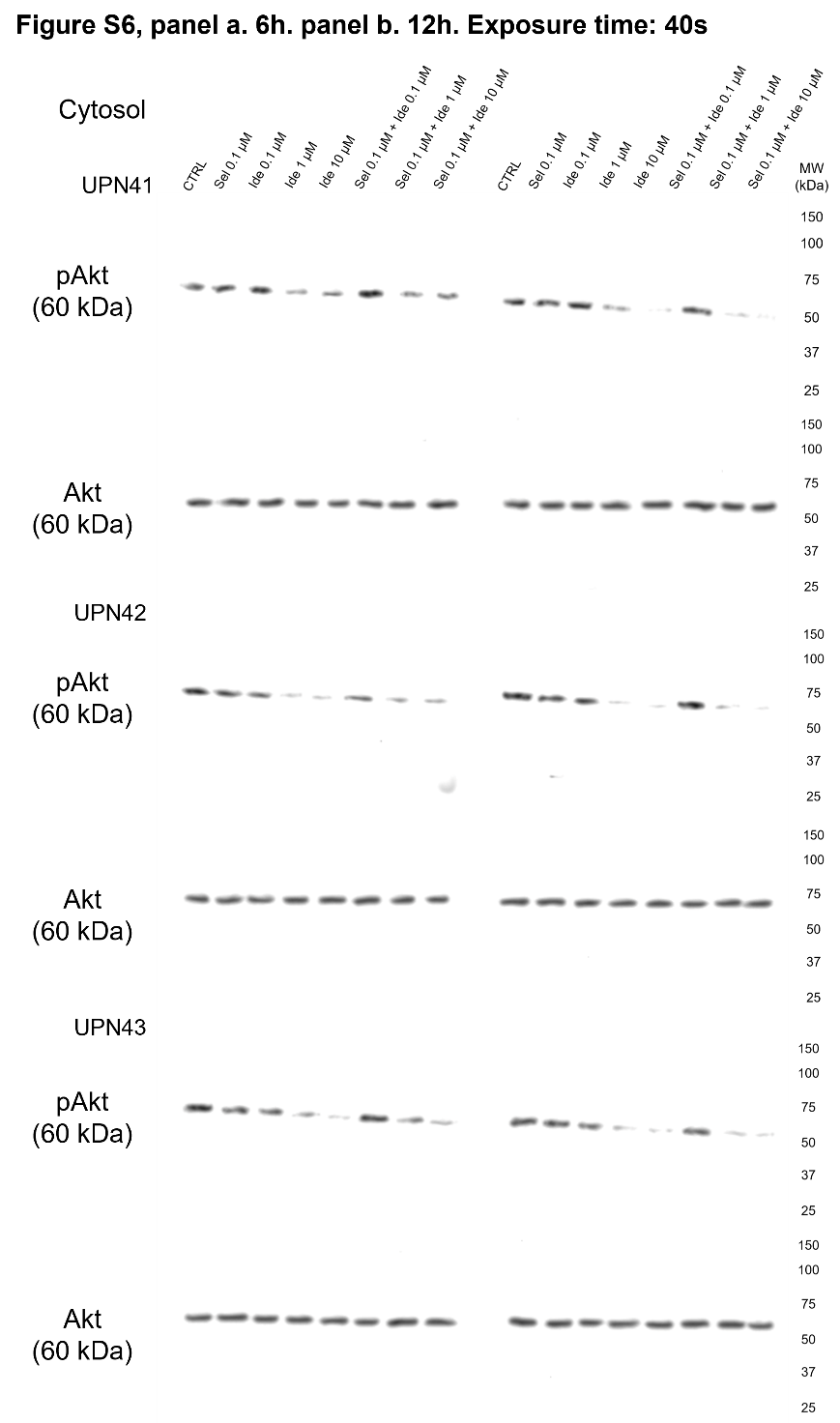


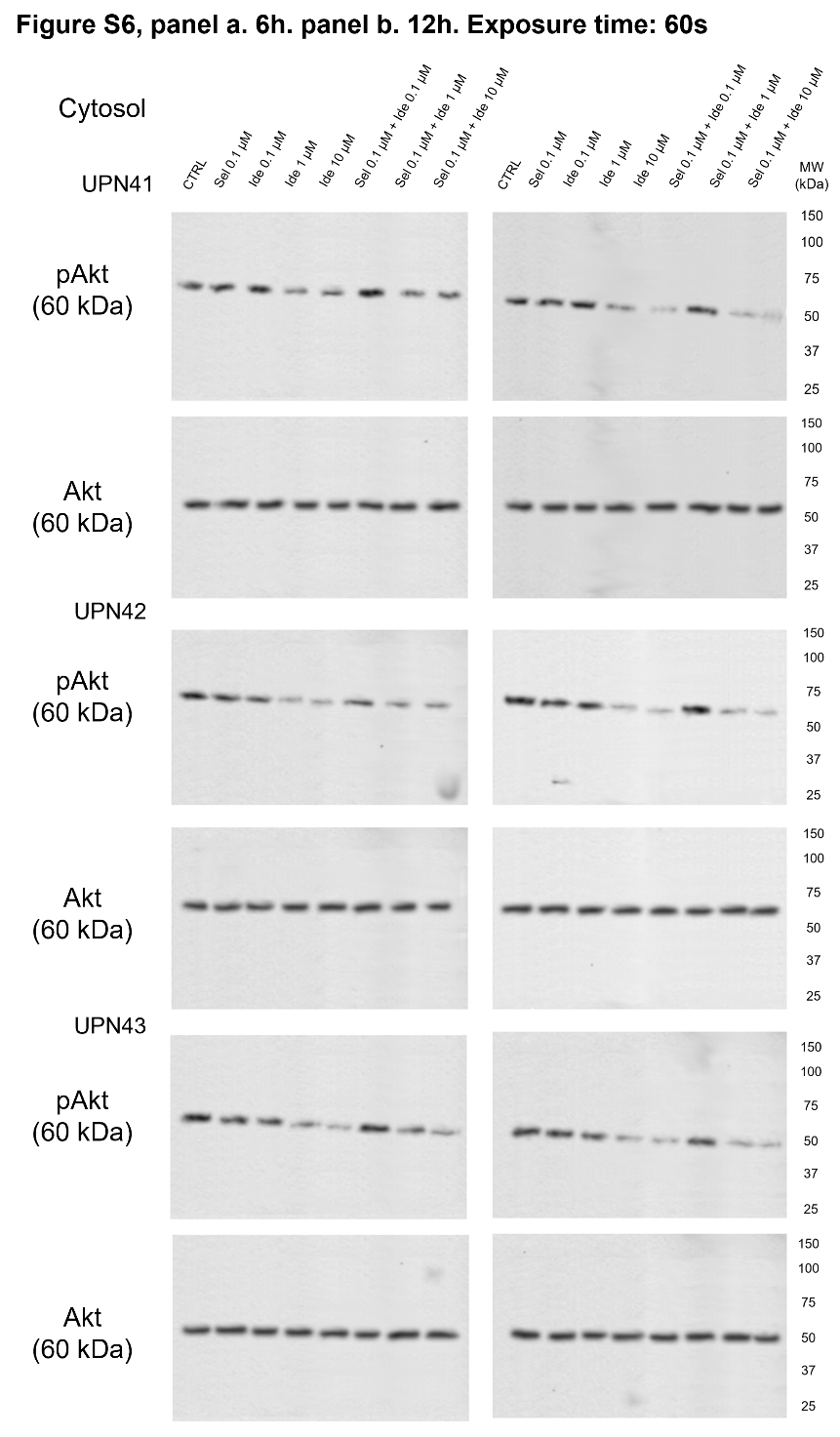

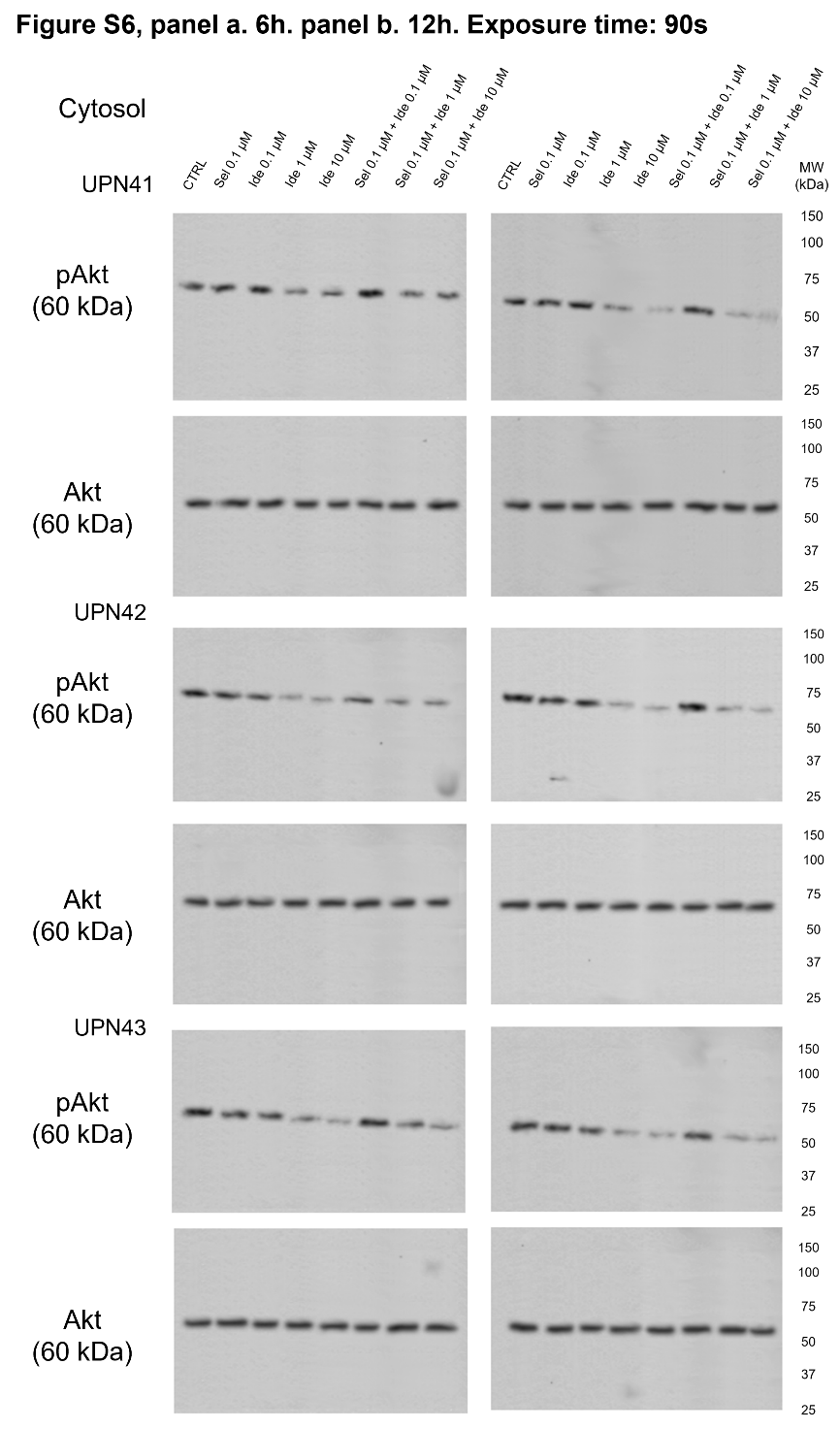


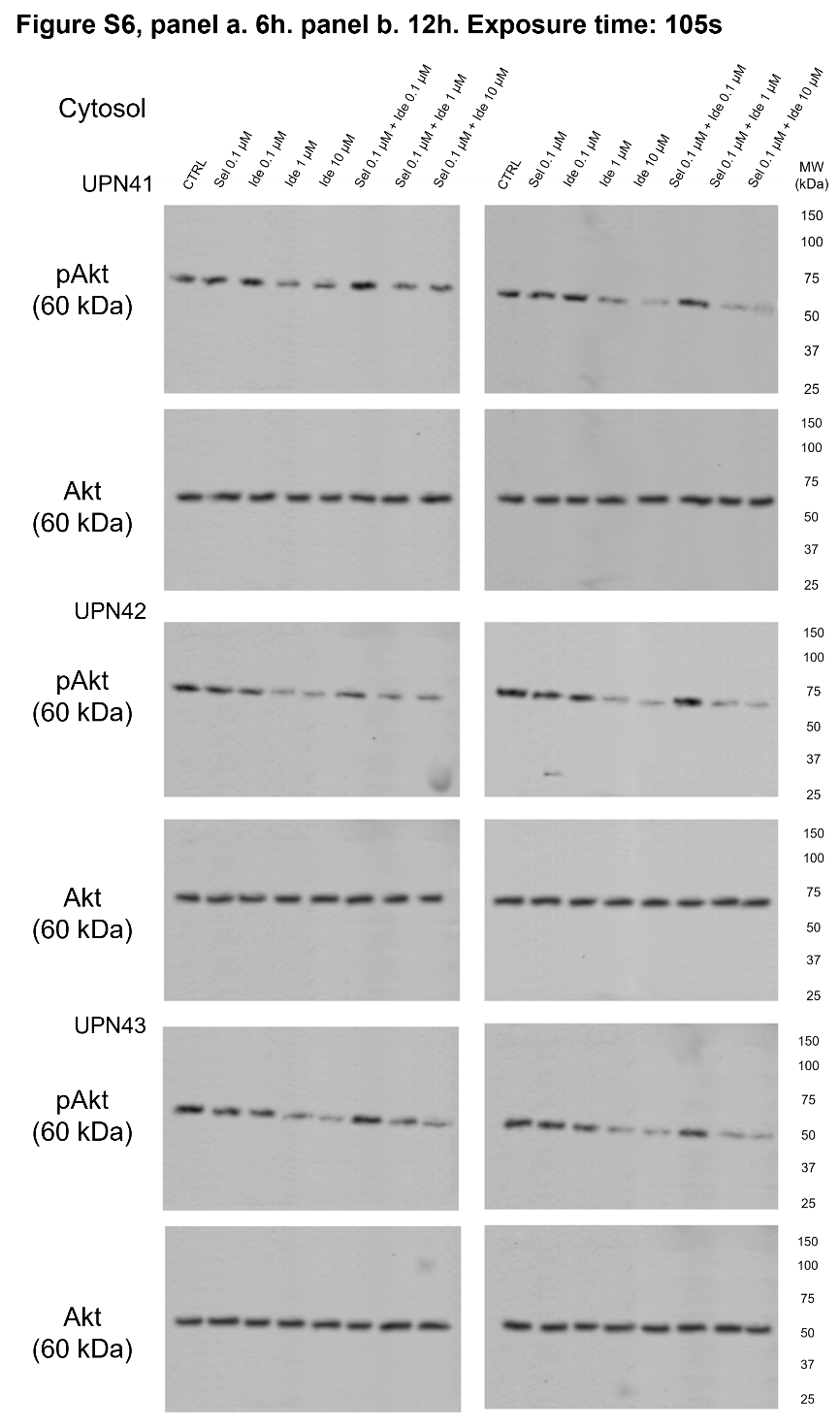

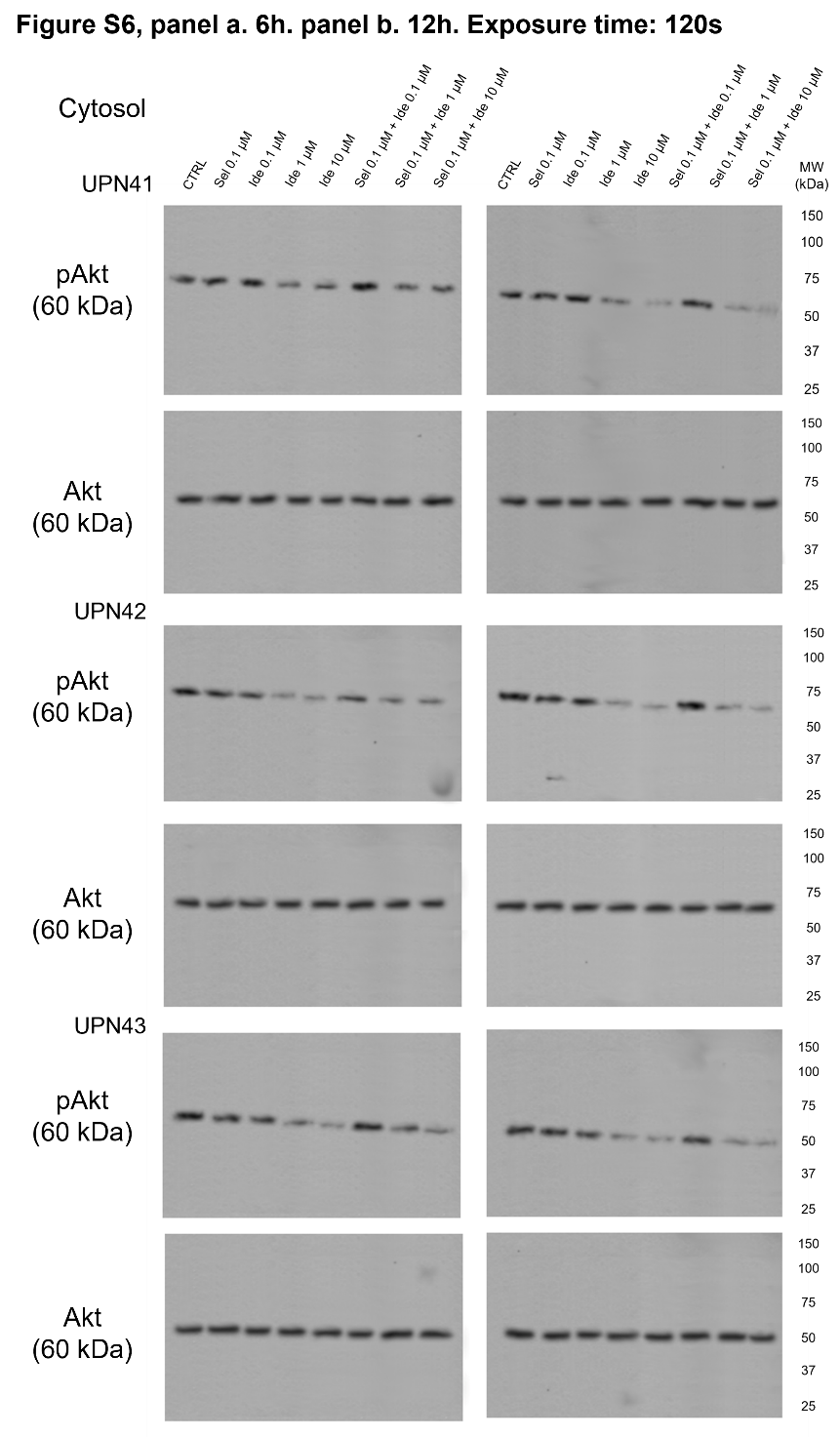


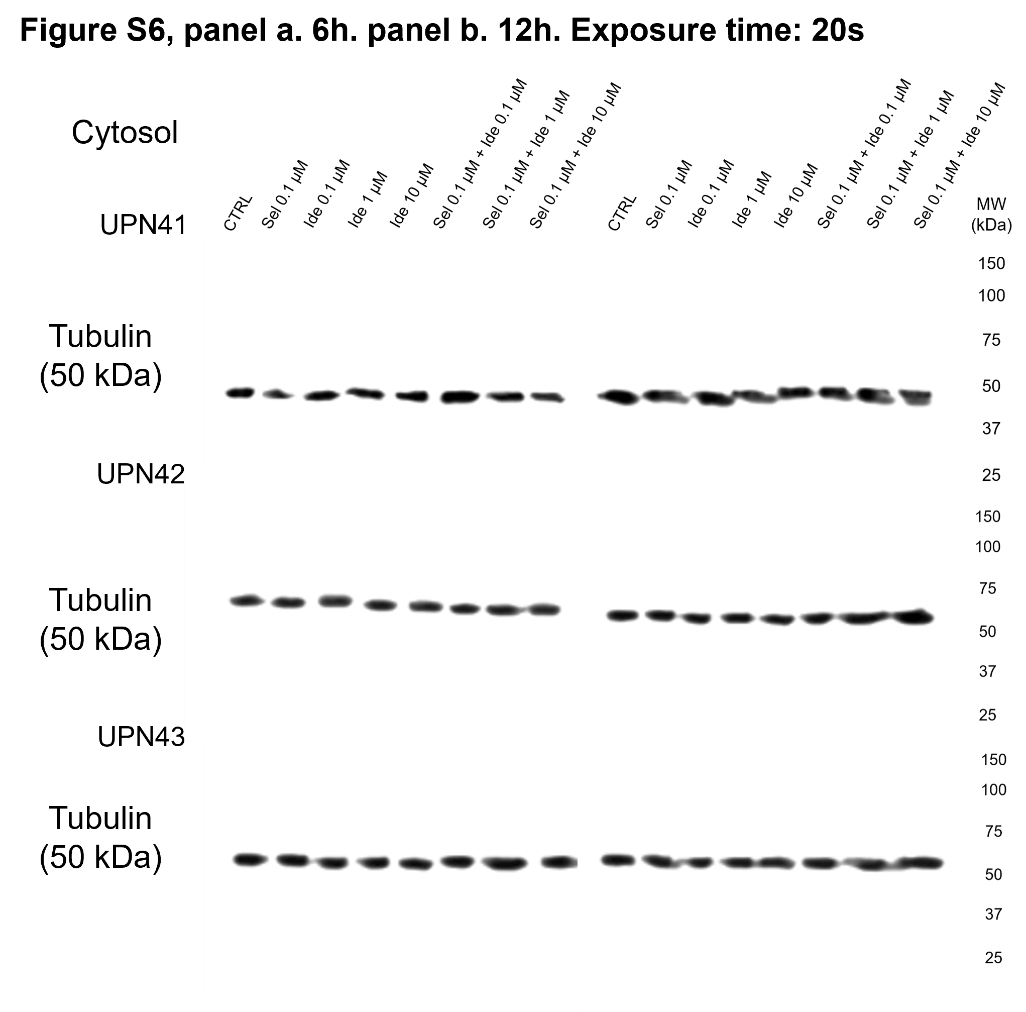

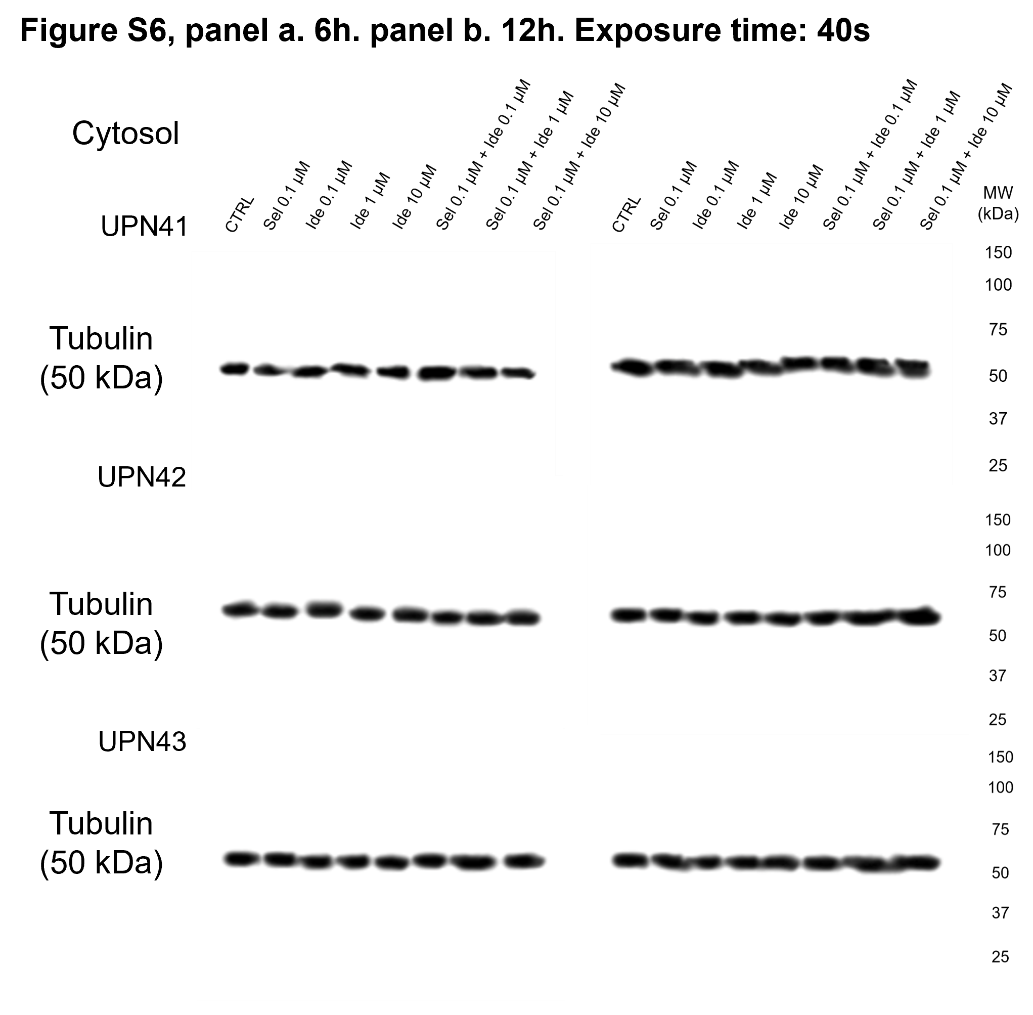


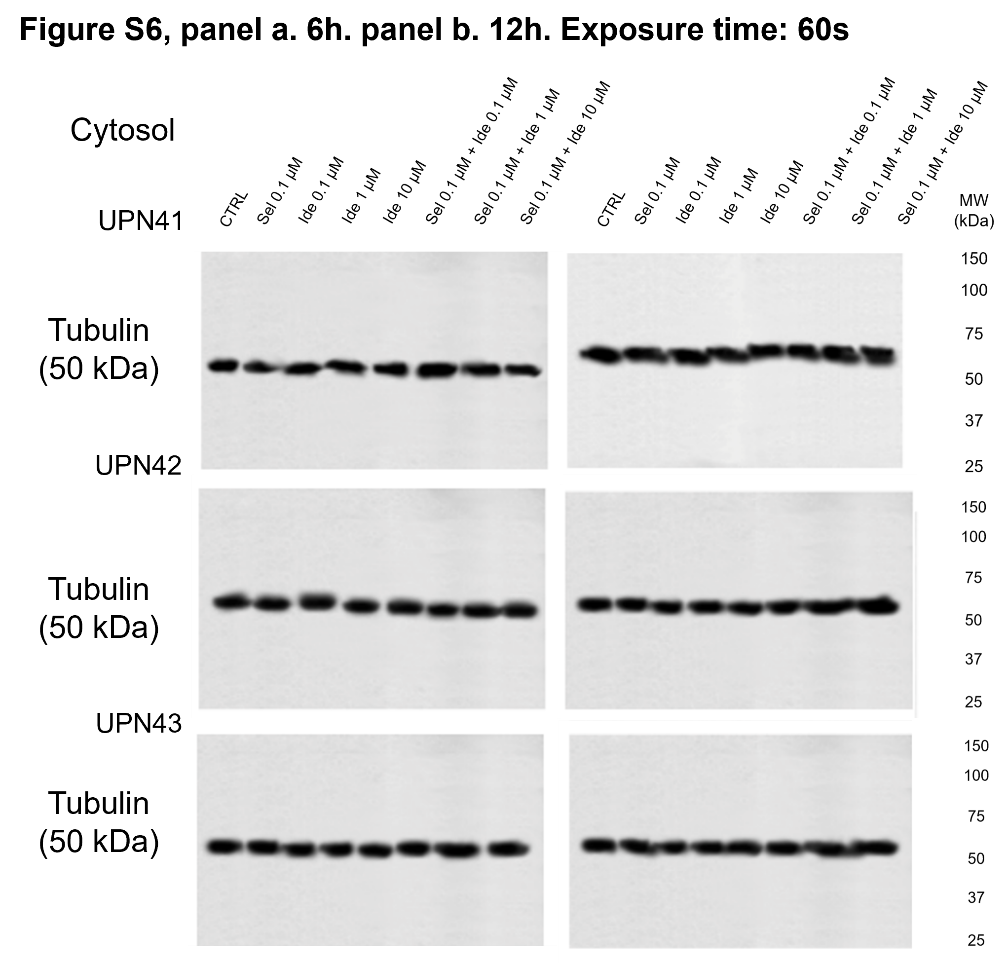

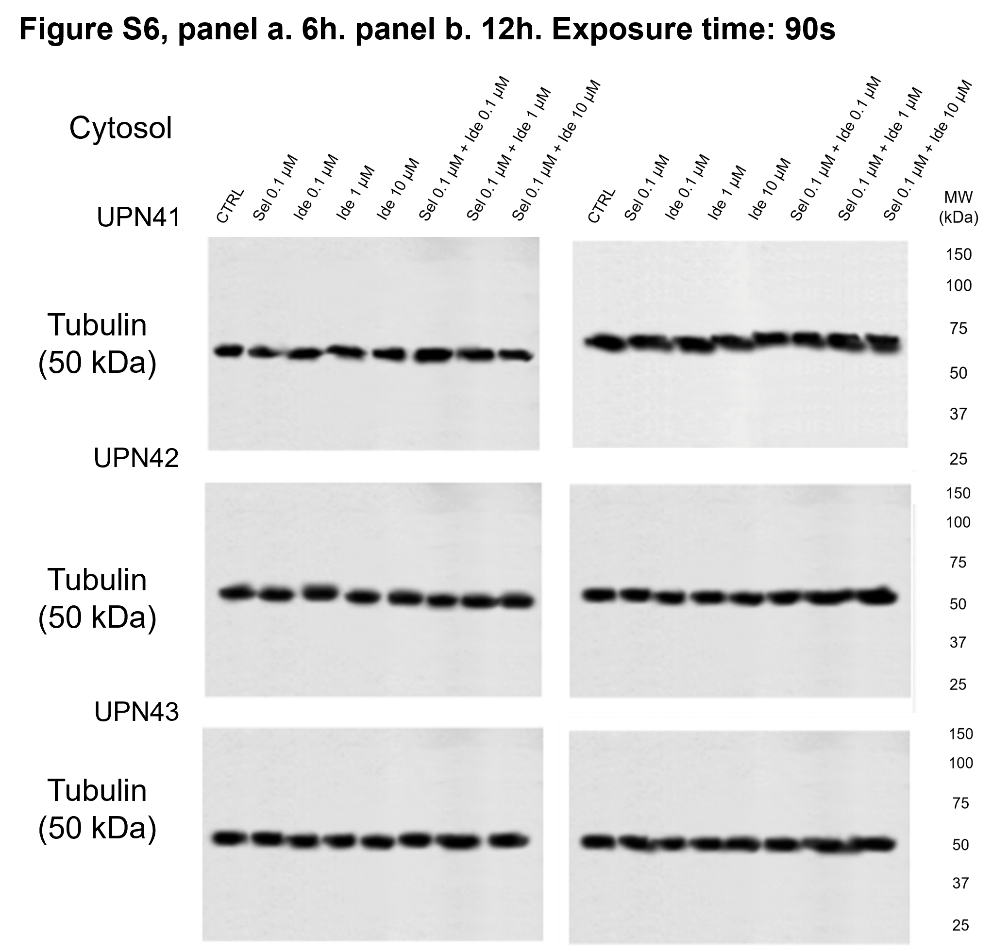


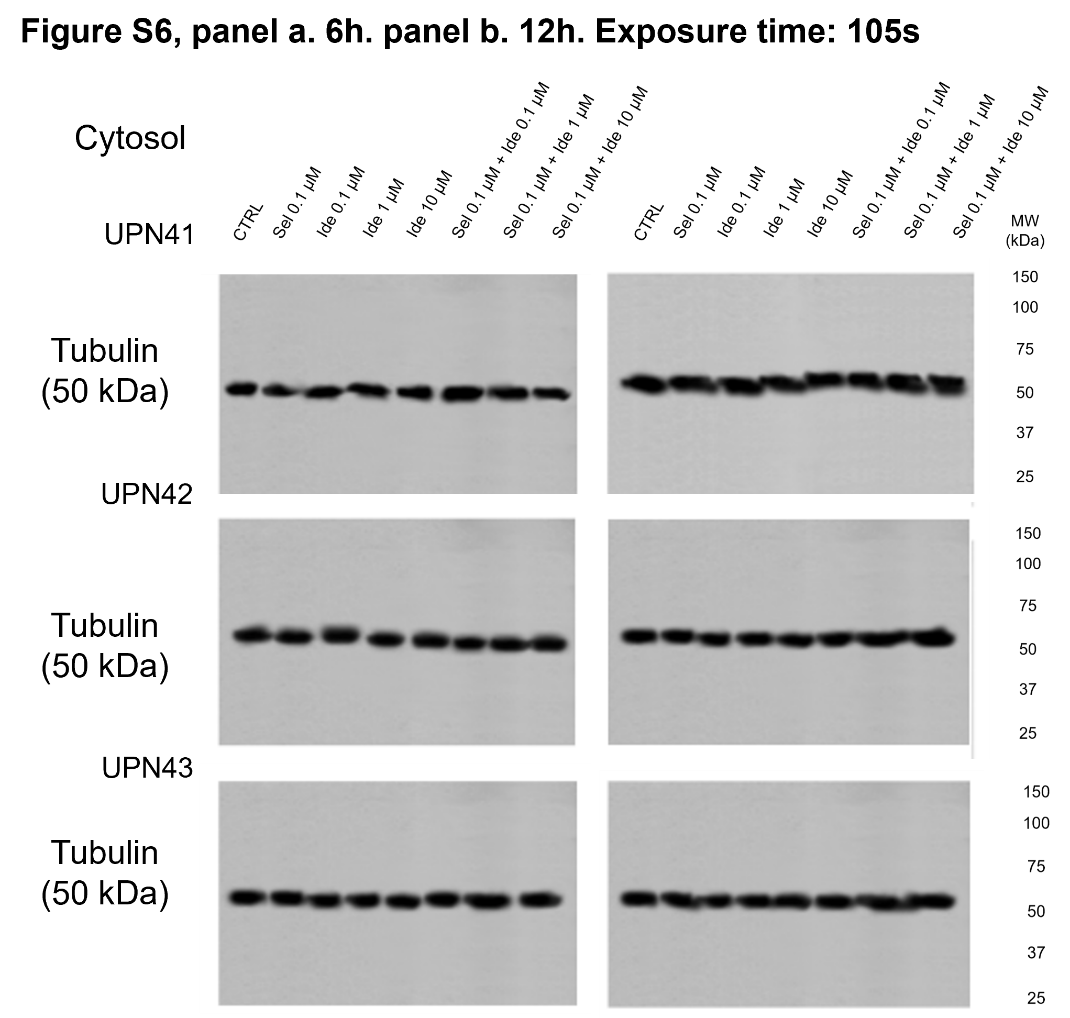

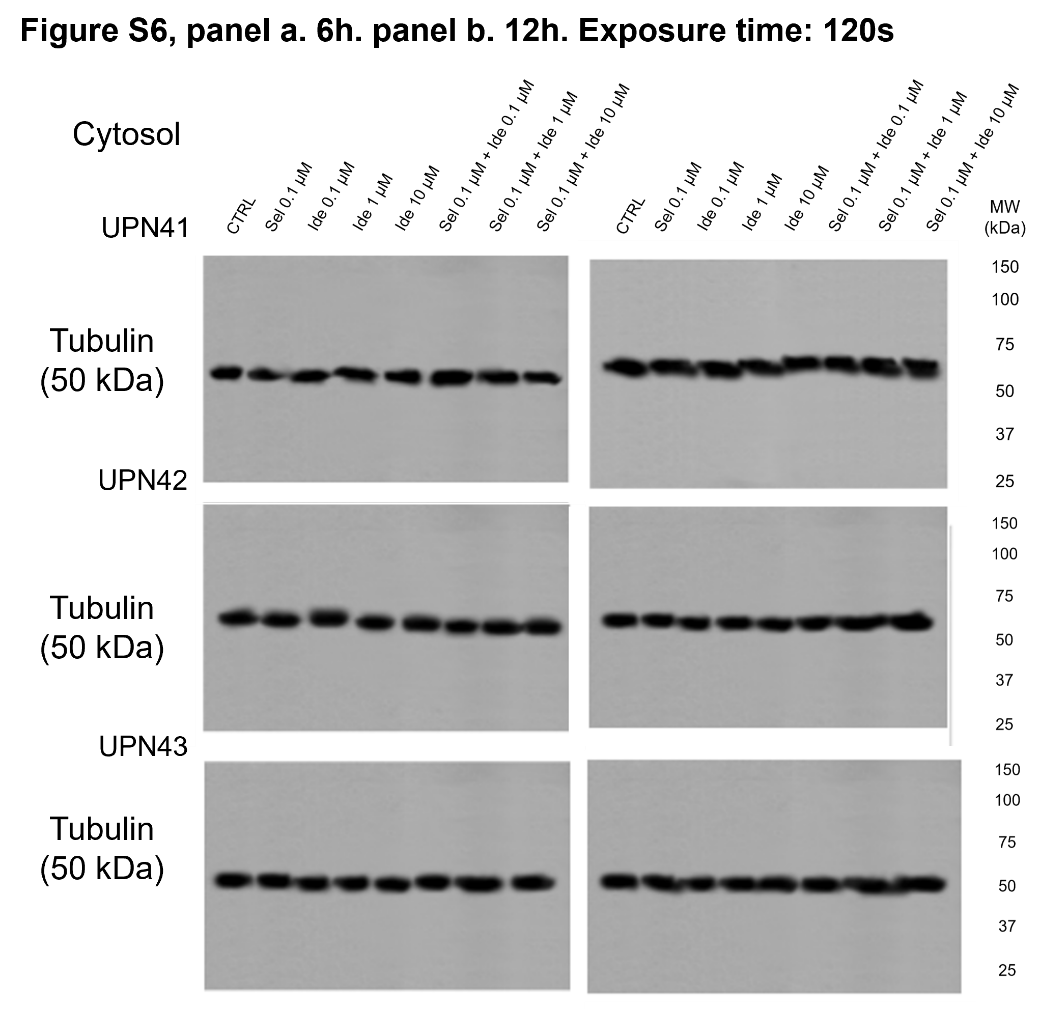


**Supplementary Figure 10. Original whole western blot images acquired at multiple exposure times**

Primary CLL cells were exposed to selinexor (Sel) and idelalisib (Ide) at specified concentrations. IkBα and TBP nuclear amount was evaluated after 6 and 12 hours. FOXO3A nuclear amount was evaluated after 6 hours. Original whole western blots of 7 independent experiments (Unique Patient Number, UPN41, UNP42, UPN43, UPN44, UPN34, UPN13 and UPN45) acquired at indicated times of exposure are shown. Images acquired at 120 s were included in Figure 5 (panels a, c and e). pAkt, Akt and tubulin cytosolic amount was evaluated after 6 and 12 hours. Original whole western blot images of 3 independent experiments (Unique Patient Number, UPN41, UNP42 and UPN43) acquired at indicated times of exposure are reported. Images acquired at 120 s were included in Figure S6 (panels a and b).
